# Role of Gonadotropin Regulated Testicular RNA Helicase (GRTH/DDX25) on Polysomal Associated mRNAs in Mouse Testis

**DOI:** 10.1371/journal.pone.0032470

**Published:** 2012-03-30

**Authors:** Chon-Hwa Tsai-Morris, Hisashi Sato, Ravi Gutti, Maria L. Dufau

**Affiliations:** Section on Molecular Endocrinology, Program in Developmental Endocrinology and Genetics, Eunice Kennedy Shriver National Institute of Child Health and Human Development, National Institutes of Health, Bethesda, Maryland, United States of America; National University of Singapore, Singapore

## Abstract

Gonadotropin Regulated Testicular RNA Helicase (GRTH/Ddx25) is a testis-specific multifunctional RNA helicase and an essential post-transcriptional regulator of spermatogenesis. GRTH transports relevant mRNAs from nucleus to cytoplasmic sites of meiotic and haploid germ cells and associates with actively translating polyribosomes. It is also a negative regulator of steroidogenesis in Leydig cells. To obtain a genome-wide perspective of GRTH regulated genes, in particularly those associated with polyribosomes, microarray differential gene expression analysis was performed using polysome-bound RNA isolated from testes of wild type (WT) and GRTH KO mice. 792 genes among the entire mouse genome were found to be polysomal GRTH-linked in WT. Among these 186 were down-regulated and 7 up-regulated genes in GRTH null mice. A similar analysis was performed using total RNA extracted from purified germ cell populations to address GRTH action in individual target cells. The down-regulation of known genes concerned with spermatogenesis at polysomal sites in GRTH KO and their association with GRTH in WT coupled with early findings of minor or unchanged total mRNAs and abolition of their protein expression in KO underscore the relevance of GRTH in translation. Ingenuity pathway analysis predicted association of GRTH bound polysome genes with the ubiquitin-proteasome-heat shock protein signaling network pathway and NFκB/TP53/TGFB1 signaling networks were derived from the differentially expressed gene analysis. This study has revealed known and unexplored factors in the genome and regulatory pathways underlying GRTH action in male reproduction.

## Introduction

The testis contains diverse cell populations including somatic Leydig/ Sertoli cells and germ cells (undifferentiated diploid spermatogonia, meiotic spermatocyte, haploid spermatid and spermatozoa). Leydig cells present in the interstitial cells compartment adjacent to the seminiferous tubules contain the steroidogenic enzymes and steroid precursors to produce androgens that are essential for germ cells development. Sertoli cells located at basal sites of seminiferous tubules, act as nurse cells of the developing sperm cells. Testicular function is controlled primarily by pituitary gonadotropins (LH/FSH) that bind to specific gonadal receptors (LH receptor in Leydig cells FSH receptor in Sertoli cells) to regulate steroid production and gametogenesis [1], [2]. In conjunction with FSH, androgens stimulate the production of proteins and other factors essential for germ cells differentiation [3], [4]. The process of germ cells development from spermatogonia into mature spermatozoa depends on the integrated expression of an array of genes in a precise temporal sequence [5], [6]. Two thirds of the mRNAs in the adult mammalian testes are associated with specific proteins as messenger ribonuclear protein (mRNP) complex. mRNAs are transported from nucleus to the cytoplasm where messages are translationally repressed presumably in the chromatoid body of round spermatids and where can also undergo degradation [7], [8], [9], [10]. Translational activation of stored mRNAs transported to polyribosomes at specific times is critical for the progression of spermatogenesis.

Gonadotropin regulated testicular RNA helicase (GRTH/Ddx25) is a testis specific member of the DEAD box family of RNA helicase [9], [10], [11], [12], [13]. It contains 483 aa and shares the 9 conserved signature motifs found in members of the DEAD box family of RNA helicases. This helicase displays ATP binding and hydrolysis, RNA binding and RNA unwinding activities [11]. It is the sole family member to be hormonally regulated. GRTH is regulated by gonadotropin/androgen in Leydig cells and germ cells of the testis [12], [13] where its expression is both cell- and stage-specific. It is highly expressed in pachytene and metaphase spermatocytes and round spermatids, where it regulates the expression of crucial proteins in sperm maturation including H4, HMG2, TP1, TP2, PGK2, ACE and protamines 1 and 2 [13], [14]. As a component of messenger ribonucleoprotein particles, GRTH participates in the transport of specific mRNAs to cytoplasmic sites (Chromatoid body of round spermatids) for storage of mRNAs and prior to their translation at specific times during spermatogenesis [13], [14], [15]. Also, through its association with polyribosomes GRTH may regulate the translation of messages encoding spermatogenic factors [14]. GRTH null mice are sterile and lack sperm due to the failure of round spermatids to elongate, resulting in complete arrest at step 8 of spermiogenesis [13]. There is also a major decrease in size of the chromatoid body in GRTH KO mice, consistent with the marked reduction of nuclear-cytoplasmic transport of messages relevant to spermiogenesis, presumably stored in these organelles [14], [15].

In Leydig cells, GRTH has been recently demonstrated to regulate the expression of genes involved in cholesterol synthesis and transfer (SREBP2, HMG-CoA and StAR) [16]. This helicase regulates cholesterol availability at the mitochondrial level through its negative role on StAR message stability. This consequently impacts the cholesterol transport to the inner mitochondrial membrane which is essential for pregnenolone synthesis and ultimately androgen production. GRTH is the first helicase reported to display a novel negative autocrine control of the androgen production in the male.

Since GRTH is a multifunctional RNA helicase that is an essential post-transcriptional regulator of spermatid development and the completion of spermatogenesis and has important role in the regulation of steroid synthesis, it is important to obtain a genome-wide analysis of messages associated with GRTH in different types of testicular cells, and their associations with polyribosomes, to gain further insights into GRTH action. Comparison of genes associated with actively translating polysomes isolated by sucrose gradient of testis extracts from wild type and GRTH knockout mice was performed to obtain differential gene profiles regulated by GRTH. Among them, messages associated with GRTH were identified from polysomes of WT mice testes immunoprecipitated by GRTH antibody. Subsequently differential mRNA expression profile of purified testicular cells extracts was also examined to determine specific GRTH regulation in Leydig and germ cells. A bioinformatic approach utilizing Ingenuity pathway software was further applied to predict the potential network signaling pathways to obtain a global aspect of GRTH regulation. The differential set of genes derived from the present study serve as foundation for the understanding of the molecular mechanism of spermatogenesis under GRTH control in the testis.

## Materials and Methods

### Animals

Adult GRTH wild type and GRTH^−/−^ male mice were housed in temperature and light-controlled conditions. All animal studies were approved by the National Institute of Child Health and Human Development Animal and Care and Use Committee. Animals were killed by asphyxiation with CO_2_ and decapitated. Testes were removed and decapsulated for total testicular RNA extraction, polysome preparation or testicular cells preparation. Leydig cells (interstitium) and germinal cells (seminferous tubule) were prepared (see below) and further purified for RNA extraction.

### Testicular Cells Preparation

Leydig cells were prepared by collagenase dispersion and purified by centrifugal elutriation [17]. Testicular germ cells (round spermatid and spermatocyte) were prepared by collagenase/trypsin dispersion and purified by centrifugal elutriation [12], [18]. After collagenase dispersion, seminiferous tubules were minced and incubated in Medium 199 containing 0.1% bovine serum albumin, 0.1% trypsin (Sigma), and 17 µg/ml DNase (Sigma) for 15 min in a rotary water bath (80 rpm, 35°C). After the addition of soybean 0.04% trypsin inhibitor, the sample was filtered through a 300-, 90-, 40-µm mesh screen, and glass wool and cells were pelleted and re-suspended in elutriation buffer containing 2 µg/ml DNase. Spermatocytes were subsequently separated and purified by centrifugal elutriation using Beckman Avanti 21B centrifuge with elutriator rotor model J 5.0 as described previously. The first 2 fractions (1 and 2) were collected with flow rates of 31.5 and 41.4 ml/min at 3000 rpm, and 2 additional fractions (3 and 4) were obtained with flow rates of 23.2 and 40 ml/min at 2000 rpm. Cells were identified on air-dried smears, fixed in Bouin's fixative, and stained with hematoxylin and periodic acid-Schiff. Fractions 2 and 4 contain round spermatids and pachytene spermatocytes at a purity of 84 and 86%, respectively. The purification of germ cells is assessed based on the morphology of different germ cells types and in case of Leydig cells on the functional validation and histochemical staining for 3ß-hydroxysteroid dehydrogenase using nitro- blue tetrazolium [17].

### Polysome preparation

Polysomes were isolated using sucrose-gradient fractionation essentially as previously described [14]. Briefly, mouse testes were homogenized in hypotonic buffer A (25 mM Tris, pH 7.5, 100 mM KCL, 5 mM MgCl_2_, 1 mM dithiothreitol) containing protease inhibitors and cycloheximide (100 µg/ml) using a Dounce homogenizer. Lysates were centrifuged at 500× *g* for 5 min to separate nuclear from cytoplasmic fraction. Sucrose gradients (7–47%) in buffer A were prepared using a gradient mixer (Biocomp, Fredericton, N.B. Canada). The cytoplasmic fraction (20 *A*_260_) was applied onto the linear sucrose gradient (7–47%) and subsequently centrifuged at 260,000× *g* (Beckman SW41 rotor) for 150 min. Twenty 600-µl fractions were collected using an Isco density gradient fractionator (Teledyne Isco, Inc, Lincoln, NE) equipped with a 5-mm path length density gradient flow cell and a UA-6 UV/vis detector with built-in chart recorder.

### GRTH Co-immunoprecipitation

Polysome prepared from WT mice testis extracts were initially subjected to pre-clearing by incubation with 40 µl of protein A agarose (50% of slurry) and 2 µg of normal rabbit or mouse immunoglobulin G (lgG) in the immunoprecipitation assay buffer with gentle agitation. The recovered supernatant was incubated with polyclonal GRTH antibody (2 µg) raised in rabbits against GRTH peptide (aa. 465–477) and further purified by peptide affinity chromatography utilizing GRTH peptide coupled to CNBr activated Sepharose 4B [12], [14] or rabbit IgG (as control) for 2 h at 4°C in the presence of 1× protease inhibitor mixture (Roche Applied Sciences) to co-immunoprecipitate the GRTH–RNP complex. 50 µl of protein A-agarose in 50% slurry was added, and the incubation was continued for overnight at 4°C. Protein A-precipitated GRTH–RNP complex was recovered by brief centrifugation followed by three-times washes with assay buffer. The supernatant of IgG group was recovered as the control containing all the RNA species.

### RNA preparation and Microarray analysis

Total RNA was extracted from testicular polysome, GRTH/IgG immunoprecipitated polysome and purified testicular cells samples using TRIZOL reagent (Invitrogen, Carlsbad, CA) followed by micro RNA kit according to the manufacture's protocol. Target preparation, labeling, hybridization of RNA to mouse genome (Affymetrix 430-2.0) and scanning were performed using the Affymetrix protocol. Three chips were used for each experimental design. Briefly 1 µg of total RNA was reverse transcribed by using either one step (polysome or germ cells) or two steps (GRTH/IgG immunoprecipitated samples) target labeling procedure followed by fragmentation of the cRNA and hybridization to the chips. A confocal scanner was used to collect the fluorescence signals and the average signal from two sequential scans was calculated for each microarray. Affymetrix GCOS software was initially used to analyze and quantify the hybridized array. GenespringGX 11.5 software was used to quantify expression levels for targeted genes. Each analysis was based on triplicate samples. Lists of differentially expressed transcripts were generated statistically by using unpaired t-test corrected by Benjamini-Hochberg method with a P value cutoff of ≤0.05. A filtering criteria was applied to the analysis, including genes raw signal value of larger than 100, presence of the signal in all replica and fold change at least larger than 2. Internal control of RNA sample hybridization quality was monitored in each array according to manufacture protocol.

### Functional Network Analysis

IPA (version 9.0, www.ingenuity.com) was used to construct network predictions on all significantly expressed genes with at least 2-fold change (p<0.05). Gene symbol was used as the identifier and Ingenuity knowledge gene database was used as a reference for the pathway analysis. Top score ranked networks with p<0.01 were chosen to report in the study.

### RT-PCR and Real-time PCR quantification of gene expression

Total RNA prepared from the testicular polysome was treated with DNase I to remove any possible co-purified genomic DNA. 1 µg of RNA was reverse transcribed using a SuperScript III First Strand Synthesis System (Invitrogen) containing a mixture of oligo(dT)20. The first-strand cDNA was used as a template in real-time PCR with SYBR Green Master Mix and an ABI 7500 sequence detection system (Applied Biosynthesis). The cycling program was set as follows: denature at 95°C for 10 min, followed by 45 cycles of 95°C for 15 s and 60°C for 1 min. Specific primers for gene of interests were designed accordingly. The specificity of the PCR products was verified by melting curve and agarose gel analyses. The results presented are from three individual experiments, in which each sample was assayed in triplicate, normalized to the level of β-actin mRNA, and expressed as fold of wild type.

## Results

### Differential expression of testicular polysome associated genes in the wild type and GRTH knockout mice

**Figure 1:**
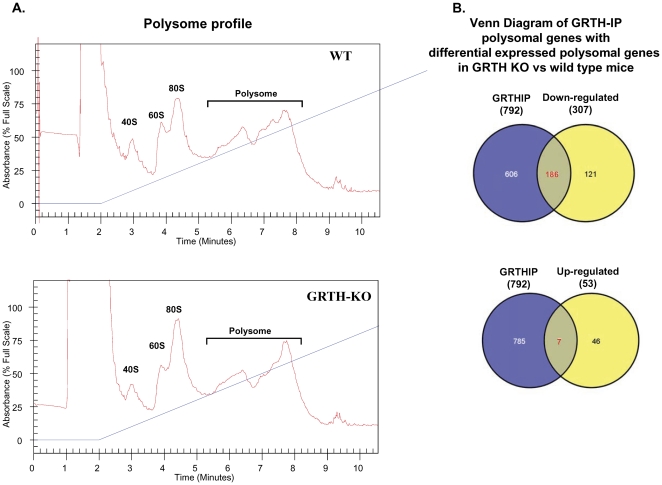
Differential expression of testicular polysome-associated genes in GRTH^−/−^ compared to WT mice. (**A**). A continuous sucrose-gradient (blue line, 7–47%) fractionation of wild type (WT) and GRTH^−/−^ (KO) mouse testis. Location of ribosomal subunits (40S and 60S), monoribosome (80S) and polyribosomes as indicated in the profile. RNA was extracted from the pooled of polysome fractions and used for microarray analysis. (**B**). Venn diagram of the overlap (red color) between total GRTH antibody immuno-precipitated (IP) polysomal mRNA messages (792) in WT mice testes and differentially expressed testicular polysomal genes (307 down and 53 up) in GRTH knockout compared to wild type mice. **Top panel**: down regulated genes. **Lower panel**: up regulated genes.

To examine the translational regulatory role of GRTH resulting from its association with RNA messages at polysomal sites, microarray studies were first conducted by comparing the differential mRNA expression pattern in the testicular polysomes of wild type and GRTH knockout (KO) mice. A typical polysome profile was observed in both WT and GRTH KO mice (Fig. 1 A). Using the RNA extracted from the pooled polysomal fractions as the probe for the microarray analysis of the entire mouse genome (Affymetrix 430.20), 307 genes were found to be significantly down-regulated (Table S1A) and 53 genes (Table S1B) up-regulated in the testicular polysomes of GRTH KO mice. To further identify the differentially expressed messages found in the GRTH null mice that might be associated with GRTH, initially study total GRTH-associated mRNAs in the testicular polysomes of wild type adult mice followed by Venn diagram analysis.

### Identification of polysome bound RNA as an integral component of GRTH-mRNP (messenger ribonuclear protein particles) complexes

To identify polysome-bound RNAs associated with GRTH, RNA extracted from immunoprecipitated (IP) testicular polysomes of WT with GRTH peptide antibody was used as the probe for microarray analysis. The specificity of the GRTH antibody (Ab) used in the present study has been previously demonstrated in the GRTH KO mice with completely absence of the GRTH protein [13] (negative control), and by an *in vitro* overexpressed GRTH protein [12] (positive control). In addition we have use the specific peptide as competitor inhibitor of the antibody-GRTH association. Success of immunoprecipitation (IP) using GRTH Ab is routinely validated by the presence of CRM1 since GRTH is known to interact with CRM1 involved in nuclear export pathway for mRNA transport from nuclear to the cytoplasm sites [14]. 792 genes (Table S2) were immunoprecipitated from total RNA messages associated with polyribosomes. IgG was used as control. 60.6% of testicular down-regulated (186 /307) and 13.2% (7/53) up-regulated genes derived from the differential analysis (see above, Fig. 1A and Table S1A) were associated in testicular polysomes as GRTH-RNA complexes in wild type mice. These were revealed by intersecting the mRNAs from total testis (307 down- and 53 up-genes) with GRTH-IP associated with polysomes (792 genes) by Venn diagram (Fig. 1B). Gene lists of GRTH protein associated with either 186 down- or 7 up- regulated genes in testicular polysomes of GRTH null mice are shown in Table S3 A & B.

**Figure 2:**
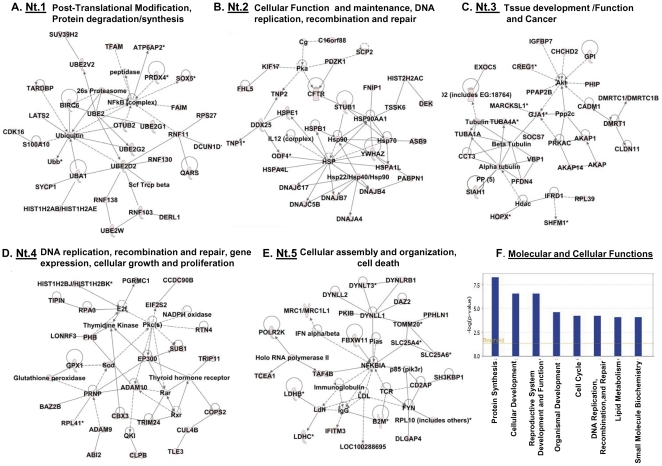
Network pathway analysis of mRNA messages associated with GRTH in testicular polysomes of WT mice. (**A–E**). Five different top score of the associated network pathway predicted by the ingenuity pathway analysis (IPA) are presented. The network was constructed by genes with shape representing functional class of the gene product (www.ingenuity.com). Genes in light pink color are the polysomal mRNA message immunoprecipitated by GRTH antibody. Uncolored genes are predicted by ingenuity pathway knowledge data base as the biological relevance to that network. Solid line: direct interaction. Dotted line: indirect interaction. (**F**). IPA predicts eight different top score (p<0.5) of molecular and cellular functions in the testicular polysomal mRNA messages associated with GRTH protein in adult mice.

**Figure 3:**
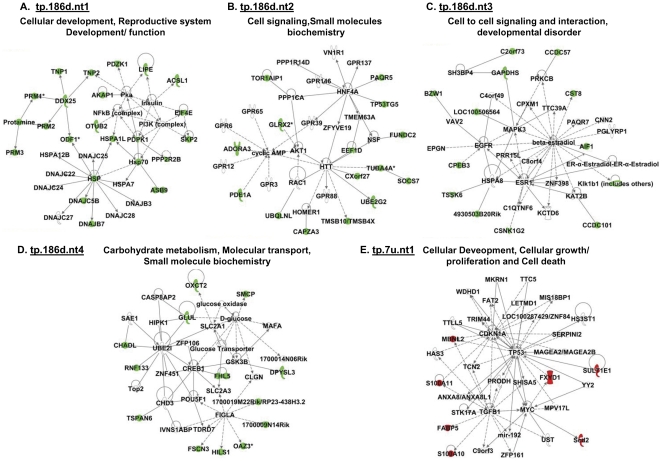
Network pathway analysis of differentially expressed testicular polysomal GRTH bound-mRNAs between GRTH^−/−^ and WT mice. IPA predicts four different top score of down (d)-regulated (**A–D**) networks and one up (u)-regulated (**E**) network in the genes differentially expressed between GRTH KO versus wild type testicular polysome. tp # (d/u) nt * - testicular polysomal mRNAs (t) immunoprecipitated by GRTH antibody (p) 186 (d) or 7 (u) network (nt). Genes in color green (down-regulated), red (up-regulated) and uncolored (biological relevance to the network with no change in expression between WT and GRTH^−/−^).

To learn the significance of the genes derived from the above microarray analysis in relation to GRTH regulation in the testis, we performed comprehensive bioinformatic analysis using Ingenuity pathway (IPA) software (www.Ingenuity.com) to assess integrated function and pathways of these polysomal GRTH bound mRNAs (792) (Fig. 2) and those GRTH-bound differentially expressed genes revealed in these studies (GRTH WT versus KO) (Fig. 3). An overview of the 792 GRTH-bound mRNAs noted in WT pointed to genes involved in protein synthesis, cellular development, reproductive system development and function among others (Fig. 2F). IPA further reveals five top score networks including genes highly relevant to post-translational modification, protein degradation/synthesis pathway and to cellular function, DNA replication, recombination, tissue development, cellular growth, proliferation and death (Fig. 2A–E). The first network (Fig. 2A) with centered nuclear factor kappa B (NFκB) complex connects to the network of ubiquitin/ubiquitin conjugating enzymes (UBE2:G1, G2, D2, V2, W). This further links to proteasome and ring finger proteins (RNF: 11, 103, 130, 138). Ubiquitin-like modifier activating enzyme 1 (UBA1) is also shown to interact with meiotic synaptonemal complex protein 1 (SYCP1) and histone cluster 1 H2AB/H2AE (Hist1H2AB/HIST1H2AE) (Fig. 2A). The second network (Fig. 2B) includes the heat shock protein complex (HSP) with members, Hsp90 (Hsap90AA1), Hsp70 and Hsp70 like (Hspa1l, Hspa4l), members of Hsp40 family (DNAJB7, DNAJC5B, DNAJA4, DNAJC17, DNAJB4), Hsp27 (HspB1) and Hsp10 (HspE1). Histone cluster 2 H2AC (HIST2H2AC) links to Hsp90 via testis-specific serine kinase 6 (TSSK6). It also interacts with transcriptional regulator, DEK oncogene (DEK). Ddx25 (GRTH) and the germ cells specific messages including outer dense fiber of sperm tails (ODF) and chromatin remodeling transition protein 1 and 2 (TP1/2) are also present in this network. Network 3 includes Akt as centered gene signaling, cAMP dependent protein kinase A (PRKAC) complex and members of A kinase anchor proteins (AKAP). Tubulin associated network with biological function in apoptosis, cell cycle and germ cell-Sertoli cell junction signaling is part of this network (Fig. 2C). Network 4 includes transcriptional coactivator (EP300) and protein kinase C and Prion protein (PRNP) centered pathway (Fig. 2D). Network 5 includes nuclear factor kappa BIA (NFκBIA) (a member of I-κ B proteins which inactives NFκB complex formation) and Dynein (Dnyll1, Dynll2, DnynRB1, DynlT3) centered complexes. DAZ2 restricted to premeiotic spermatogonia is also associated with Dynein associated pathway.

Regarding to the networks associated with differentially expressed GRTH bound genes in testicular polysome observed in the GRTH null mice, four top score networks of down regulated genes (tp.186d.nt1–4) (Fig. 3A–D) and one network of up regulated genes (tp.7u.nt1) (Fig. 3E) were predicted by IPA. The first down-regulated network (tp.186d.nt1) consists of 35 nodes and included 23 down-regulated expressed genes. HSP, NFκB and DDx25 are the three centered complex pathways (Fig. 3A). Network 2 (tp.186d.nt2) consists of factors acting on Huntington's disease signaling (HTT), HNF4A and cAMP signaling (Fig. 3B). Network 3 (tp.186d.nt3) consists of 13 down-regulated genes of total 33 nodes indirectly associated with estrogen action (ESR1) (Fig. 3C). Network 4 (tp186d.nt4) includes ubiquitin, (UBE21)/cyclic AMP response element binding protein (CREB1) and transcriptional factor specific for folliculogenesis helix loop protein (FIGLA) (Fig. 3D). All 7 of up-regulated GRTH bound polysomal messages (tp.7u.nt1) participate TP53-, Myc- and transforming growth factor TGFB1 centered pathway (Fig. 3E). The predicted network information of those non-GRTH associated differentially expressed genes (46 up and 121 down, Fig. 1C) is provided in Fig. S1.

### Identification of differentially expressed GRTH associated mRNAs in the polysome of purified testicular cells

**Figure 4:**
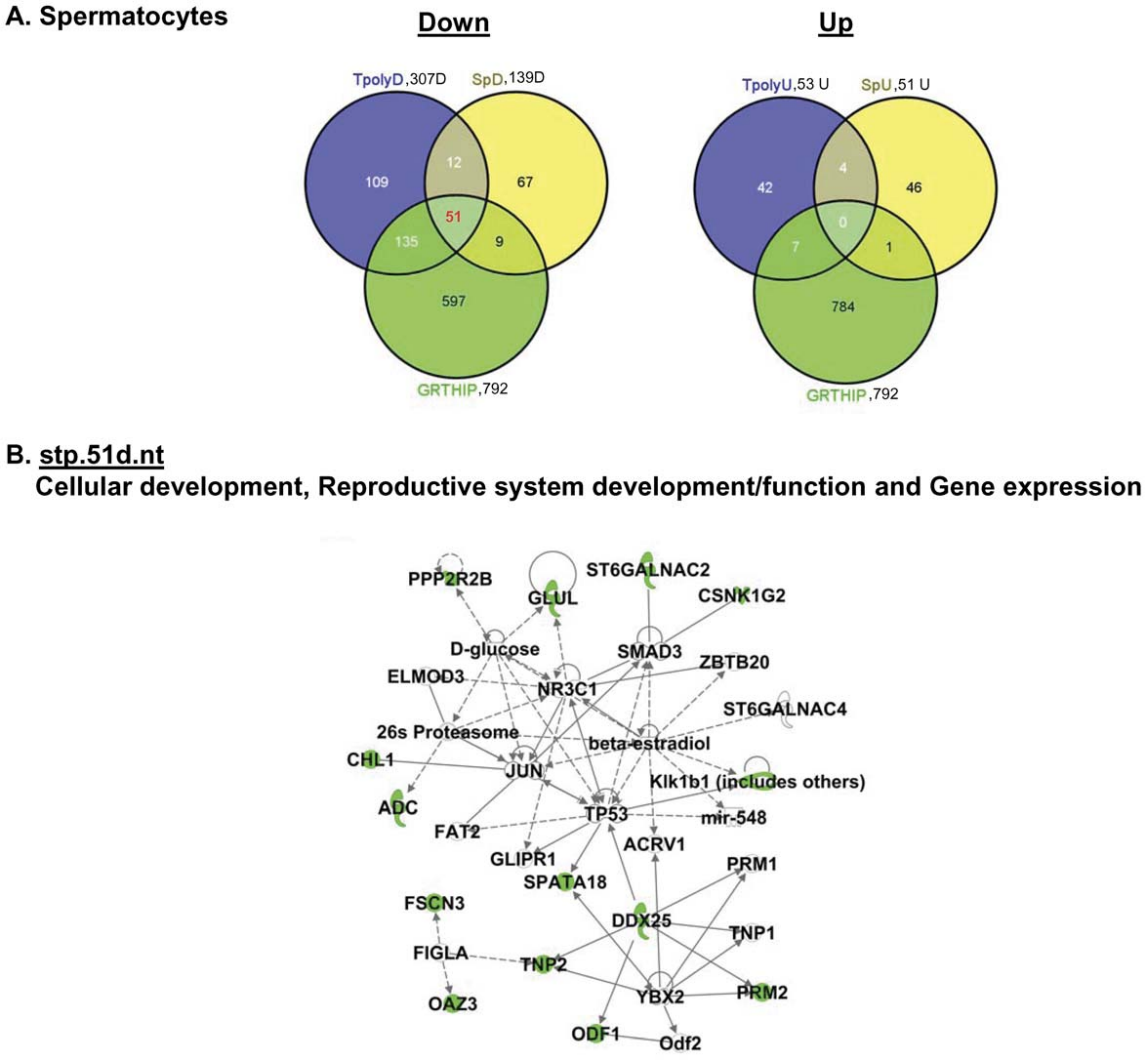
IPA analysis of differentially expressed genes in spermatocytes of GRTH^−/−^ compared to WT mice. (**A**). Venn diagram analysis of the overlap among differentially regulated genes in spermatocytes (**left panel**,139 down or **right panel**, 51 up-regulated genes), in testicular polysomes (**left panel**, 307 down - or **right panel**, 53 up-regulated genes) and polysomal GRTH IP genes noted in WT mice (792 genes). Tpoly3fd or Tpoly3fu: >3 fold (f) down (d) or up (u)- regulated genes found in testicular polysomes (Tpoly); sp2fd or sp2fu: >2 fold down- or up-regulated genes found in spermatocytes (sp); GRTHIP: mRNA messages were immunoprecipitated (IP) by GRTH antibody in testicular polysomal fraction of wild type mouse testis. (**B**). IPA predicts one top score of network (nt) function in 51 down regulated genes (A, in red) associated with GRTH protein in polysomes of spermatocytes. stp: spermatocytes (s), testicular polysome (t) and GRTH IP (p). Genes in color green (down-regulated) and uncolored (relevant biological genes to the network with no change in expression between WT and GRTH^−/−^).

**Figure 5:**
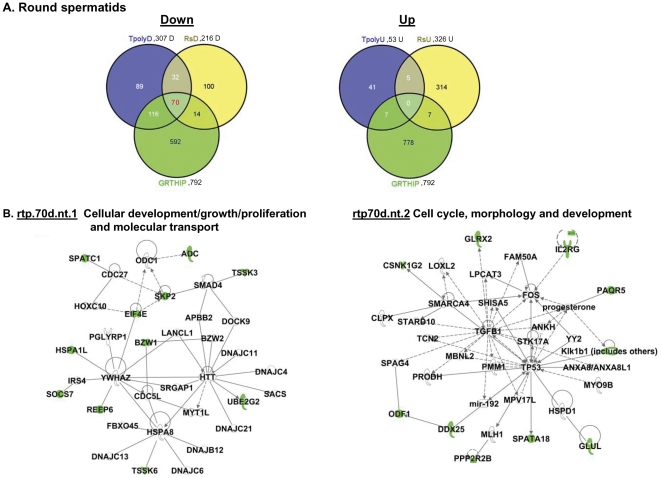
IPA analysis of differentially expressed genes in round spermatids of GRTH^−/−^ compared to WT mice. (**A**). Venn diagram analysis of the overlap among differentially regulated genes in round spermatids −216 down (**left panel**)- and 326 up (**right panel**) -regulated genes , in testicular polysomes (307 down - or 53 up-regulated genes) and polysomal GRTH IP genes noted in WT mice (792 genes). Tpoly3fd or Tpoly3fu: >3 fold (f) down (d) or up (u)- regulated genes found in testicular polysomes (tpoly); rs3fd or rs3fu: >3 fold down- or up-regulated genes found in round spermatids (rs); GRTHIP: mRNA messages were immunoprecipitated (IP) by GRTH antibody in testicular polysomal fraction of wild type mouse testis. (**B**). IPA predicts two top score of network (nt) function in 70 down- regulated genes (A, in red) associated with GRTH protein in polysomes of round spermatids. rtp: round spermatids (r), testicular polysome (t) and GRTH IP (p). Genes in color green (down-regulated) and uncolored (relevant biological genes to the network with no change in expression between GRTH^−/−^ and WT).

**Figure 6:**
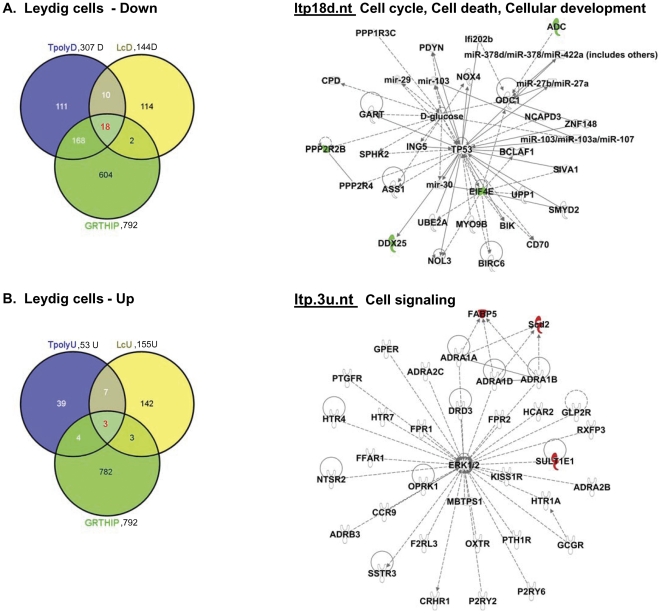
IPA analysis of differentially expressed genes in Leydig cells of GRTH^−/−^ compared to WT mice. **(A) and (B). Left panel-**Venn diagram analysis of the overlap among differentially regulated genes in Leydig cells (144 down - and 155 up-regulated genes), in total testicular polysomes (307 down - or 53 up-regulated genes) and polysomal GRTH IP genes noted in WT mice (792 genes). Tpoly3fd or Tpoly3fu: >3 fold (f) down (d) or up (u)- regulated genes found in testicular polysomes (tpoly); lc3fd or lc3fu: >3 fold down- or up-regulated genes found in Leydig cells (lc); GRTHIP: mRNA messages were immunoprecipitated (IP) by GRTH antibody in testicular polysomal fraction of wild type mouse testis. **Right panel.** IPA predicts one network (nt) function in (**A**) 18 down- and (**B**) 3 up- regulated genes (A, in red) associated with GRTH protein in polysomes of Leydig cells. ltp: Leydig cells (l), testicular polysomes (t) and GRTH IP (p). Genes in color green (down-regulated), red (up-regulated) and uncolored (relevant biological genes to the network with no change in expression between WT and GRTH^−/−^).

Because the above studies in isolated polysomes were not feasible to be performed in purified testicular cell populations due to the limited sample size to attain their purification, a different approach was followed for this part of the study. First, RNA extracts from purified testicular cells of WT and GRTH KO mice including Leydig and germ cells (spermatocytes and round spermatids) were subjected to microarray analysis. These studies revealed that 139 genes down-regulated and 51 genes up-regulated in spermatocytes (Table S4A & 4B), 216 genes down–regulated and 326 genes up-regulated in round spermatids (Table S5A & 5B) and 144 mRNAs down-regulated and 155 up-regulated in Leydig cells of KO versus WT (Table S6A & 6B). Subsequently Venn-diagram was used to detect transcripts that were regulated by GRTH as polysomal GRTH-bound messages (indicated in the center space of the three circles) by intersecting GRTH regulated genes of particular testicular cell type with differentially expressed down- and up- regulated genes in total testicular polysomes (Fig. 1A, 307 down- or 53 up-regulated genes) and GRTH IP polysomal samples (792 genes, Table S2) to draw conclusions. 85% ((51/51+9), 83.5% (70/70+14) and 90% (18/18+2) of GRTH IP transcripts from polysomes (intercept) of spermatocytes (Fig. 4A, left panel), round spermatids (Fig. 5A, left panel) and Leydig cells (Fig. 6A, top panel), respectively were found in testicular polysomes. Less than 7% of down-regulated GRTH IP genes were not present in the total testicular polysome which validate this analytic approach [Spermatocytes: 9/135+51+9 (Fig. 4A, left panel), Round spermatids: 14/70+116+14 (Fig. 5A, left panel), LC: 2/168+18+2 (Fig. 6A, top panel)]. GRTH regulated genes localized at cytoplasmic sites other than polysomes were outside the region intersecting the specific cell type with total polysome of both down regulated - and GRTH IP- transcripts (Fig. 4A, Spermatocytes: 67 genes, Fig. 5A, Round spermatids, 100 genes; Fig. 6A, Leydig cells: 114 genes). Very few up-regulated genes were found as GRTH associated in polysomes of Leydig cells only (3 genes) (Fig. 6B, left panel) but none in either spermatocytes or round spermatids (Fig. 4A & 5A, right panel). The complete gene lists of GRTH-associated messages in polysomes of individual testicular cells are shown in Table S7.

Within 51 down-regulated GRTH IP genes in polysomes of spermatocytes, IPA predicted a network highly involved in cellular development, reproductive development and function (stp.51d.nt). This includes Ddx25 and TP53 centered interactions (Fig. 4B). Two top score networks are noted in round spermatids: Network 1 includes possible 14-3-3 mediated signaling (YWHAZ) pathway (rtp70d.nt.1) and network 2 (rtp70d.nt.2) associates with TGFB1 and TP53 signaling. Both link to cellular development/growth/proliferation and cell cycle (Fig. 5B). In Leydig cells, one potential network of those 18 down-regulated genes predicted by IPA (ltp18d.nt) is TP53 centered pathway including Ddx25, EIF4E and ADC (Fig. 6A, right panel). 3 up-regulated genes, fatty acid binding protein (FABP5), stearoyl-CoA-desaturase (Scd2) and sulfotransferase family 1E (Sult1E1) participate in fatty acid biosynthetic process, lipid metabolism and steroid metabolism (Fig. 6B, right panel, ltp.3u.nt). The overall networks for the differentially expressed genes in each cell types predicted by IPA are shown in supplemental figures: spermatocytes (Fig. S2), round spermatids (Fig. S3) and Leydig cells (Fig. S4).

### Validation of differential gene expression in polysomes of mouse testis

**Figure 7:**
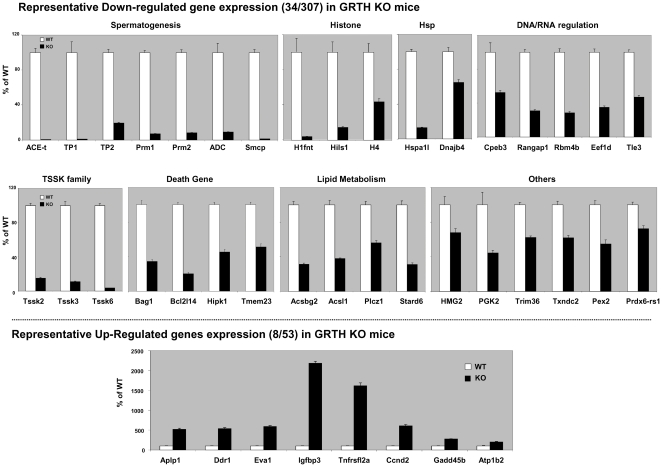
qRT-PCR validation of representative differentially expressed genes in testicular polysomes of GRTH^−/−^ versus WT mice. Expression of a panel of candidate genes in **GRTH^−/−^** (KO) and wild type (WT) was chosen for further validation by RT-PCR. Gene expression level from three independent experiments were quantified and normalized by β-actin (means ± se). The KO values are presented as percentages of WT. The overall trends of expression by real time PCR were in agreement with the array data. **Top and middle panels:** down-regulated genes. **Bottom panel:** up-regulated genes.

**Figure 8:**
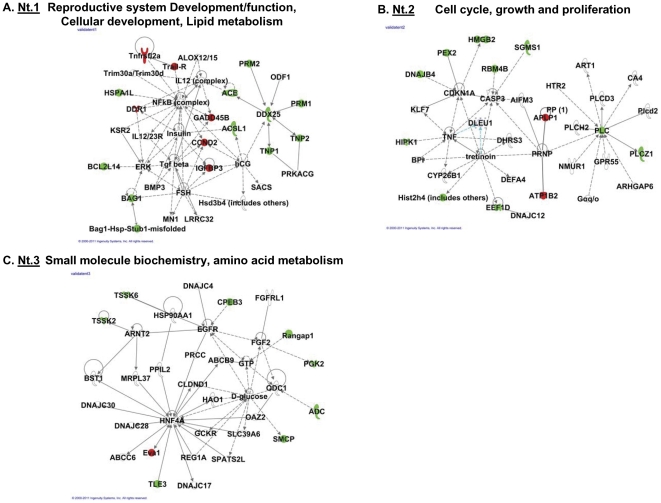
Network function of validated differentially expressed genes in testicular polysomes of GRTH^−/−^ versus WT mice. Three top score of the associated network pathways from validated genes (Fig. 7) were presented by ingenuity pathway analysis (IPA). Genes in color green (down-regulated), red (up-regulated) and uncolored (relevant biological genes to the network with no change in expression between WT and GRTH KO).

The validation criteria is based on GRTH known functions as a male specific gene and essential during spermatogenesis including chromatin remodeling process, spermatid elongation, RNA helicase functions, apoptotic effect, transport, and its role in steroidogenesis. In the case of up- regulated genes, we randomly selected 8 out of 53 genes since very little is known about them at the present time. The expression of selected individual genes within the polysomal fractions (WT and KO) was determined by real time RT-PCR. These for the most part were in good agreement with the array data and only in few cases the fold-change did not match. Eight categories of genes are presented based on the biological and molecular functions (Fig. 7). Background of individual genes is shown in Table 1 [13]–[15], [30]–[67]. Decreases in the protein expression level such as ACE, Tp1/2 and protamine 1 and 2 in GRTH knockout mice have been validated (not shown). The IPA predicted three network functions from these validated differentially expressed genes in testicular polysomes (Fig. 8). Each network consists of 35 nodes. Top score network (Fig. 8A) presents 11 out of 34 down-regulated genes and 6 out of 8 up-regulated genes. NFκB, TGF β and DDX25 are the three center interacting genes of the signaling network. The 2^nd^ network (Fig. 8B) consists of 9 down- and 2 up- of the validated differentially expressed 42 genes. It associates with Tumor necrosis factor (TNF), Caspase 3 (Casp3) and phospholipase C (PLC) and link to cellular apoptotic signaling. The 3^rd^ network (Fig. 8C) consists of 8, down- and one (Eva1) up- out of the validated genes and directly or indirectly link to HNF4A for TGF-β signaling pathway.

**Table 1:** Background of the validated differentially expressed testicular polysomal genes in GRTH^−/−^ compared to WT mice.

	Gene	Microarray data	Gene Description	Function
DDX family	DDX25	polysome, IP, sp,rs,lc	Gonadotropin regulated testicular RNA helicase	Multifunctional RNA helicase: translation, RNA transport, CB structure integrity, steroid synthesis [13], [14], [15].

Down regulated gene in GRTH KO mice verified by RT-PCR				
Spermatogenesis	tACE	polysome	Testis-specific angiotensin-converting enzyme	Transporting of tACE from nucleus (N) to cytoplasm (C) is reduced in the absence of GRTH [13], [14].
	TP1	polysome, IP	Transition protein 1	Chromatin remodeling [30].
	TP2	polysome, IP, sp	Transition protein 2	Chromatin remodeling, mRNA translocation efficiency from N to C is regulated by GRTH [13], [14].
	Prm1	polysome, IP	Protamine 1	Chromatin remodeling [30].
	Prm2	polysome, IP, sp	protamine 2	Chromatin remodeling [30].
	ADC	polysome, IP, sp,rs,lc	arginine decarboxylase	Ornithine metabolic/polyamine biosynthetic process [31].
	Smcp	polysome, IP	Sperm mitochondria-associated cysteine-rich protein	Binding of spem to zona pellucida, sperm motility [32], [33].
	HMG2	polysome	High mobility group 2 protein	DNA binding/bending activity, protein but not mRNA expression is regulated by GRTH [13], [14].
	PGK2	polysome	Mus musculus phosphoglycerate kinase 2	Transporting of PGK2 from nucleus to cytoplasm is reduced in the absence of GRTH [13], [14].
	Trim36	polysome	Tripartite motif protein 36	Acrosome reaction [34].
	Txndc2	polysome, IP	Mus musculus thioredoxin domain containing 2	Spermatogenesis (spermatozoa)/ thiol-disulfide exchange intermediate activity [35].
Histone	H1fnt	polysome, IP	H1 histone family, member N, testis-specific	Sperm nuclear elongation/H1fnt exclusively in haploid spermatids [36].
	Hils1	polysome, IP	Histone H1-like protein in spermatids 1	Sperm nuclear elongation/localized to the nuclei of round and elongating spermatids [37].
	H4	polysome, IP	Histone 4	One of core histone for nucleosome assembly, protein not RNA expression is regulated by GRTH [13], [14].
Heat schok protein	Hspa1l	polysome, IP, rs	Heat shock protein 1-like	ATP binding, expressed in elongating and elongated spermatids [38].
	Dnajb4	polysome, IP	DnaJ (Hsp40) homolog, subfamily B, member 4	Protein folding [39].
DNA/RNA regulation	Cpeb3	polysome, IP	Cytoplasmic polyadenylation element binding protein 3	CPEB3 is a sequence-specific translational regulatory protein [40].
	Rangap1	polysome, IP	RAN GTPase activating protein 1	Signal transduction/GTPase activator activity [41], [42].
	Rbm4b	polysome	RNA binding motif protein 4B	mRNA processing [43].
	Eef1d	polysome, IP	Eukaryotic translation elongation factor 1 delta	Translational elongation [44].
	Tle3	polysome, IP, rs	Transducin-like enhancer of split 3, homolog of Drosophila E(spl)	Spermatogenesis/transcriptional regulation [45].
TSSK family	Tssk2	polysome	Testis-specific serine kinase 2	Spermatogenesis/phosphorylation serine/threonine kinase activity [46], [47].
	Tssk3	polysome, IP, sp,rs,lc	Testis-specific serine kinase 3	Spermatogenesis/phosphorylation serine/threonine kinase activity, expressed in Leydig cell [46], [47].
	Tssk6	polysome, IP, sp,rs	Testis-specific serine kinase 6	Sperm chromatin condensation/localized to heads of elongating spermatids [46], [47].
Death Gene	Bag1	polysome	Bcl2-associated athanogene 1	Hsp70-binding protein that can collaborate with Bcl-2 in suppressing apoptosis [48].
	Bcl2l14	polysome	Bcl2-like 14 (apoptosis facilitator)	BCL-G/BCL2L14 gene and that induction of this gene contributes to p53-mediated apoptosis [49].
	Hipk1	polysome,rs	Homeodomain interacting protein kinase 1	DNA damage response/signal transduction by p53 class/nuclear serine/threonine kinases [50].
	Tmem23	polysome	Transmembrane protein 23	Sphingomyelin synthase 1 [51], [52].
Lipid metabolism	Acsl1	polysome, IP	Acyl-CoA synthetase long-chain family member 1	Lipid metabolic process/fatty acid degradation [53].
	Acsbg2	polysome, IP	Acyl-CoA synthetase bubblegum family member 2	Lipid metabolic process [54].
	Plcz1	polysome	Phospholipase C, zeta 1	Lipid metabolic process/phospholipase C activity [55], [56].
	Stard	polysome	StAR-related lipid transfer (START) domain containing	Steroid biosynthetic process/cholesterol binding/lipid transport [57].
Others	Pex2	polysome	Peroxin 2	Peroxisome organization/biogenesis [58].
	Prdx6-rs1	polysome	Peroxiredoxin 6, related sequence 1	Lipid catabolic process/antioxidant activity [59].

Up regulated genes in GRTH KO mice verified by RT-PCR				
	Gene	Microarray data	Gene Description	Function
	Ddr1	polysome	Discoidin domain receptor family, member 1	Cell adhesion/protein-tyrosine kinase activity [60].
	Eva 1	polysome	Epithelial V-like antigen 1	Possible role in placenta morphogenesis [61].
	Aplp1	polysome	Amyloid beta (A4) precursor-like protein 1	mRNA plyadenylation/cell adhesion/apoptosis [62].
	Igfbp3	polysome	Insulin-like growth factor binding protein 3	Positive regulation of apoptosis [63].
	Tnfrsf12a	polysome	Tumor necrosis factor receptor superfamily	Poptosis/cell adhesion/angiogenesis [64].
	Ccnd2	polysome	Cyclin D2	Regulation of cell cycle/ proliferation/ protein kinase activity [65].
	Gadd45b	Polysome, rs,lc	Growth arrest and DNA-damage-inducible 45 beta	Activation of MAPKKactivity/cell cycle [66].
	Atp1b2	polysome	ATPase, Na+/K+ transporting, beta 2	Cell ladhesion/ion transport/ATPase activity [67].

IP: immunoprecipitation by GRTH antibody., SP: spermatocytes., RS: round spermatids., LC: Leydig cells. IP: Testicular polysomal mRNA immunoprecipitated by GRTH antibody.

## Discussion

Global analysis of gene expression in GRTH KO compared to wild-type mouse testis by microarrays revealed cell specific targeted gene regulation by GRTH. More than 50% mRNAs of down-regulated genes were associated with GRTH in polysomes. Unlike the down-regulated genes, the majority of up-regulated genes (>87%) were free transcripts (non-polysome bound). Most of up-regulated genes were found in round spermatids.

To expand the repertoire of genes connected with the various functions of GRTH in the testis, we first applied molecular network modeling to evaluate the entire set of pathways modulated by GRTH bound transcripts in testicular polysomes. The ubiquitylation-proteasome network pathway ranked the highest score among GRTH-bound gene transcripts in testicular polysomes (Fig. 2A). Ubiquitin, ubiquitin-like proteins, different type of E2-like conjugating enzymes, 26 s proteasome and ring finger proteins, known to interact with ubiquitin/E2D2, are present in this pathway. Since ubiquitin dependent post-translational modifications are essential in male germ cells development and quality control [19], a GRTH-linked ubiquitin-proteasomal process could be envisioned as a critical step to regulate gene expression during spermatogenesis. IPA also suggests the importance of a group of functionally related heat shock proteins (HSP) that could act as chaperons or be involved in the folding/unfolding of GRTH-targeting proteins relevant for germ cells development (Fig. 2B). The binding partner of HSP90, testicular specific serine/threonine kinase (TSSK6) and also TSSK6 phosphorylated histone (H2A) are present in the HSP network system. TSSK6 is essential for male fertility for its impact on chromatin remodeling in elongating spermatid [20]. TSSK6 is decreased in the testicular polysome of GRTH null mice (Fig. 7) which are sterile with spermatids that fail to elongate [13]. This indicates the importance of TSSK6-HSP pathway via GRTH regulation in male reproduction. The relevance of GRTH-HSP action is further supported by the evidence that the majority of these HSP signaling molecules were down-regulated in the absence of GRTH (Table S1A). These molecules were also grouped in the highest score IPA predicted network signaling with HSP at its center in the pathway (Fig. 3A, tp.186d.nt1). GRTH associated mRNA messages of histone cluster 1, H2AB/H2AE also link to ubiquitin-like modifier activating enzyme 1 (UBA1) and further extend to the ubiquin network signaling (Fig. 1A, Nt.1). Because of the essential role of histone ubiquitination in the chromatin remodeling to permit transition proteins /protamines replacement leading to final mature germ cell production [21], [22], [23], this GRTH-histone-ubiquitin network offers an additional regulatory route of GRTH action to be explored during spermatogenesis.

**Figure 9:**
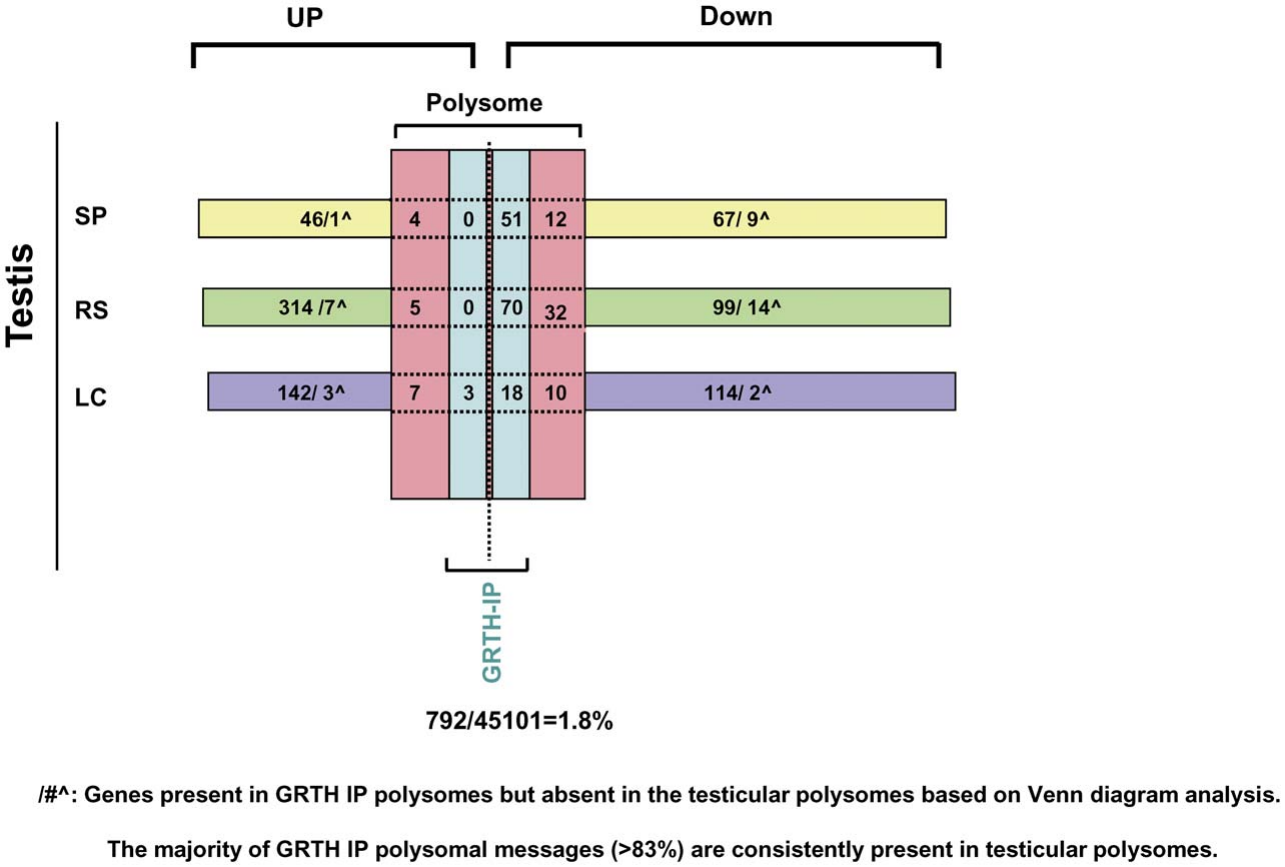
Cellular distribution profile of differentially regulated GRTH-bound polysomal genes in mouse testicular cells. Number of differentially regulated genes not associated with polysomes- spermatocytes (SP) in yellow, spermatids (RS) in green and Leydig cells (LC) in purple. Number of transcripts found in polysome but not associated with GRTH protein (column in pink). Number of transcripts associated with GRTH protein in polysomes (column in light blue). /#∧: few genes present in GRTH-IP polysomes but absent in the testicular polysomes revealed by Venn diagram analysis (Fig. 4, 5, 6).

Profiles of differentially regulated genes as GRTH-mRNA complexes in the polysomes of individual testicular cells (Fig. 9) were predicted by Venn diagram. Candidate genes were identified by overlapping the total polysomal GRTH-bound genes with differentially down-regulated genes present in both testicular polysomes and total cellular extracts of purified testicular cells (spermatocytes, round spermatids or Leydig cells). The three way- rather than two way- overlapping the gene lists of GRTH- IP with those of total cellular extracts exclude potential analytic error in the prediction. From the entire mouse genome, 1.8% of GRTH- bound genes were associated with polysomes. 51 genes in spermatocytes, 70 genes in round spermatids and 18 genes in Leydig cells were down-regulated in GRTH KO mice (Fig. 9 and Table S7). Within this population, 35 genes are commonly present in both spermatocytes and round spermatids and 12 out of these genes are also present in Leydig cells (Table S7). IPA predicted overall agreement in their network function associated with cellular development and reproductive system (Fig. 4C, 5C and 6B) which is consistent with the earlier defined critical role of GRTH function in germ cell development [13], [24].

The present IPA analysis also shows the active participation of NFκB complex network in GRTH mediated action. This network (Fig. 2A) linked to GRTH associated transcripts either directly to proteasome or indirectly to the molecules such as Fas apoptotic inhibitory molecule (FAIM), ring finger protein 11, ubiquitin E2 (UBE2), ubiquitin specific protease (OTUB2), ubiquitin, mitochondrial transcriptional factor A (TFAM), antioxidant peroxiredoxin 4 (Prdx4), gene encoding ATPase associated with energy conservation/transport (ATP6AP2) and sex determining region Y box 5 (SOX5) involved in Wnt-β-catenin signaling. In the absence of GRTH, the NFκB complex is also presented as the center in the pathway connected to polysomal down-regulated genes heat shock protein HSPA1L, OTUB2, cell proliferation regulator 3-phosphoinostide dependent protein kinase 1 (PDK1) and protein kinase A (Fig. 3A, tp186d.nt1). In an early study, we found that GRTH promotes the NFκB mediated anti-apoptotic pathway through controlling the transfer of NFκB dimers from cytoplasm to the nucleus. This consequently stimulates the transcription of anti-apoptotic genes including Bcl-2 and Bcl-xL [24]. Since NFκB signaling plays a crucial role in the proliferation, survival and differentiation of the cells, the predicted NFκB network provides a potential mechanistic angle to GRTH mediated action through controlling expression of these genes and further impacting on NFκB signaling to control apoptosis in testicular germ cells of adult mice [24].

It is also of interest the finding of the tumor suppressor protein TP53/transforming growth factor β1 (TGFB1) network as the major pathway not only in the up-regulated GRTH associated messages extracted from total testicular polysomes (Fig. 3E, tp.7u.nt1) but also in down-regulated messages from individual testicular cells: spermatocytes (Fig. 4B, stp.51d.nt), round spermatids (Fig. 5B, rtp70d.nt) and Leydig cells (Fig. 6A-right panel, ltp18d.nt). TP53 responds to various cellular stresses to regulate the cell cycle and genomic stability, whereas TGFB1, a member of TGFβ superfamily of cytokines, positively or negatively regulates cell fate pending on interacting molecules and particular cellular context. Expression of TP53 [25], [26] and TGFB1 [27], [28] is developmental dependent in the testis. TP53 is known to play a role in spermatogonial differentiation [26] and meiotic progress of spermatocytes [25]. On the other hand, TGFB1 is known to participate in Sertoli/germ cell interaction and the onset of spermatogenesis [29]. Although GRTH does not apparently alter the mRNA level of either gene, TP53 protein was found significantly increased in the spermatocytes of GRTH null mice [24]. The evidence of several differentially regulated genes regardless the testicular cell type converging to a common main network of TP53/TGFB1 pathway (Fig 4, 5, 6) underscores the important regulatory role of GRTH-TP53-TGFB1 linkage in male reproduction and fertility control.

To further gain insights into the GRTH regulatory action in polysomes, the differential profile of a panel of genes with diversified biological/cellular functions was confirmed by reverse-transcription and real time PCR analysis (Fig. 7). This study revealed an undiscovered panel of GRTH regulated genes shown in Table 1. The differential profile of a panel of genes with diversified biological/cellular functions was confirmed by reverse-transcription and real time PCR analysis (Fig. 7). Among these genes, similar change at both cellular and polysomal level of GRTH KO mice was observed only in ADC, and TSSK3 which are commonly down-regulated in all three types of testicular cells [spermatocytes (SP), round spermatids (RS) and Leydig cells (LC)]. In other cases, we observed Tp2/Prm2 down-regulated in SP only, TLE3/Hspa1l/Hipk1 down-regulated in RS only and Gadd45b up-regulated in both RS and LC. The significance of these cell specific GRTH regulated gene expressions in the testis requires further investigation. In GRTH KO some of these genes found decreased at testicular polysomal sites were either not altered (H4/ PGK2) or minimally decreased (HMG2/tACE) at the total cellular mRNA level with abolished protein expression [13], [14] (Table 2). Because of the finding of marked decrease of tACE and PGK2 in the cytoplasm by Northern analysis and of cytoplasm/total ratio in those studies, we concluded that GRTH through binding mRNAs is required for the export (via the CRM1 pathway) of selected RNA species from nuclear to the cytoplasmic sites. Thus, it is likely that comparable mRNA expression at the total cellular level between GRTH KO and wild type with clear reduction at polysomal level in the absence of GRTH might result from decreased RNA export efficiency and/or the inability to be transported to polysomes and their impaired association at these sites. The general decrease of RNA messages in polysomes without a change in total cellular level would impact protein translation where GRTH may act as a translational regulator during germ cell development.

**Table 2:** Summary of relevant spermatogenic gene expression from microarray analysis and early studies in GRTH knockout mice compared to the wild type.

Gene	WT		KO	versus	WT	
	IP		RNA			Protein
	Microarry	Microarray	Northern			Western
	GRTH-mRNA	Polysome	Total	Cyto.	Cyto./Total	Total
H4	Yes	D	NC	NC	NC	Abolish
HMG2	No	D	D*	D*	NC	Abolish
PGK2	No	D	NC	D	D	Abolish
Acr	No	D	NC	NC	NC	NC
tACE	No	D	D*	D	D	Abolish
TP1	Yes	D	NC#	NA	NA	Abolish
TP2	Yes	D	D	D	D	Abolish
Prm1	Yes	D	NC#	NA	NA	Abolish
Prm2	Yes	D	NC#	NA	NA	Abolish

IP: Testicular polysomal mRNA immunoprecipitated by GRTH antibody.

KO: GRTH knockout mice., WT: wild type. D: decrease., D*: minor decrease.

NC: no change., NC#: no change detected by qRT-PCR [13]., NA: not available.

Microarray data are derived from the present study.

Northern and Western data are derived from early study [14].

Binding of GRTH to RNA [13] might be highly selective during germ cells development. We have earlier demonstrated GRTH bound to a selective panel of cellular pro- and anti-apoptotic mRNAs [24] and also genes essential for germ cell development [13]. GRTH does not appear to be associated with any message involved in the RNA interference silencing complex [15]. When we analyzed those 34 down- and 8 up-regulated genes ( Fig. 7), 21 down-regulated messages out of these total 42 gene (Ddx25, TP1/2, Prm1/2, ADC, Smcp, Txndc2, H1fnt, Hils1, H4, Hspa1l, Dnajb4, Cpeb3, Rangap1, Edf1d, Tle3, Tssk3, Tssk6, Ascl1 and Acsbg2) (Table 1) were found as GRTH immuno-precipitated polysomal messages. It is interesting to note that Cpeb3, Rangap1 and Eef1d are involved in RNA regulation as translational regulator. This observation further depicts the selectivity of GRTH binding to specific set of RNA for its regulatory action. The other interesting aspects of this study is that fewer number of genes up-regulated (53 genes) compared to those down-regulated (307 genes) associated with polysomal sites were observed in the absence of GRTH (Fig. 1 and 4, 5, 6). We have validated expression of eight of those up-regulated genes whose functional role in their association with GRTH is not evident (Fig. 7 bottom and Table 1).

Some of the IPA predicted network pathways are consistent with the loss of chromatin remodeling genes such as transition protein (Tp1/2) and protamine (1/2) which contribute to the arrest of spemiogenesis [13]. In the case of NFκB signaling, the apoptosis observed in germ cells at the metaphase of meiosis of GRTH KO might result from the down-regulation of NFκB signaling. In the case of GRTH linked ubiquitine-proteasome-HSP network, we can envision the absence of GRTH causing abnormal gene degradation/ transport and ultimately sterility. Since our present analysis is based on mRNA expression, the functionality of other predicted pathways clearly requires a major effort to elucidate the complex biological processes and this will be the subject of our future investigations. The differential microarray studies together with detailed bioinformatic analyses provide new information and overall insights into GRTH mediated gene regulation using GRTH knockout mouse as the experimental platform. As GRTH is essential for spermatid development and completion of spermatogenesis, the present study provides valuable resources for future projects on the functions of unexplored factors in the genome linked to GRTH action in male reproduction.

## Supporting Information

Figure S1 **IPA predicted network functions from a panel of differential expressed polysomal genes (mRNA) (121 down, 46 up) that are not associated with GRTH protein in GRTH^−/−^ compared to the wild type mouse testis.** **A–C**, down-regulated genes associated network. **D–F**. up-regulated genes associated network. Genes in color green (down-regulated), red (up-regulated) and uncolored (relevant biological genes to the network with no change in expression between WT and GRTH^−/−^).
(TIF)

Figure S2 **IPA predicted top network functions of overall differentially expressed genes (139 down, 51 up) in spermatocytes of GRTH^−/−^ compared to wild type mice.** Spermatocytes prepared from four different time of pooled adult KO or WT mice testis were used for microarray analysis. **A–C**. IPA predicted top score network pathway. Genes in color green (down-regulated), red (up-regulated) and uncolored (relevant biological genes to the network with no change in expression between WT and GRTH^−/−^).
(TIF)

Figure S3 **IPA predicted top network functions of overall differentially expressed genes (216 down, 326 up) in round spermatids of GRTH^−/−^ compared to wild type mice.** Round spermatids prepared from four different time of pooled adult KO or WT mice testis were used for microarray analysis. **A–D**. IPA predicted top score network pathway. Genes in color green (down-regulated), red (up-regulated) and uncolored (relevant biological genes to the network with no change in expression between WT and GRTH^−/−^).
(TIF)

Figure S4 **IPA predicted top network functions of overall differentially expressed genes (144 down, 155 up) in Leydig cells of GRTH^−/−^ compared to wild type mice.** Leydig cells prepared from four different time of pooled adult KO or WT mice testis were used for microarray analysis. **A–D**. IPA predicted top score network pathway. Genes in color green (down-regulated), red (up-regulated) and uncolored (relevant biological genes to the network with no change in expression between WT and GRTH KO).
(TIF)

Table S1 **Differentially regulated genes in testicular polysomes of GRTH KO compared to wild type mice.** **A**. List of down-regulated genes (307) in testicular polysomes of GRTH^−/−^ compared to wild type mice. **B**. List of up-regulated genes (53) in testicular polysomes of GRTH^−/−^ compared to wild type mice.
(DOCX)

Table S2 **List of genes (792) associated with GRTH protein in testicular polysomes of wild type adult mice.**
(DOC)

Table S3 **Differentially regulated genes associated with GRTH in testicular polysomes of wild type adult mice.** **A**. List of differentially down-regulated genes (186) associated with GRTH in polysomes. **B**. List of differentially up-regulated genes (7) associated with GRTH in polysomes.
(DOCX)

Table S4 **Differentially regulated genes in spermatocytes of GRTH KO compared to wild type adult mice.** **A**. List of down-regulated genes (139) in spermatocytes of GRTH^−/−^ compared to wild type mice. **B**. List of up-regulated genes (52) in spermatocytes of GRTH^−/−^ compared to wild type mice.
(DOCX)

Table S5 **Differentially regulated genes in round spermatids of GRTH KO compared to wild type adult mice.** **A**. List of down-regulated genes (216) in round spermatids of GRTH^−/−^ compared to wild type mice. **B**. List of up-regulated genes (326) in round spermatids of GRTH^−/−^ compared to wild type mice.
(DOCX)

Table S6 **Differentially regulated genes in Leydig cells of GRTH KO compared to wild type adult mice.** **A**. List of down-regulated genes (144) in Leydig cells of GRTH^−/−^ compared to wild type mice. **B**. List of down-regulated genes (155) in Leydig cells of GRTH^−/−^ compared to wild type mice.
(DOCX)

Table S7 **List of differentially expressed genes associated with GRTH in polysome of the individual testicular cells.**
(DOC)
