# Supplementary figures and images for: Role of Gonadotropin Regulated Testicular RNA Helicase (GRTH/DDX25) on Polysomal Associated mRNAs in Mouse Testis
Source: PLoS One. 2012 Mar 30;7(3):e32470. doi: 10.1371/journal.pone.0032470 (PMC3316541; doi:10.1371/journal.pone.0032470)

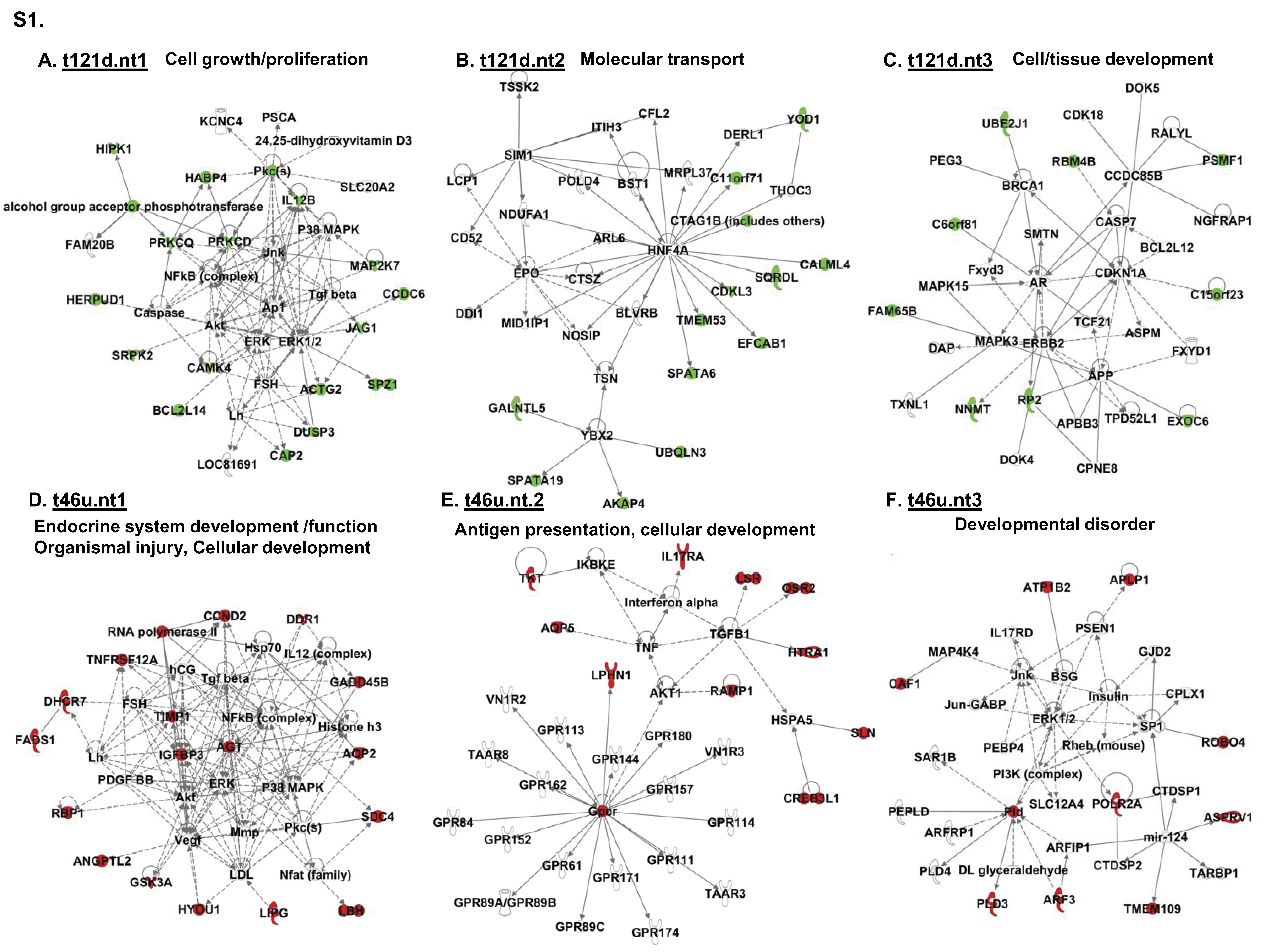

Supplement: Figure S1 — IPA predicted network functions from a panel of differential expressed polysomal genes (mRNA) (121 down, 46 up) that are not associated with GRTH protein in GRTH−/− compared to the wild type mouse testis. A–C, down-regulated genes associated network. D–F. up-regulated genes associated network. Genes in color green (down-regulated), red (up-regulated) and uncolored (relevant biological genes to the network with no change in expression between WT and GRTH−/−). (TIF) [file pone.0032470.s001.tif]

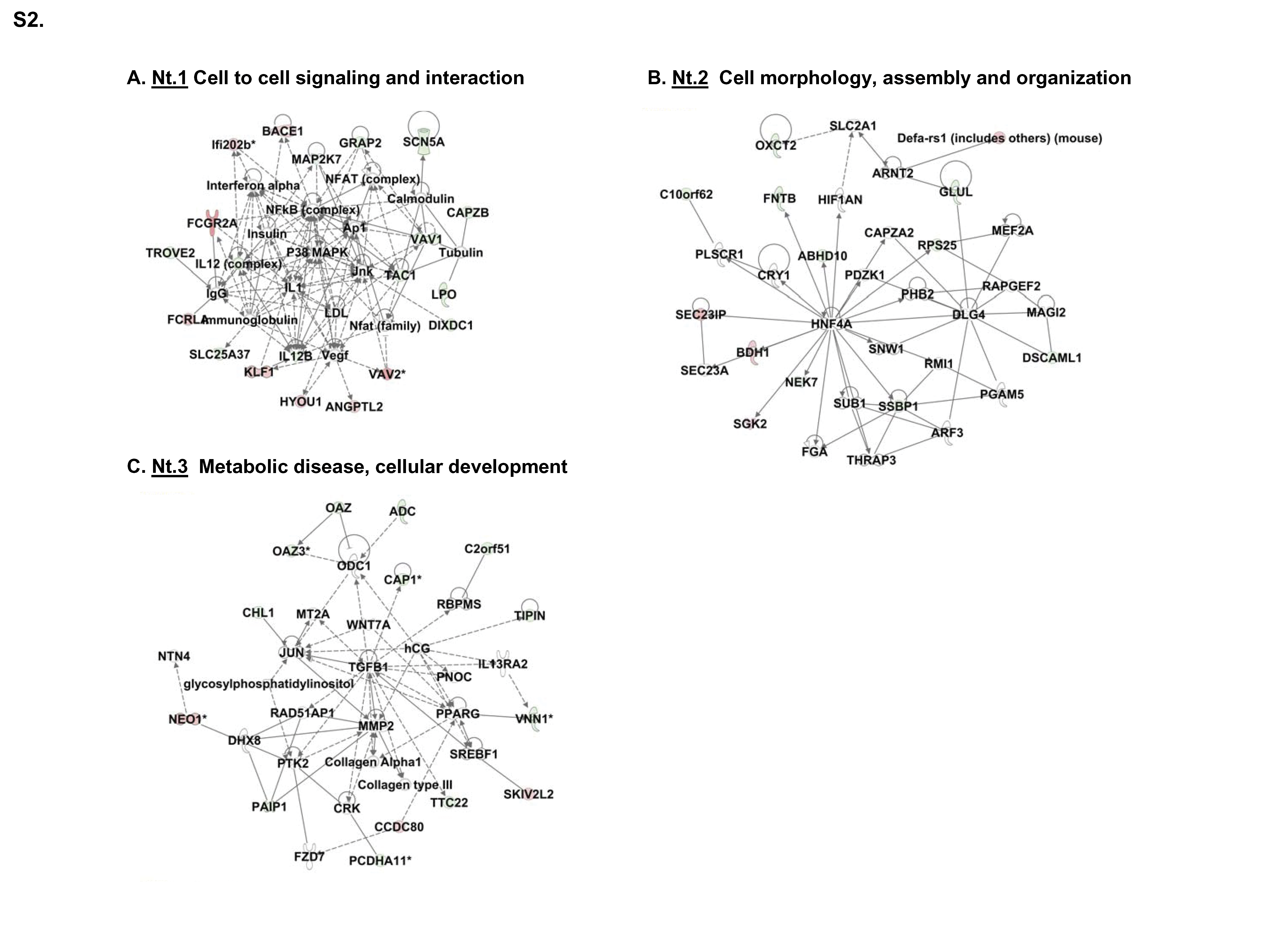

Supplement: Figure S2 — IPA predicted top network functions of overall differentially expressed genes (139 down, 51 up) in spermatocytes of GRTH−/− compared to wild type mice. Spermatocytes prepared from four different time of pooled adult KO or WT mice testis were used for microarray analysis. A–C. IPA predicted top score network pathway. Genes in color green (down-regulated), red (up-regulated) and uncolored (relevant biological genes to the network with no change in expression between WT and GRTH−/−). (TIF) [file pone.0032470.s002.tif]

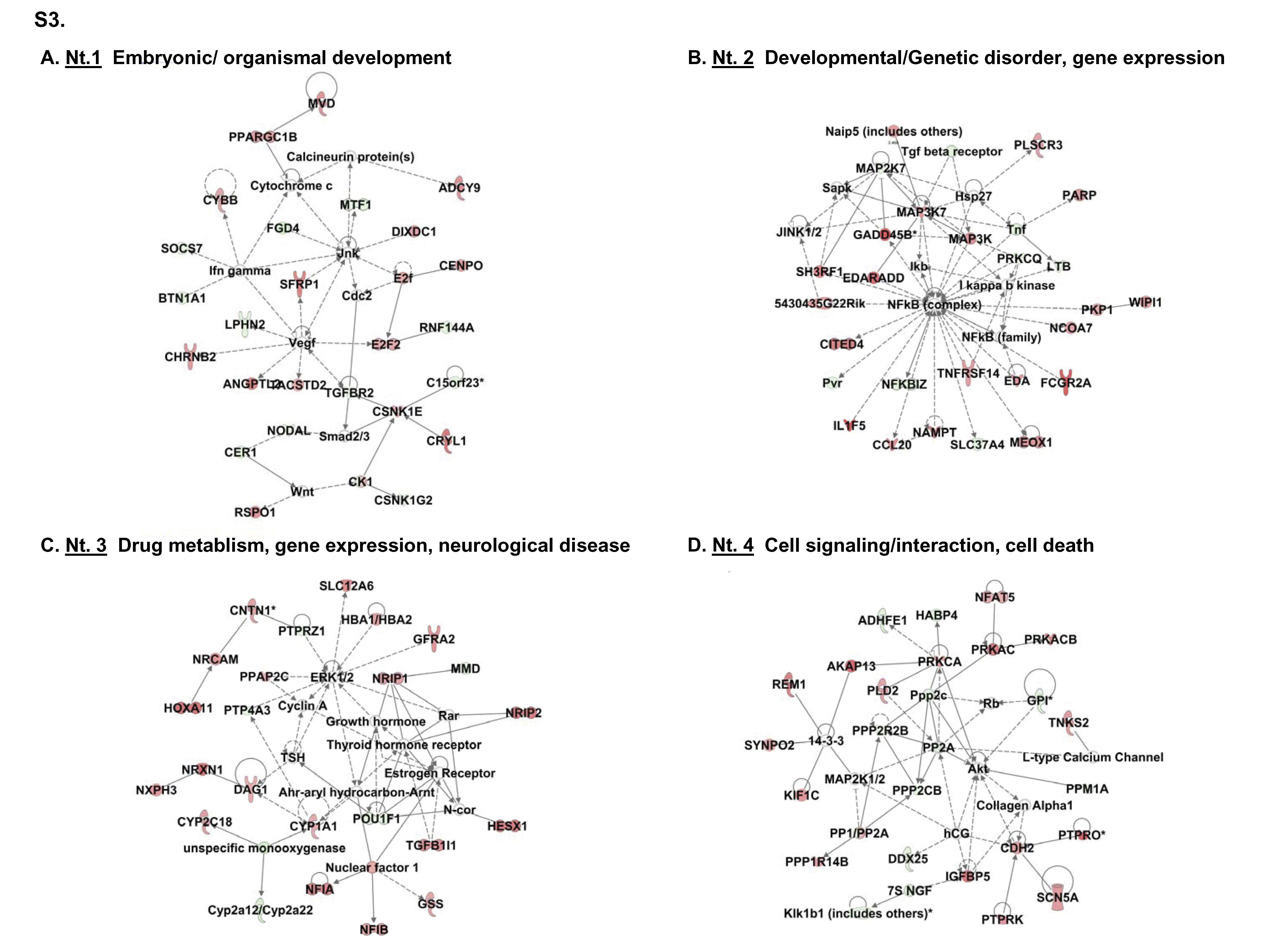

Supplement: Figure S3 — IPA predicted top network functions of overall differentially expressed genes (216 down, 326 up) in round spermatids of GRTH−/− compared to wild type mice. Round spermatids prepared from four different time of pooled adult KO or WT mice testis were used for microarray analysis. A–D. IPA predicted top score network pathway. Genes in color green (down-regulated), red (up-regulated) and uncolored (relevant biological genes to the network with no change in expression between WT and GRTH−/−). (TIF) [file pone.0032470.s003.tif]

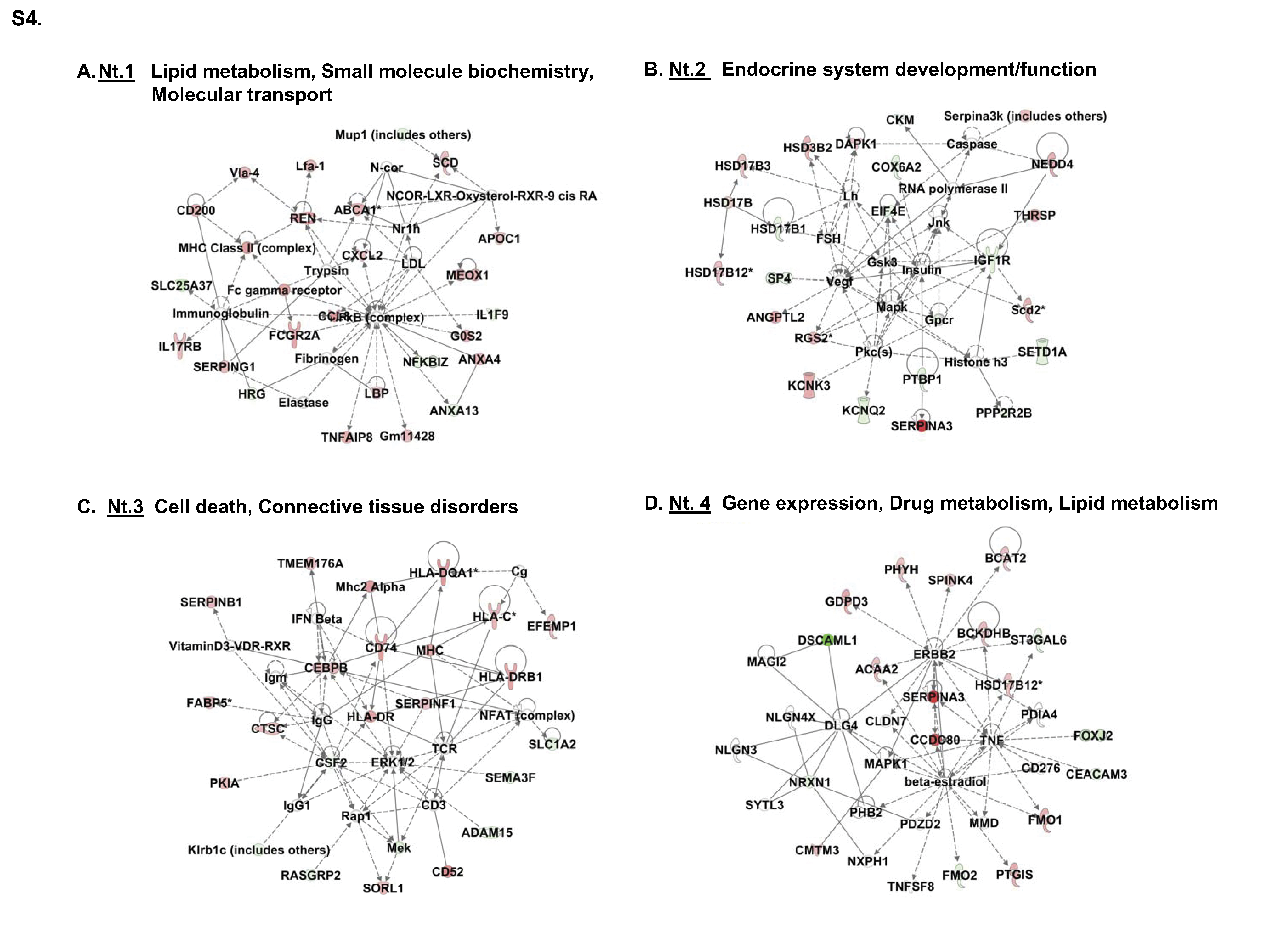

Supplement: Figure S4 — IPA predicted top network functions of overall differentially expressed genes (144 down, 155 up) in Leydig cells of GRTH−/− compared to wild type mice. Leydig cells prepared from four different time of pooled adult KO or WT mice testis were used for microarray analysis. A–D. IPA predicted top score network pathway. Genes in color green (down-regulated), red (up-regulated) and uncolored (relevant biological genes to the network with no change in expression between WT and GRTH KO). (TIF) [file pone.0032470.s004.tif]
