# Supplementary material for: Role of Gonadotropin Regulated Testicular RNA Helicase (GRTH/DDX25) on Polysomal Associated mRNAs in Mouse Testis
Source: PLoS One. 2012 Mar 30;7(3):e32470. doi: 10.1371/journal.pone.0032470 (PMC3316541; doi:10.1371/journal.pone.0032470)
Supplement: Table S2 — List of genes (792) associated with GRTH protein in testicular polysomes of wild type adult mice. (DOC) [file pone.0032470.s006.doc]

| **Table S2. List of genes (792) associated with GRTH protein in testicular polysomes of wild type adult mice** | | |
| --- | --- | --- |
|  |  |  |
| **Affymetrix ID** | **Symbol** | **Entrez Gene Name** |
| 1456857_at | 1500011B03Rik | RIKEN cDNA 1500011B03 gene |
| 1452602_a_at | 1700001C19Rik | RIKEN cDNA 1700001C19 gene |
| 1419151_at | 1700001J03Rik (includes others) | predicted gene 3415 |
| 1422722_at | 1700001K19Rik | RIKEN cDNA 1700001K19 gene |
| 1437988_x_at | 1700003E24Rik | RIKEN cDNA 1700003E24 gene |
| 1431103_at | 1700003P14Rik | RIKEN cDNA 1700003P14 gene |
| 1430284_at | 1700008P02Rik | RIKEN cDNA 1700008P02 gene |
| 1432505_at | 1700009J07Rik | RIKEN cDNA 1700009J07 gene |
| 1429747_at | 1700009N14Rik | RIKEN cDNA 1700009N14 gene |
| 1429636_at | 1700010D01Rik (includes others) | predicted gene 9043 |
| 1453346_at | 1700011L22Rik | RIKEN cDNA 1700011L22 gene |
| 1436683_at | 1700011M02Rik/Gm9112 | predicted gene 9112 |
| 1429142_at | 1700012A03Rik | RIKEN cDNA 1700012A03 gene |
| 1418303_at | 1700012L04Rik (includes others) | predicted gene 14475 |
| 1437792_at | 1700013D24Rik | RIKEN cDNA 1700013D24 gene |
| 1459995_at | 1700015G11Rik | RIKEN cDNA 1700015G11 gene |
| 1438791_at | 1700016P04Rik | RIKEN cDNA 1700016P04 gene |
| 1441861_at | 1700018G05Rik | RIKEN cDNA 1700018G05 gene |
| 1429513_at | 1700019M22Rik/RP23-438H3.2 | RIKEN cDNA 1700019M22 gene |
| 1447710_at | 1700019P21Rik | RIKEN cDNA 1700019P21 gene |
| 1430524_at | 1700020N15Rik/Gm6812 | RIKEN cDNA 1700020N15 gene |
| 1453383_at | 1700022F17Rik | RIKEN cDNA 1700022F17 gene |
| 1429855_at | 1700023I07Rik | protein phosphatase 1, regulatory (inhibitor) subunit 2 pseudogene |
| 1430261_at | 1700024J04Rik | RIKEN cDNA 1700024J04 gene |
| 1429828_at | 1700024P04Rik | RIKEN cDNA 1700024P04 gene |
| 1458398_at | 1700027F06Rik | RIKEN cDNA 1700027F06 gene |
| 1450999_a_at | 1700029H14Rik | RIKEN cDNA 1700029H14 gene |
| 1457057_at | 1700042G07Rik | RIKEN cDNA 1700042G07 gene |
| 1438384_at | 1700047L15Rik | SPANX family, member N4 |
| 1440768_x_at | 1700052I22Rik | RIKEN cDNA 1700052I22 gene |
| 1441773_at | 1700061N14Rik | RIKEN cDNA 1700061N14 gene |
| 1457421_at | 1700063D05Rik | RIKEN cDNA 1700063D05 gene |
| 1432514_at | 1700066J24Rik | RIKEN cDNA 1700066J24 gene |
| 1419337_at | 1700080E11Rik | RIKEN cDNA 1700080E11 gene |
| 1453447_at | 1700109H08Rik | RIKEN cDNA 1700109H08 gene |
| 1457050_at | 1700129O19Rik | RIKEN cDNA 1700129O19 gene |
| 1428258_at | 2010107E04Rik | RIKEN cDNA 2010107E04 gene |
| 1460478_at | 2200002J24Rik | RIKEN cDNA 2200002J24 gene |
| 1435237_at | 2310009A05Rik | RIKEN cDNA 2310009A05 gene |
| 1436362_x_at | 2700079J08Rik | RIKEN cDNA 2700079J08 gene |
| 1429771_at | 3110073H01Rik | RIKEN cDNA 3110073H01 gene |
| 1430624_at | 4921511M17Rik (includes others) | predicted pseudogene 5645 |
| 1419718_at | 4921530L21Rik | RIKEN cDNA 4921530L21 gene |
| 1445661_at | 4930406D18Rik | RIKEN cDNA 4930406D18 gene |
| 1430872_at | 4930412O13Rik | RIKEN cDNA 4930412O13 gene |
| 1441651_at | 4930414N06Rik | RIKEN cDNA 4930414N06 gene |
| 1432078_at | 4930453L07Rik | RIKEN cDNA 4930453L07 gene |
| 1453838_at | 4930471G03Rik | RIKEN cDNA 4930471G03 gene |
| 1433326_at | 4930488N24Rik (includes others) | predicted gene 7358 |
| 1431184_a_at | 4930503B20Rik | RIKEN cDNA 4930503B20 gene |
| 1454133_s_at | 4930523O13Rik | RIKEN cDNA 4930523O13 gene |
| 1433421_at | 4930527J03Rik | RIKEN cDNA 4930527J03 gene |
| 1421367_at | 4930549C01Rik | RIKEN cDNA 4930549C01 gene |
| 1431673_at | 4930557A04Rik | RIKEN cDNA 4930557A04 gene |
| 1431855_at | 4930570E03Rik | RIKEN cDNA 4930570E03 gene |
| 1430063_at | 4930571K23Rik | RIKEN cDNA 4930571K23 gene |
| 1429551_at | 4930579G22Rik | RIKEN cDNA 4930579G22 gene |
| 1430016_at | 4930584F24Rik | RIKEN cDNA 4930584F24 gene |
| 1432871_at | 4932429P19Rik | RIKEN cDNA 4932429P19 gene |
| 1429263_at | 4933425D22Rik | RIKEN cDNA 4933425D22 gene |
| 1420787_at | 4933436I01Rik | RIKEN cDNA 4933436I01 gene |
| 1430441_at | 4933438K21Rik | RIKEN cDNA 4933438K21 gene |
| 1429840_at | 4933439G12Rik | RIKEN cDNA 4933439G12 gene |
| 1451712_at | 8030411F24Rik | RIKEN cDNA 8030411F24 gene |
| 1432531_at | 9230104L09Rik | RIKEN cDNA 9230104L09 gene |
| 1453429_at | 9530057J20Rik | RIKEN cDNA 9530057J20 gene |
| 1421840_at | ABCA1 | ATP-binding cassette, sub-family A (ABC1), member 1 |
| 1422906_at | ABCG2 | ATP-binding cassette, sub-family G (WHITE), member 2 |
| 1436984_at | ABI2 | abl-interactor 2 |
| 1428236_at | ACBD5 | acyl-CoA binding domain containing 5 |
| 1418878_at | ACRV1 | acrosomal vesicle protein 1 |
| 1439000_at | ACSBG2 | acyl-CoA synthetase bubblegum family member 2 |
| 1422526_at | ACSL1 | acyl-CoA synthetase long-chain family member 1 |
| 1416454_s_at | ACTG2 | actin, gamma 2, smooth muscle, enteric |
| 1437382_at | ACVR2A | activin A receptor, type IIA |
| 1450095_a_at | ACYP1 | acylphosphatase 1, erythrocyte (common) type |
| 1428103_at | ADAM10 | ADAM metallopeptidase domain 10 |
| 1449513_at | Adam24 | a disintegrin and metallopeptidase domain 24 (testase 1) |
| 1425395_at | Adam26a/Adam26b | a disintegrin and metallopeptidase domain 26A (testase 3) |
| 1428030_at | Adam34 (includes others) | predicted gene 5347 |
| 1456368_at | Adam4/Gm4787 | a disintegrin and metallopeptidase domain 4 |
| 1436130_s_at | ADAM6 | ADAM metallopeptidase domain 6 (pseudogene) |
| 1416094_at | ADAM9 | ADAM metallopeptidase domain 9 |
| 1439649_at | ADC | arginine decarboxylase |
| 1416185_a_at | ADH5 | alcohol dehydrogenase 5 (class III), chi polypeptide |
| 1424729_at | ADIG | adipogenin |
| 1451311_a_at | ADIPOR1 | adiponectin receptor 1 |
| 1438292_x_at | ADK | adenosine kinase |
| 1429609_at | ADORA3 | adenosine A3 receptor |
| 1428821_at | AGPAT2 | 1-acylglycerol-3-phosphate O-acyltransferase 2 (lysophosphatidic acid acyltransferase, beta) |
| 1434390_at | AI503316 | expressed sequence AI503316 |
| 1418204_s_at | AIF1 | allograft inflammatory factor 1 |
| 1418279_a_at | AKAP1 | A kinase (PRKA) anchor protein 1 |
| 1457541_at | AKAP14 | A kinase (PRKA) anchor protein 14 |
| 1435769_at | AKAP9 | A kinase (PRKA) anchor protein (yotiao) 9 |
| 1448894_at | AKR1B15 | aldo-keto reductase family 1, member B15 |
| 1456589_x_at | ALLC | allantoicase |
| 1427989_at | AMHR2 | anti-Mullerian hormone receptor, type II |
| 1433521_at | ANKRD13C | ankyrin repeat domain 13C |
| 1430395_at | ANKRD45 | ankyrin repeat domain 45 |
| 1460330_at | ANXA3 | annexin A3 |
| 1460036_at | AP1S2 | adaptor-related protein complex 1, sigma 2 subunit |
| 1420621_a_at | APP | amyloid beta (A4) precursor protein |
| 1417828_at | AQP8 | aquaporin 8 |
| 1448129_at | ARPC5 | actin related protein 2/3 complex, subunit 5, 16kDa |
| 1429788_at | ARPM1 | actin related protein M1 |
| 1422608_at | ARPP19 | cAMP-regulated phosphoprotein, 19kDa |
| 1420468_at | ASB17 | ankyrin repeat and SOCS box containing 17 |
| 1426309_at | ASB9 | ankyrin repeat and SOCS box containing 9 |
| 1444668_at | Astx | amplified spermatogenic transcripts X encoded |
| 1451376_at | ATL3 | atlastin GTPase 3 |
| 1447851_x_at | ATP10A | ATPase, class V, type 10A |
| 1451388_a_at | ATP11B | ATPase, class VI, type 11B |
| 1434513_at | ATP13A3 | ATPase type 13A3 |
| 1443823_s_at | ATP1A2 | ATPase, Na+/K+ transporting, alpha 2 polypeptide |
| 1439036_a_at | ATP1B1 | ATPase, Na+/K+ transporting, beta 1 polypeptide |
| 1437738_at | ATP2C1 | ATPase, Ca++ transporting, type 2C, member 1 |
| 1437688_x_at | ATP6AP2 | ATPase, H+ transporting, lysosomal accessory protein 2 |
| 1447802_x_at | AV099323 | expressed sequence AV099323 |
| 1434671_at | B230337E12Rik | RIKEN cDNA B230337E12 gene |
| 1452428_a_at | B2M | beta-2-microglobulin |
| 1443967_at | BANF2 | barrier to autointegration factor 2 |
| 1428572_at | BASP1 | brain abundant, membrane attached signal protein 1 |
| 1448007_at | BAZ2B | bromodomain adjacent to zinc finger domain, 2B |
| 1429762_a_at | BBS5 | Bardet-Biedl syndrome 5 |
| 1439446_at | BC048507 | cDNA sequence BC048507 |
| 1435331_at | BC094916/Pyhin1 | pyrin and HIN domain family, member 1 |
| 1456279_a_at | BCAP31 | B-cell receptor-associated protein 31 |
| 1434284_at | BDP1 | B double prime 1, subunit of RNA polymerase III transcription initiation factor IIIB |
| 1428209_at | BEX4 | brain expressed, X-linked 4 |
| 1419562_at | BIRC6 | baculoviral IAP repeat containing 6 |
| 1428744_s_at | BRI3BP | BRI3 binding protein |
| 1449007_at | BTG3 | BTG family, member 3 |
| 1450846_at | BZW1 | basic leucine zipper and W2 domains 1 |
| 1452802_at | C10orf62 | chromosome 10 open reading frame 62 |
| 1430762_at | C11orf88 | chromosome 11 open reading frame 88 |
| 1438979_s_at | C11orf94 | chromosome 11 open reading frame 94 |
| 1433978_at | C12orf29 | chromosome 12 open reading frame 29 |
| 1430140_at | C12orf50 | chromosome 12 open reading frame 50 |
| 1416522_a_at | C12orf57 | chromosome 12 open reading frame 57 |
| 1452840_at | C12orf75 | chromosome 12 open reading frame 75 |
| 1453407_at | C13orf16 | chromosome 13 open reading frame 16 |
| 1428593_at | C13orf27 | chromosome 13 open reading frame 27 |
| 1430314_at | C14orf148 | chromosome 14 open reading frame 148 |
| 1431393_at | C14orf39 | chromosome 14 open reading frame 39 |
| 1431648_at | C16orf73 | chromosome 16 open reading frame 73 |
| 1420548_a_at | C16orf88 | chromosome 16 open reading frame 88 |
| 1430121_at | C17orf105 | chromosome 17 open reading frame 105 |
| 1430091_at | C17orf98 | chromosome 17 open reading frame 98 |
| 1419274_at | C19orf2 | chromosome 19 open reading frame 2 |
| 1418702_a_at | C19orf60 | chromosome 19 open reading frame 60 |
| 1448505_at | C1D/C1DP3 | C1D nuclear receptor corepressor |
| 1430674_at | C1orf100 | chromosome 1 open reading frame 100 |
| 1455126_x_at | C1orf151 | chromosome 1 open reading frame 151 |
| 1453337_at | C1orf173 | chromosome 1 open reading frame 173 |
| 1431104_at | C1orf185 | chromosome 1 open reading frame 185 |
| 1439230_at | C1orf65 | chromosome 1 open reading frame 65 |
| 1429544_at | C20orf106 | chromosome 20 open reading frame 106 |
| 1430422_at | C20orf141 | chromosome 20 open reading frame 141 |
| 1452863_at | C20orf144 | chromosome 20 open reading frame 144 |
| 1429292_a_at | C20orf54 | chromosome 20 open reading frame 54 |
| 1432308_at | C20orf71 | chromosome 20 open reading frame 71 |
| 1454202_a_at | C22orf33 | chromosome 22 open reading frame 33 |
| 1430892_at | C2orf51 | chromosome 2 open reading frame 51 |
| 1449358_at | C2orf65 | chromosome 2 open reading frame 65 |
| 1432404_at | C2orf73 | chromosome 2 open reading frame 73 |
| 1430368_s_at | C2orf88 | chromosome 2 open reading frame 88 |
| 1457928_at | C3orf30 | chromosome 3 open reading frame 30 |
| 1453879_at | C4orf36 | chromosome 4 open reading frame 36 |
| 1431447_at | C4orf40 | chromosome 4 open reading frame 40 |
| 1453168_at | C4orf47 | chromosome 4 open reading frame 47 |
| 1420774_a_at | C4orf49 | chromosome 4 open reading frame 49 |
| 1424005_at | C5orf24 | chromosome 5 open reading frame 24 |
| 1441883_at | C5orf32 | chromosome 5 open reading frame 32 |
| 1452737_at | C5orf43 | chromosome 5 open reading frame 43 |
| 1420574_at | C5orf48 | chromosome 5 open reading frame 48 |
| 1429989_at | C5orf50 | chromosome 5 open reading frame 50 |
| 1455538_at | C5orf53 | chromosome 5 open reading frame 53 |
| 1426964_at | C6orf115 | chromosome 6 open reading frame 115 |
| 1416608_a_at | C6orf89 | chromosome 6 open reading frame 89 |
| 1453142_at | C7orf31 | chromosome 7 open reading frame 31 |
| 1430044_at | C7orf72 | chromosome 7 open reading frame 72 |
| 1436523_s_at | C8orf59 | chromosome 8 open reading frame 59 |
| 1434528_at | C8orf85 | chromosome 8 open reading frame 85 |
| 1437140_at | C9orf128 | chromosome 9 open reading frame 128 |
| 1459890_s_at | C9orf16 | chromosome 9 open reading frame 16 |
| 1424795_a_at | C9orf50 | chromosome 9 open reading frame 50 |
| 1429744_at | CABS1 | calcium-binding protein, spermatid-specific 1 |
| 1417378_at | CADM1 | cell adhesion molecule 1 |
| 1423807_a_at | Calm1 (includes others) | calmodulin 1 |
| 1453233_s_at | CALR3 | calreticulin 3 |
| 1441845_at | CAPS2 | calcyphosine 2 |
| 1418556_at | CAPZA3 | capping protein (actin filament) muscle Z-line, alpha 3 |
| 1415976_a_at | CARHSP1 | calcium regulated heat stable protein 1, 24kDa |
| 1433779_at | CASC4 | cancer susceptibility candidate 4 |
| 1439421_x_at | CBX3 | chromobox homolog 3 |
| 1451166_a_at | CCDC101 | coiled-coil domain containing 101 |
| 1458438_at | CCDC122 | coiled-coil domain containing 122 |
| 1440825_s_at | CCDC28A | coiled-coil domain containing 28A |
| 1433995_s_at | Ccdc50 | coiled-coil domain containing 50 |
| 1428965_at | CCDC54 | coiled-coil domain containing 54 |
| 1429852_at | CCDC57 | coiled-coil domain containing 57 |
| 1449487_at | CCDC70 | coiled-coil domain containing 70 |
| 1428505_at | CCDC90B | coiled-coil domain containing 90B |
| 1418459_at | CCDC91 | coiled-coil domain containing 91 |
| 1426067_x_at | CCT3 | chaperonin containing TCP1, subunit 3 (gamma) |
| 1437962_at | CCT8L2/LOC155100 | chaperonin containing TCP1, subunit 8 (theta)-like 1 |
| 1420907_at | CD2AP | CD2-associated protein |
| 1417224_a_at | CD2BP2 | CD2 (cytoplasmic tail) binding protein 2 |
| 1448919_at | CD302 | CD302 molecule |
| 1450883_a_at | CD36 | CD36 molecule (thrombospondin receptor) |
| 1421586_a_at | CD46 | CD46 molecule, complement regulatory protein |
| 1429830_a_at | Cd59a | CD59a antigen |
| 1419226_at | CD96 | CD96 molecule |
| 1436715_s_at | CDIPT | CDP-diacylglycerol--inositol 3-phosphatidyltransferase |
| 1415956_a_at | CDK16 | cyclin-dependent kinase 16 |
| 1435509_x_at | CDK2AP1 | cyclin-dependent kinase 2 associated protein 1 |
| 1448983_at | CDRT4 | CMT1A duplicated region transcript 4 |
| 1451100_a_at | CDV3 | CDV3 homolog (mouse) |
| 1448935_at | CDYL | chromodomain protein, Y-like |
| 1418264_at | CENPK | centromere protein K |
| 1432522_s_at | CEP112 | centrosomal protein 112kDa |
| 1420579_s_at | CFTR | cystic fibrosis transmembrane conductance regulator (ATP-binding cassette sub-family C, member 7) |
| 1451092_a_at | CHADL | chondroadherin-like |
| 1436990_s_at | CHCHD10 | coiled-coil-helix-coiled-coil-helix domain containing 10 |
| 1428161_a_at | CHCHD2 | coiled-coil-helix-coiled-coil-helix domain containing 2 |
| 1451504_at | CHCHD3 | coiled-coil-helix-coiled-coil-helix domain containing 3 |
| 1417795_at | CHL1 | cell adhesion molecule with homology to L1CAM (close homolog of L1) |
| 1435926_at | CHML | choroideremia-like (Rab escort protein 2) |
| 1432270_a_at | CHMP5 | chromatin modifying protein 5 |
| 1428574_a_at | Chn2 | chimerin (chimaerin) 2 |
| 1416698_a_at | CKS1B | CDC28 protein kinase regulatory subunit 1B |
| 1417457_at | CKS2 | CDC28 protein kinase regulatory subunit 2 |
| 1438366_x_at | CLCN3 | chloride channel 3 |
| 1416003_at | CLDN11 | claudin 11 |
| 1429925_at | CLEC12B | C-type lectin domain family 12, member B |
| 1425407_s_at | CLEC4A | C-type lectin domain family 4, member A |
| 1416656_at | CLIC1 | chloride intracellular channel 1 |
| 1425321_a_at | CLMN | calmin (calponin-like, transmembrane) |
| 1416541_at | CLPB | ClpB caseinolytic peptidase B homolog (E. coli) |
| 1418626_a_at | CLU | clusterin |
| 1426662_at | CMAS | cytidine monophosphate N-acetylneuraminic acid synthetase |
| 1423073_at | CMPK1 | cytidine monophosphate (UMP-CMP) kinase 1, cytosolic |
| 1423792_a_at | CMTM6 | CKLF-like MARVEL transmembrane domain containing 6 |
| 1423727_at | CNIH | cornichon homolog (Drosophila) |
| 1437783_x_at | CNPY2 | canopy 2 homolog (zebrafish) |
| 1425234_at | COL20A1 | collagen, type XX, alpha 1 |
| 1420383_a_at | COL4A3BP | collagen, type IV, alpha 3 (Goodpasture antigen) binding protein |
| 1437039_at | COPS2 | COP9 constitutive photomorphogenic homolog subunit 2 (Arabidopsis) |
| 1434435_s_at | COX17 | COX17 cytochrome c oxidase assembly homolog (S. cerevisiae) |
| 1417417_a_at | COX6A1 | cytochrome c oxidase subunit VIa polypeptide 1 |
| 1416565_at | COX6B1 | cytochrome c oxidase subunit VIb polypeptide 1 (ubiquitous) |
| 1434491_a_at | COX6C | cytochrome c oxidase subunit VIc |
| 1416970_a_at | COX7A2 | cytochrome c oxidase subunit VIIa polypeptide 2 (liver) |
| 1448222_x_at | COX8A | cytochrome c oxidase subunit VIIIA (ubiquitous) |
| 1415949_at | CPE | carboxypeptidase E |
| 1445868_at | CPEB3 | cytoplasmic polyadenylation element binding protein 3 |
| 1428231_at | CPSF6 | cleavage and polyadenylation specific factor 6, 68kDa |
| 1415948_at | CREG1 | cellular repressor of E1A-stimulated genes 1 |
| 1431930_x_at | CRLS1 | cardiolipin synthase 1 |
| 1423370_a_at | CSNK1G2 | casein kinase 1, gamma 2 |
| 1424627_at | Cst12 | cystatin 12 |
| 1429424_at | Cst13 | cystatin 13 |
| 1424479_at | CST8 | cystatin 8 (cystatin-related epididymal specific) |
| 1449285_at | CST9L | cystatin 9-like |
| 1422506_a_at | CSTB | cystatin B (stefin B) |
| 1437807_x_at | CTNNA1 | catenin (cadherin-associated protein), alpha 1, 102kDa |
| 1451310_a_at | CTSL2 | cathepsin L2 |
| 1433908_a_at | CTTN | cortactin |
| 1417453_at | CUL4B | cullin 4B |
| 1420424_at | CXorf27 | chromosome X open reading frame 27 |
| 1422483_a_at | Cycs/Gm10053 | cytochrome c, somatic |
| 1421591_at | CYLC1 | cylicin, basic protein of sperm head cytoskeleton 1 |
| 1456192_x_at | Cypt1 (includes others) | cysteine-rich perinuclear theca 1 |
| 1429374_at | Cypt12 | cysteine-rich perinuclear theca 12 |
| 1453568_at | DAPL1 | death associated protein-like 1 |
| 1419542_at | DAZ2 | deleted in azoospermia 2 |
| 1452079_s_at | DCUN1D1 | DCN1, defective in cullin neddylation 1, domain containing 1 (S. cerevisiae) |
| 1418263_at | DDX25 | DEAD (Asp-Glu-Ala-Asp) box polypeptide 25 |
| 1456076_at | DEFB119 | defensin, beta 119 |
| 1452659_at | DEK | DEK oncogene |
| 1437723_s_at | DERL1 | Der1-like domain family, member 1 |
| 1422677_at | DGAT2 | diacylglycerol O-acyltransferase 2 |
| 1426465_at | DLGAP4 | discs, large (Drosophila) homolog-associated protein 4 |
| 1423582_at | DMRT1 | doublesex and mab-3 related transcription factor 1 |
| 1429573_at | DMRTC1/DMRTC1B | DMRT-like family C1B |
| 1418592_at | DNAJA4 | DnaJ (Hsp40) homolog, subfamily A, member 4 |
| 1431734_a_at | DNAJB4 | DnaJ (Hsp40) homolog, subfamily B, member 4 |
| 1450991_at | DNAJB7 | DnaJ (Hsp40) homolog, subfamily B, member 7 |
| 1446928_at | DNAJC17 | DnaJ (Hsp40) homolog, subfamily C, member 17 |
| 1418725_at | DNAJC5B | DnaJ (Hsp40) homolog, subfamily C, member 5 beta |
| 1438789_s_at | DPYSL3 | dihydropyrimidinase-like 3 |
| 1452912_at | DSCC1 | defective in sister chromatid cohesion 1 homolog (S. cerevisiae) |
| 1453400_at | DYDC2 | DPY30 domain containing 2 |
| 1417339_a_at | DYNLL1 | dynein, light chain, LC8-type 1 |
| 1418371_at | DYNLL2 | dynein, light chain, LC8-type 2 |
| 1428257_s_at | DYNLRB1 | dynein, light chain, roadblock-type 1 |
| 1449929_at | DYNLT3 | dynein, light chain, Tctex-type 3 |
| 1439439_x_at | EEF1D | eukaryotic translation elongation factor 1 delta (guanine nucleotide exchange protein) |
| 1429445_at | EFCAB3 | EF-hand calcium binding domain 3 |
| 1429746_at | EFCAB9 | EF-hand calcium binding domain 9 |
| 1437478_s_at | EFHD2 | EF-hand domain family, member D2 |
| 1438657_x_at | EG667723/Ptp4a1 | protein tyrosine phosphatase 4a1 |
| 1423798_a_at | EIF1 | eukaryotic translation initiation factor 1 |
| 1419736_a_at | EIF1AX | eukaryotic translation initiation factor 1A, X-linked |
| 1448820_a_at | EIF2S2 | eukaryotic translation initiation factor 2, subunit 2 beta, 38kDa |
| 1417210_at | Eif2s3y (includes EG:171792) | eukaryotic translation initiation factor 2, subunit 3, structural gene Y-linked |
| 1444010_at | EIF4E | eukaryotic translation initiation factor 4E |
| 1434976_x_at | EIF4EBP1 | eukaryotic translation initiation factor 4E binding protein 1 |
| 1434604_at | EIF5B | eukaryotic translation initiation factor 5B |
| 1434765_at | EP300 | E1A binding protein p300 |
| 1428011_a_at | ERBB2IP | erbb2 interacting protein |
| 1423972_at | ETFA | electron-transfer-flavoprotein, alpha polypeptide |
| 1436817_at | EXOC5 | exocyst complex component 5 |
| 1420115_at | EXOSC8 | exosome component 8 |
| 1449269_at | F5 | coagulation factor V (proaccelerin, labile factor) |
| 1431788_at | FABP12 | fatty acid binding protein 12 |
| 1416021_a_at | FABP5 | fatty acid binding protein 5 (psoriasis-associated) |
| 1421804_at | FABP9 | fatty acid binding protein 9, testis |
| 1418029_at | FAIM | Fas apoptotic inhibitory molecule |
| 1452706_a_at | FAM116B | family with sequence similarity 116, member B |
| 1433572_a_at | FAM120A | family with sequence similarity 120A |
| 1453955_a_at | Fam122c | family with sequence similarity 122, member C |
| 1439422_a_at | FAM132A | family with sequence similarity 132, member A |
| 1450505_a_at | FAM134B | family with sequence similarity 134, member B |
| 1428930_at | FAM156A/FAM156B | family with sequence similarity 156, member A |
| 1453026_at | FAM166A | family with sequence similarity 166, member A |
| 1453057_at | Fam187b | family with sequence similarity 187, member B |
| 1431972_a_at | FAM190B | family with sequence similarity 190, member B |
| 1423829_at | FAM49B | family with sequence similarity 49, member B |
| 1436948_a_at | FAM70A | family with sequence similarity 70, member A |
| 1429397_a_at | FAM71D | family with sequence similarity 71, member D |
| 1434455_at | FBXO44 | F-box protein 44 |
| 1437468_x_at | FBXW11 | F-box and WD repeat domain containing 11 |
| 1449332_at | FHL5 | four and a half LIM domains 5 |
| 1416858_a_at | FKBP3 | FK506 binding protein 3, 25kDa |
| 1416803_at | FKBP7 | FK506 binding protein 7 |
| 1437687_x_at | FKBP9 | FK506 binding protein 9, 63 kDa |
| 1434739_at | FMR1NB | fragile X mental retardation 1 neighbor |
| 1419924_at | FNIP1 | folliculin interacting protein 1 |
| 1418948_at | FSCN3 | fascin homolog 3, actin-bundling protein, testicular (Strongylocentrotus purpuratus) |
| 1418364_a_at | FTL | ferritin, light polypeptide |
| 1430829_s_at | FTO | fat mass and obesity associated |
| 1419016_at | FUNDC2 | FUN14 domain containing 2 |
| 1421374_a_at | FXYD1 | FXYD domain containing ion transport regulator 1 |
| 1417558_at | FYN | FYN oncogene related to SRC, FGR, YES |
| 1449974_at | GAPDHS | glyceraldehyde-3-phosphate dehydrogenase, spermatogenic |
| 1423569_at | GATM | glycine amidinotransferase (L-arginine:glycine amidinotransferase) |
| 1419595_a_at | GGH | gamma-glutamyl hydrolase (conjugase, folylpolygammaglutamyl hydrolase) |
| 1415800_at | GJA1 | gap junction protein, alpha 1, 43kDa |
| 1437680_x_at | GLRX2 | glutaredoxin 2 |
| 1431451_at | GLT6D1 | glycosyltransferase 6 domain containing 1 |
| 1426235_a_at | GLUL | glutamate-ammonia ligase |
| 1416624_a_at | Gm11808/Uba52 | ubiquitin A-52 residue ribosomal protein fusion product 1 |
| 1460582_x_at | Gm14226 | predicted gene 14226 |
| 1429323_at | Gm16440 (includes others) | predicted gene 16440 |
| 1428301_at | Gm2897 | predicted gene 2897 |
| 1426088_at | Gm4076 | predicted gene 4076 |
| 1423455_at | Gm4617/Ptma | prothymosin alpha |
| 1453217_at | Gm4836 (includes others) | predicted gene 4836 |
| 1448232_x_at | Gm5620 | predicted gene 5620 |
| 1449196_a_at | Gm6111/Rps27a | ribosomal protein S27A |
| 1439149_s_at | Gm9999 | predicted gene 9999 |
| 1454959_s_at | GNAI1 | guanine nucleotide binding protein (G protein), alpha inhibiting activity polypeptide 1 |
| 1450186_s_at | Gnas (mouse) | GNAS (guanine nucleotide binding protein, alpha stimulating) complex locus |
| 1454696_at | GNB1 | guanine nucleotide binding protein (G protein), beta polypeptide 1 |
| 1419499_at | GPAM | glycerol-3-phosphate acyltransferase, mitochondrial |
| 1434814_x_at | GPI | glucose-6-phosphate isomerase |
| 1460671_at | GPX1 | glutathione peroxidase 1 |
| 1439150_x_at | Grtp1 (mouse) | GH regulated TBC protein 1 |
| 1436991_x_at | GSN | gelsolin |
| 1416368_at | Gsta4 | glutathione S-transferase, alpha 4 |
| 1421040_a_at | GSTA5 | glutathione S-transferase alpha 5 |
| 1449575_a_at | Gstp1 (includes others) | glutathione S-transferase, pi 1 |
| 1430181_at | H1FNT | H1 histone family, member N, testis-specific |
| 1434127_a_at | H3F3C | H3 histone, family 3C |
| 1455972_x_at | HADH | hydroxyacyl-CoA dehydrogenase |
| 1428361_x_at | HBA1/HBA2 | hemoglobin, alpha 1 |
| 1428823_at | HDDC2 | HD domain containing 2 |
| 1415982_at | HERPUD2 | HERPUD family member 2 |
| 1448512_at | HILS1 | histone linker H1 domain, spermatid-specific 1 |
| 1424017_a_at | HINT1 | histidine triad nucleotide binding protein 1 |
| 1438009_at | HIST1H2AB/HIST1H2AE | histone cluster 1, H2ae |
| 1427762_x_at | HIST1H2BD | histone cluster 1, H2bd |
| 1418072_at | HIST1H2BJ/HIST1H2BK | histone cluster 1, H2bk |
| 1418367_x_at | HIST2H2AC | histone cluster 2, H2ac |
| 1422948_s_at | Hist2h4 (includes others) | histone cluster 2, H4 |
| 1438858_x_at | HLA-DQA1 | major histocompatibility complex, class II, DQ alpha 1 |
| 1429349_at | HMGB4 | high mobility group box 4 |
| 1455897_x_at | Hmgn1 | high mobility group nucleosomal binding domain 1 |
| 1433507_a_at | Hmgn4 | high mobility group nucleosomal binding domain 2, pseudogene 1 |
| 1452774_at | HNRNPA3 | heterogeneous nuclear ribonucleoprotein A3 |
| 1428662_a_at | HOPX | HOP homeobox |
| 1438902_a_at | HSP90AA1 | heat shock protein 90kDa alpha (cytosolic), class A member 1 |
| 1419625_at | HSPA1L | heat shock 70kDa protein 1-like |
| 1418253_a_at | HSPA4L | heat shock 70kDa protein 4-like |
| 1422943_a_at | HSPB1 | heat shock 27kDa protein 1 |
| 1450668_s_at | HSPE1 | heat shock 10kDa protein 1 (chaperonin 10) |
| 1423754_at | IFITM3 | interferon induced transmembrane protein 3 |
| 1416067_at | IFRD1 | interferon-related developmental regulator 1 |
| 1423584_at | IGFBP7 | insulin-like growth factor binding protein 7 |
| 1458481_at | IL2RG | interleukin 2 receptor, gamma |
| 1437289_at | IMPAD1 | inositol monophosphatase domain containing 1 |
| 1418984_at | INADL | InaD-like (Drosophila) |
| 1422728_at | INHA | inhibin, alpha |
| 1423274_at | INTS6 | integrator complex subunit 6 |
| 1418056_at | Iqcf3 | IQ motif containing F3 |
| 1456133_x_at | ITGB5 | integrin, beta 5 |
| 1423021_s_at | JAK3 | Janus kinase 3 |
| 1424283_at | JTB | jumping translocation breakpoint |
| 1449117_at | JUND | jun D proto-oncogene |
| 1429666_at | KCTD16 | potassium channel tetramerisation domain containing 16 |
| 1419827_s_at | KIF17 | kinesin family member 17 |
| 1415855_at | KITLG | KIT ligand |
| 1451021_a_at | KLF5 | Kruppel-like factor 5 (intestinal) |
| 1428288_at | KLF9 | Kruppel-like factor 9 |
| 1449463_at | Klk1b1 (includes others) | kallikrein 1-related pepidase b4 |
| 1419678_at | LATS2 | LATS, large tumor suppressor, homolog 2 (Drosophila) |
| 1448237_x_at | LDHB | lactate dehydrogenase B |
| 1453235_at | Lelp1 | late cornified envelope-like proline-rich 1 |
| 1436540_at | let-7 | microRNA let-7a-1 |
| 1419573_a_at | LGALS1 | lectin, galactoside-binding, soluble, 1 |
| 1418232_s_at | LIMS3L | LIM and senescent cell antigen-like domains 3-like |
| 1432180_at | LIPE | lipase, hormone-sensitive |
| 1433914_at | Lipo1 (includes others) | lipase, member O2 |
| 1433747_at | LNPEP | leucyl/cystinyl aminopeptidase |
| 1421865_at | LOC100131454 | diazepam binding inhibitor-like 5, pseudogene |
| 1438280_at | LOC100287482 | hypothetical protein LOC100287482 |
| 1448179_at | LOC100290142/USMG5 | up-regulated during skeletal muscle growth 5 homolog (mouse) |
| 1438813_at | LOC100502820 | hypothetical LOC100502820 |
| 1426803_at | LOC100502987 | hypothetical LOC100502987 |
| 1442131_at | LOC100503166 | hypothetical LOC100503166 |
| 1435952_at | LOC100503222 | hypothetical LOC100503222 |
| 1434613_at | LOC100503807 | hypothetical protein LOC100503807 |
| 1450840_a_at | LOC100503815 | 60S ribosomal protein L39-like |
| 1438944_at | LOC100504462 | hypothetical LOC100504462 |
| 1423071_x_at | LOC100505088 | hypothetical LOC100505088 |
| 1429853_at | LOC100505478 | hypothetical protein LOC100505478 |
| 1429611_at | LOC100505841/LOC728460 | zinc finger protein 474-like |
| 1432527_at | LOC100506049 | leucine-rich repeat-containing protein ENSP00000371558 |
| 1453483_at | LOC100506564 | hypothetical LOC100506564 |
| 1453293_a_at | LOC390760 | protein phosphatase inhibitor 2-like |
| 1442430_at | LOC646498 | hypothetical protein LOC646498 |
| 1431849_at | LOC646851 | hypothetical LOC646851 |
| 1431735_at | LOC730159 | hypothetical protein LOC730159 |
| 1432551_at | LOC73317 | RIKEN cDNA 1700031F10 gene |
| 1436200_at | LONRF3 | LON peptidase N-terminal domain and ring finger 3 |
| 1418723_at | LPAR3 | lysophosphatidic acid receptor 3 |
| 1430057_s_at | LRRC57 | leucine rich repeat containing 57 |
| 1453536_at | LRRC69 | leucine rich repeat containing 69 |
| 1428438_s_at | LSM14A | LSM14A, SCD6 homolog A (S. cerevisiae) |
| 1418656_at | LSM5 | LSM5 homolog, U6 small nuclear RNA associated (S. cerevisiae) |
| 1417313_at | LSM7 | LSM7 homolog, U6 small nuclear RNA associated (S. cerevisiae) |
| 1444291_at | LYSMD4 | LysM, putative peptidoglycan-binding, domain containing 4 |
| 1449892_at | LYZL1 | lysozyme-like 1 |
| 1432481_a_at | LYZL6 | lysozyme-like 6 |
| 1453280_at | MAGEB5 | melanoma antigen family B, 5 |
| 1438403_s_at | MALAT1 | metastasis associated lung adenocarcinoma transcript 1 (non-protein coding) |
| 1448647_at | MAN2A1 | mannosidase, alpha, class 2A, member 1 |
| 1438908_at | MAP3K12 | mitogen-activated protein kinase kinase kinase 12 |
| 1439323_a_at | MAP4K1 | mitogen-activated protein kinase kinase kinase kinase 1 |
| 1415922_s_at | MARCKSL1 | MARCKS-like 1 |
| 1417165_at | MBD2 | methyl-CpG binding domain protein 2 |
| 1416904_at | MBNL1 | muscleblind-like (Drosophila) |
| 1433754_at | MBNL2 | muscleblind-like 2 (Drosophila) |
| 1454840_at | MCCC2 | methylcrotonoyl-CoA carboxylase 2 (beta) |
| 1425806_a_at | MED21 | mediator complex subunit 21 |
| 1427202_at | METTL20 | methyltransferase like 20 |
| 1417710_at | METTL9 | methyltransferase like 9 |
| 1454612_at | MEX3C | mex-3 homolog C (C. elegans) |
| 1437627_at | MEX3D | mex-3 homolog D (C. elegans) |
| 1453607_at | MFAP3L | microfibrillar-associated protein 3-like |
| 1456582_x_at | MFF | mitochondrial fission factor |
| 1420911_a_at | MFGE8 | milk fat globule-EGF factor 8 protein |
| 1456987_at | MGC50722 | hypothetical MGC50722 |
| 1415897_a_at | MGST1 | microsomal glutathione S-transferase 1 |
| 1429032_at | MICALCL | MICAL C-terminal like |
| 1416335_at | MIF | macrophage migration inhibitory factor (glycosylation-inhibiting factor) |
| 1438654_x_at | MMD2 | monocyte to macrophage differentiation-associated 2 |
| 1455184_at | MOBKL1A | MOB1, Mps One Binder kinase activator-like 1A (yeast) |
| 1415778_at | MORF4L2 | mortality factor 4 like 2 |
| 1450430_at | MRC1 | mannose receptor, C type 1 |
| 1460701_a_at | MRPL52 | mitochondrial ribosomal protein L52 |
| 1423242_at | MRPS36 | mitochondrial ribosomal protein S36 |
| 1418515_at | MTF2 | metal response element binding transcription factor 2 |
| 1434278_at | MTM1 | myotubularin 1 |
| 1455238_at | MUM1L1 | melanoma associated antigen (mutated) 1-like 1 |
| 1452670_at | Myl9 | myosin, light polypeptide 9, regulatory |
| 1455052_a_at | MZT1 | mitotic spindle organizing protein 1 |
| 1449072_a_at | N6AMT2 | N-6 adenine-specific DNA methyltransferase 2 (putative) |
| 1448703_at | NAA38 | N(alpha)-acetyltransferase 38, NatC auxiliary subunit |
| 1428409_at | NAA50 | N(alpha)-acetyltransferase 50, NatE catalytic subunit |
| 1417624_at | NAB1 | NGFI-A binding protein 1 (EGR1 binding protein 1) |
| 1438678_at | NCRNA00116 | non-protein coding RNA 116 |
| 1424820_a_at | NDFIP1 | Nedd4 family interacting protein 1 |
| 1422241_a_at | NDUFA1 | NADH dehydrogenase (ubiquinone) 1 alpha subcomplex, 1, 7.5kDa |
| 1424085_at | NDUFA4 | NADH dehydrogenase (ubiquinone) 1 alpha subcomplex, 4, 9kDa |
| 1416056_a_at | NDUFB11 | NADH dehydrogenase (ubiquinone) 1 beta subcomplex, 11, 17.3kDa |
| 1420088_at | NFKBIA | nuclear factor of kappa light polypeptide gene enhancer in B-cells inhibitor, alpha |
| 1453236_at | NIPSNAP3B | nipsnap homolog 3B (C. elegans) |
| 1443817_x_at | NKX2-6 | NK2 homeobox 6 |
| 1435970_at | NLK | nemo-like kinase |
| 1423581_at | NMT2 | N-myristoyltransferase 2 |
| 1430189_at | NOL4 | nucleolar protein 4 |
| 1420487_at | NOL7 | nucleolar protein 7, 27kDa |
| 1415821_at | Nptn | neuroplastin |
| 1421516_at | NR6A1 | nuclear receptor subfamily 6, group A, member 1 |
| 1429278_at | NUBPL | nucleotide binding protein-like |
| 1444952_a_at | NUCKS1 | nuclear casein kinase and cyclin-dependent kinase substrate 1 |
| 1418566_s_at | NUDCD2 | NudC domain containing 2 |
| 1456035_at | NXF3 | nuclear RNA export factor 3 |
| 1437010_a_at | OAZ3 | ornithine decarboxylase antizyme 3 |
| 1460521_a_at | OBFC2A | oligonucleotide/oligosaccharide-binding fold containing 2A |
| 1457659_x_at | ODF1 | outer dense fiber of sperm tails 1 |
| 1450964_a_at | OSBPL9 | oxysterol binding protein-like 9 |
| 1427346_at | Ott (includes others) | ovary testis transcribed |
| 1417576_a_at | OTUB2 | OTU domain, ubiquitin aldehyde binding 2 |
| 1418272_at | OXCT2 | 3-oxoacid CoA transferase 2 |
| 1447816_x_at | OXNAD1 | oxidoreductase NAD-binding domain containing 1 |
| 1431321_at | P2RX3 | purinergic receptor P2X, ligand-gated ion channel, 3 |
| 1422849_a_at | PABPN1 | poly(A) binding protein, nuclear 1 |
| 1448493_at | PAIP2 | poly(A) binding protein interacting protein 2 |
| 1419183_at | PAPD4 | PAP associated domain containing 4 |
| 1460459_at | PAQR5 | progestin and adipoQ receptor family member V |
| 1428314_at | PCNP | PEST proteolytic signal containing nuclear protein |
| 1448433_a_at | PCOLCE | procollagen C-endopeptidase enhancer |
| 1449298_a_at | PDE1A | phosphodiesterase 1A, calmodulin-dependent |
| 1426410_at | PDK3 | pyruvate dehydrogenase kinase, isozyme 3 |
| 1416501_at | PDPK1 | 3-phosphoinositide dependent protein kinase-1 |
| 1431701_a_at | PDZK1 | PDZ domain containing 1 |
| 1438649_x_at | PEBP1 | phosphatidylethanolamine binding protein 1 |
| 1451062_a_at | PEX5L | peroxisomal biogenesis factor 5-like |
| 1454888_at | PFDN4 | prefoldin subunit 4 |
| 1453962_at | PFN3 | profilin 3 |
| 1424615_at | Pgap2 | post-GPI attachment to proteins 2 |
| 1423451_at | PGRMC1 | progesterone receptor membrane component 1 |
| 1456606_a_at | PHACTR1 | phosphatase and actin regulator 1 |
| 1417053_at | PHB | prohibitin |
| 1453271_at | PHF14 | PHD finger protein 14 |
| 1426752_at | PHF17 | PHD finger protein 17 |
| 1418960_at | PHF20L1 | PHD finger protein 20-like 1 |
| 1437621_x_at | PHGDH | phosphoglycerate dehydrogenase |
| 1429004_at | PHIP | pleckstrin homology domain interacting protein |
| 1441870_s_at | PKD2 (includes EG:18764) | polycystic kidney disease 2 (autosomal dominant) |
| 1421138_a_at | PKIB | protein kinase (cAMP-dependent, catalytic) inhibitor beta |
| 1430700_a_at | PLA2G7 | phospholipase A2, group VII (platelet-activating factor acetylhydrolase, plasma) |
| 1448786_at | PLBD1 | phospholipase B domain containing 1 |
| 1426926_at | PLCG2 | phospholipase C, gamma 2 (phosphatidylinositol-specific) |
| 1432460_at | Pldi | polymorphic derived intron containing |
| 1420839_at | PLEKHA3 | pleckstrin homology domain containing, family A (phosphoinositide binding specific) member 3 |
| 1417768_at | PNPLA8 | patatin-like phospholipase domain containing 8 |
| 1452596_at | POLR2K | polymerase (RNA) II (DNA directed) polypeptide K, 7.0kDa |
| 1429514_at | PPAP2B | phosphatidic acid phosphatase type 2B |
| 1428345_at | PPAPDC2 | phosphatidic acid phosphatase type 2 domain containing 2 |
| 1435766_at | PPHLN1 | periphilin 1 |
| 1437649_x_at | PPIB | peptidylprolyl isomerase B (cyclophilin B) |
| 1460165_at | PPP1CA | protein phosphatase 1, catalytic subunit, alpha isozyme |
| 1456462_x_at | PPP1CB | protein phosphatase 1, catalytic subunit, beta isozyme |
| 1440285_at | Ppp1r9a | protein phosphatase 1, regulatory (inhibitor) subunit 9A |
| 1426621_a_at | PPP2R2B | protein phosphatase 2, regulatory subunit B, beta |
| 1422467_at | PPT1 | palmitoyl-protein thioesterase 1 |
| 1449962_at | Pramel3 (includes others) | preferentially expressed antigen in melanoma-like 3 |
| 1416292_at | PRDX3 | peroxiredoxin 3 |
| 1416166_a_at | PRDX4 | peroxiredoxin 4 |
| 1448202_x_at | PRELID1 | PRELI domain containing 1 |
| 1451576_at | PRKDC | protein kinase, DNA-activated, catalytic polypeptide |
| 1439379_x_at | PRM1 | protamine 1 |
| 1448105_at | PRM2 | protamine 2 |
| 1422913_at | PRM3 | protamine 3 |
| 1448233_at | PRNP | prion protein |
| 1449427_at | PRSS37 | protease, serine, 37 |
| 1429578_at | Prss52 | protease, serine, 52 |
| 1420278_at | PRSS58 | protease, serine, 58 |
| 1460198_a_at | PSMB3 | proteasome (prosome, macropain) subunit, beta type, 3 |
| 1417189_at | PSME2 | proteasome (prosome, macropain) activator subunit 2 (PA28 beta) |
| 1418015_at | PUM2 | pumilio homolog 2 (Drosophila) |
| 1420628_at | PURA | purine-rich element binding protein A |
| 1452324_at | PVT1 | Pvt1 oncogene (non-protein coding) |
| 1456213_x_at | QARS | glutaminyl-tRNA synthetase |
| 1417073_a_at | QKI | quaking homolog, KH domain RNA binding (mouse) |
| 1426622_a_at | QPCT | glutaminyl-peptide cyclotransferase |
| 1416426_at | RAB5A | RAB5A, member RAS oncogene family |
| 1448391_at | RAB9A | RAB9A, member RAS oncogene family |
| 1416161_at | RAD21 | RAD21 homolog (S. pombe) |
| 1451092_a_at | Rangap1 | RAN GTPase activating protein 1 |
| 1424139_at | RAP1A | RAP1A, member of RAS oncogene family |
| 1435519_at | RAP1B | RAP1B, member of RAS oncogene family |
| 1415775_at | RBBP7 | retinoblastoma binding protein 7 |
| 1426419_at | RBM26 | RNA binding motif protein 26 |
| 1422660_at | RBM3 | RNA binding motif (RNP1, RRM) protein 3 |
| 1426671_a_at | RBM39 | RNA binding motif protein 39 |
| 1451237_s_at | RBM7 | RNA binding motif protein 7 |
| 1420786_a_at | Rbmy1a1 (includes others) | RNA binding motif protein, Y chromosome, family 1, member A1 |
| 1432232_at | RCOR3 | REST corepressor 3 |
| 1433509_s_at | REEP1 | receptor accessory protein 1 |
| 1430128_a_at | REEP6 | receptor accessory protein 6 |
| 1424632_a_at | REV3L | REV3-like, catalytic subunit of DNA polymerase zeta (yeast) |
| 1418691_at | RGS9 | regulator of G-protein signaling 9 |
| 1449914_at | RIBC1 | RIB43A domain with coiled-coils 1 |
| 1429982_at | RIMKLB | ribosomal modification protein rimK-like family member B |
| 1452734_at | RNASET2 | ribonuclease T2 |
| 1443830_x_at | RNF103 | ring finger protein 103 |
| 1426404_a_at | RNF11 | ring finger protein 11 |
| 1429265_a_at | RNF130 | ring finger protein 130 |
| 1456802_at | RNF133 | ring finger protein 133 |
| 1419368_a_at | RNF138 | ring finger protein 138 |
| 1430102_at | RNF148 | ring finger protein 148 |
| 1429321_at | RNF149 | ring finger protein 149 |
| 1454753_at | RNPEPL1 | arginyl aminopeptidase (aminopeptidase B)-like 1 |
| 1450994_at | ROCK1 | Rho-associated, coiled-coil containing protein kinase 1 |
| 1422543_at | ROPN1 | rhophilin associated tail protein 1 |
| 1448938_at | RPA3 | replication protein A3, 14kDa |
| 1421945_a_at | RPF2 | ribosome production factor 2 homolog (S. cerevisiae) |
| 1448157_s_at | RPL10 (includes others) | ribosomal protein L10 |
| 1438626_x_at | RPL14 | ribosomal protein L14 |
| 1437729_at | RPL27A | ribosomal protein L27a |
| 1438655_a_at | RPL34 | ribosomal protein L34 |
| 1416807_at | RPL36A/hCG 1787519 | ribosomal protein L36a |
| 1460543_x_at | RPL37A | ribosomal protein L37a |
| 1433472_x_at | Rpl38 (includes others) | ribosomal protein L38 |
| 1455578_x_at | RPL41 | ribosomal protein L41 |
| 1434358_x_at | RPS21 | ribosomal protein S21 |
| 1430978_at | RPS25 | ribosomal protein S25 |
| 1415716_a_at | RPS27 | ribosomal protein S27 |
| 1451101_a_at | Rps28 (includes others) | ribosomal protein S28 |
| 1438859_x_at | RPS29 | ribosomal protein S29 |
| 1416276_a_at | RPS4X | ribosomal protein S4, X-linked |
| 1435816_at | RPS6 | ribosomal protein S6 |
| 1434624_x_at | RPS9 | ribosomal protein S9 |
| 1417398_at | RRAS2 | related RAS viral (r-ras) oncogene homolog 2 |
| 1435284_at | RTN4 | reticulon 4 |
| 1457983_s_at | RWDD4 | RWD domain containing 4 |
| 1456642_x_at | S100A10 | S100 calcium binding protein A10 |
| 1460351_at | S100A11 | S100 calcium binding protein A11 |
| 1451204_at | SCARA5 | scavenger receptor class A, member 5 (putative) |
| 1415822_at | Scd2 | stearoyl-Coenzyme A desaturase 2 |
| 1449686_s_at | SCP2 | sterol carrier protein 2 |
| 1460698_a_at | SEC11C | SEC11 homolog C (S. cerevisiae) |
| 1423225_at | SELK | selenoprotein K |
| 1435602_at | SEPHS2 | selenophosphate synthetase 2 |
| 1452141_a_at | SEPP1 | selenoprotein P, plasma, 1 |
| 1448108_at | SERINC1 | serine incorporator 1 |
| 1421556_at | Serpina3a (includes others) | serine (or cysteine) peptidase inhibitor, clade A, member 3A |
| 1418813_at | SERPINA5 | serpin peptidase inhibitor, clade A (alpha-1 antiproteinase, antitrypsin), member 5 |
| 1436681_x_at | SF3B14 | splicing factor 3B, 14 kDa subunit |
| 1436997_x_at | SH3BGRL | SH3 domain binding glutamic acid-rich protein like |
| 1421922_at | SH3BP5 | SH3-domain binding protein 5 (BTK-associated) |
| 1431592_a_at | SH3KBP1 | SH3-domain kinase binding protein 1 |
| 1418574_a_at | SHFM1 | split hand/foot malformation (ectrodactyly) type 1 |
| 1420106_at | SIAH1 | seven in absentia homolog 1 (Drosophila) |
| 1449293_a_at | SKP2 | S-phase kinase-associated protein 2 (p45) |
| 1417622_at | SLC12A2 | solute carrier family 12 (sodium/potassium/chloride transporters), member 2 |
| 1424562_a_at | SLC25A4 | solute carrier family 25 (mitochondrial carrier; adenine nucleotide translocator), member 4 |
| 1436874_x_at | SLC25A6 | solute carrier family 25 (mitochondrial carrier; adenine nucleotide translocator), member 6 |
| 1436279_at | SLC26A7 | solute carrier family 26, member 7 |
| 1418843_at | SLC30A4 | solute carrier family 30 (zinc transporter), member 4 |
| 1426722_at | SLC38A2 | solute carrier family 38, member 2 |
| 1449791_x_at | SLC38A9 | solute carrier family 38, member 9 |
| 1422487_at | SMAD4 | SMAD family member 4 |
| 1432029_a_at | SMAP1 | small ArfGAP 1 |
| 1418358_at | SMCP | sperm mitochondria-associated cysteine-rich protein |
| 1418095_at | SMPX | small muscle protein, X-linked |
| 1436506_a_at | Snhg6 | small nucleolar RNA host gene (non-protein coding) 6 |
| 1447896_s_at | Snhg8 | small nucleolar RNA host gene 8 |
| 1425319_s_at | SNRNP48 | small nuclear ribonucleoprotein 48kDa (U11/U12) |
| 1428672_at | SNRPF | small nuclear ribonucleoprotein polypeptide F |
| 1448358_s_at | SNRPG | small nuclear ribonucleoprotein polypeptide G |
| 1420766_at | SOCS7 | suppressor of cytokine signaling 7 |
| 1451124_at | SOD1 | superoxide dismutase 1, soluble |
| 1440827_x_at | SOX5 | SRY (sex determining region Y)-box 5 |
| 1429571_a_at | SPACA1 | sperm acrosome associated 1 |
| 1429757_at | SPACA7 | sperm acrosome associated 7 |
| 1419432_at | Spam1 | sperm adhesion molecule 1 |
| 1448392_at | SPARC | secreted protein, acidic, cysteine-rich (osteonectin) |
| 1430351_at | SPATA18 | spermatogenesis associated 18 homolog (rat) |
| 1430023_at | SPATA24 | spermatogenesis associated 24 |
| 1453335_a_at | SPATA3 | spermatogenesis associated 3 |
| 1429864_at | SPATC1 | spermatogenesis and centriole associated 1 |
| 1450907_at | SPCS2 | signal peptidase complex subunit 2 homolog (S. cerevisiae) |
| 1436809_a_at | SPIN1 | spindlin 1 |
| 1420588_at | SPINLW1 | serine peptidase inhibitor-like, with Kunitz and WAP domains 1 (eppin) |
| 1438968_x_at | SPINT2 | serine peptidase inhibitor, Kunitz type, 2 |
| 1427134_at | SREK1 | splicing regulatory glutamine/lysine-rich protein 1 |
| 1417426_at | SRGN | serglycin |
| 1447053_x_at | SSR3 | signal sequence receptor, gamma (translocon-associated protein gamma) |
| 1448524_s_at | SSR4 | signal sequence receptor, delta (translocon-associated protein delta) |
| 1449912_at | SSX5 | synovial sarcoma, X breakpoint 5 |
| 1417616_at | ST6GALNAC2 | ST6 (alpha-N-acetyl-neuraminyl-2,3-beta-galactosyl-1,3)-N-acetylgalactosaminide alpha-2,6-sialyltransferase 2 |
| 1453584_at | STK36 | serine/threonine kinase 36 |
| 1423535_at | STRN3 | striatin, calmodulin binding protein 3 |
| 1434412_x_at | STUB1 | STIP1 homology and U-box containing protein 1, E3 ubiquitin protein ligase |
| 1422693_a_at | SUB1 | SUB1 homolog (S. cerevisiae) |
| 1420447_at | SULT1E1 | sulfotransferase family 1E, estrogen-preferring, member 1 |
| 1429912_at | SUN5 | Sad1 and UNC84 domain containing 5 |
| 1422979_at | SUV39H2 | suppressor of variegation 3-9 homolog 2 (Drosophila) |
| 1416472_at | SYAP1 | synapse associated protein 1 |
| 1429270_a_at | SYCE2 | synaptonemal complex central element protein 2 |
| 1427291_at | SYCP1 | synaptonemal complex protein 1 |
| 1422881_s_at | SYPL1 | synaptophysin-like 1 |
| 1438206_a_at | Sys1 | SYS1 Golgi-localized integral membrane protein homolog (S. cerevisiae) |
| 1435303_at | TAF4B | TAF4b RNA polymerase II, TATA box binding protein (TBP)-associated factor, 105kDa |
| 1428467_at | TARDBP | TAR DNA binding protein |
| 1419257_at | TCEA1 | transcription elongation factor A (SII), 1 |
| 1418171_at | TCEAL8 | transcription elongation factor A (SII)-like 8 |
| 1421682_a_at | TCTE3 | t-complex-associated-testis-expressed 3 |
| 1449831_at | TCTEX1D1 | Tctex1 domain containing 1 |
| 1432536_at | TEPP | testis, prostate and placenta expressed |
| 1421183_at | TEX12 | testis expressed 12 |
| 1441893_at | Tex24 | testis expressed gene 24 |
| 1448224_at | TFAM | transcription factor A, mitochondrial |
| 1427968_at | TGIF2LX | TGFB-induced factor homeobox 2-like, X-linked |
| 1454677_at | TIMP2 | TIMP metallopeptidase inhibitor 2 |
| 1426612_at | TIPIN | TIMELESS interacting protein |
| 1449554_at | TLE3 | transducin-like enhancer of split 3 (E(sp1) homolog, Drosophila) |
| 1440820_x_at | TMCO2 | transmembrane and coiled-coil domains 2 |
| 1432203_at | Tmco5b | transmembrane and coiled-coil domains 5B |
| 1455968_x_at | TMED2 | transmembrane emp24 domain trafficking protein 2 |
| 1419918_at | TMED7 | transmembrane emp24 protein transport domain containing 7 |
| 1426649_at | TMEFF1 | transmembrane protein with EGF-like and two follistatin-like domains 1 |
| 1416479_a_at | TMEM14C | transmembrane protein 14C |
| 1438561_x_at | TMEM180 | transmembrane protein 180 |
| 1424477_at | TMEM184A | transmembrane protein 184A |
| 1426628_at | TMEM184C | transmembrane protein 184C |
| 1451652_a_at | TMEM188 | transmembrane protein 188 |
| 1432550_at | TMEM190 | transmembrane protein 190 |
| 1430421_a_at | TMEM205 | transmembrane protein 205 |
| 1429825_at | TMEM225 | transmembrane protein 225 |
| 1438369_x_at | TMEM85 | transmembrane protein 85 |
| 1415906_at | TMSB10/TMSB4X | thymosin beta 4, X-linked |
| 1438632_x_at | TNP1 | transition protein 1 (during histone to protamine replacement) |
| 1422419_s_at | TNP2 | transition protein 2 (during histone to protamine replacement) |
| 1455357_x_at | TOMM20 | translocase of outer mitochondrial membrane 20 homolog (yeast) |
| 1426084_a_at | TOR1AIP1 | torsin A interacting protein 1 |
| 1438547_x_at | TOR2A | torsin family 2, member A |
| 1430106_at | TP53TG5 | TP53 target 5 |
| 1429276_at | TRANK1 | tetratricopeptide repeat and ankyrin repeat containing 1 |
| 1432158_a_at | TRAPPC2 | trafficking protein particle complex 2 |
| 1427259_at | TRIM24 | tripartite motif containing 24 |
| 1419440_at | TRIM54 | tripartite motif containing 54 |
| 1427407_s_at | TRIP11 | thyroid hormone receptor interactor 11 |
| 1420587_at | TSGA13 | testis specific, 13 |
| 1420552_s_at | Tsga8 | testis specific gene A8 |
| 1448501_at | TSPAN6 | tetraspanin 6 |
| 1418557_s_at | TSSK3 | testis-specific serine kinase 3 |
| 1418956_at | TSSK6 | testis-specific serine kinase 6 |
| 1420439_at | Tsx | testis specific X-linked gene |
| 1437878_s_at | TTC14 | tetratricopeptide repeat domain 14 |
| 1442763_s_at | TTLL10 | tubulin tyrosine ligase-like family, member 10 |
| 1418884_x_at | TUBA1A | tubulin, alpha 1a |
| 1417373_a_at | TUBA4A | tubulin, alpha 4a |
| 1416119_at | TXN (includes EG:116484) | thioredoxin |
| 1437266_at | TXNDC2 | thioredoxin domain containing 2 (spermatozoa) |
| 1420333_at | TXNDC8 | thioredoxin domain containing 8 (spermatozoa) |
| 1448116_at | UBA1 | ubiquitin-like modifier activating enzyme 1 |
| 1435643_x_at | Ubb | ubiquitin B |
| 1448356_at | UBE2D2 | ubiquitin-conjugating enzyme E2D 2 |
| 1415688_at | UBE2G1 | ubiquitin-conjugating enzyme E2G 1 |
| 1417033_at | UBE2G2 | ubiquitin-conjugating enzyme E2G 2 |
| 1417983_a_at | UBE2V2 | ubiquitin-conjugating enzyme E2 variant 2 |
| 1452054_at | UBE2W | ubiquitin-conjugating enzyme E2W (putative) |
| 1460272_at | UBL4B | ubiquitin-like 4B |
| 1437955_at | UBQLNL | ubiquilin-like |
| 1433479_at | UBR7 | ubiquitin protein ligase E3 component n-recognin 7 (putative) |
| 1448260_at | UCHL1 | ubiquitin carboxyl-terminal esterase L1 (ubiquitin thiolesterase) |
| 1434485_a_at | UGP2 | UDP-glucose pyrophosphorylase 2 |
| 1455997_a_at | UQCRB | ubiquinol-cytochrome c reductase binding protein |
| 1425805_a_at | USP12 | ubiquitin specific peptidase 12 |
| 1453670_at | USP50 | ubiquitin specific peptidase 50 |
| 1421750_a_at | VBP1 | von Hippel-Lindau binding protein 1 |
| 1456607_at | VCPIP1 | valosin containing protein (p97)/p47 complex interacting protein 1 |
| 1429085_at | VEZF1 | vascular endothelial zinc finger 1 |
| 1438118_x_at | VIM | vimentin |
| 1452770_at | VKORC1 | vitamin K epoxide reductase complex, subunit 1 |
| 1419177_at | VPS37A | vacuolar protein sorting 37 homolog A (S. cerevisiae) |
| 1449095_at | VPS54 | vacuolar protein sorting 54 homolog (S. cerevisiae) |
| 1456129_at | VWA3A | von Willebrand factor A domain containing 3A |
| 1434778_at | WAPAL | wings apart-like homolog (Drosophila) |
| 1451230_a_at | WBP5 | WW domain binding protein 5 |
| 1430644_at | Wbscr25 | Williams Beuren syndrome chromosome region 25 (human) |
| 1430082_at | WDR64 | WD repeat domain 64 |
| 1457766_at | Wfdc6a | WAP four-disulfide core domain 6A |
| 1448145_at | WWP2 | WW domain containing E3 ubiquitin protein ligase 2 |
| 1436981_a_at | YWHAZ | tyrosine 3-monooxygenase/tryptophan 5-monooxygenase activation protein, zeta polypeptide |
| 1436067_at | ZBTB10 | zinc finger and BTB domain containing 10 |
| 1417321_at | ZCCHC7 | zinc finger, CCHC domain containing 7 |
| 1456783_at | ZDBF2 | zinc finger, DBF-type containing 2 |
| 1438607_at | ZDHHC19 | zinc finger, DHHC-type containing 19 |
| 1454975_at | ZFC3H1 | zinc finger, C3H1-type containing |
| 1438050_x_at | Zfp640 (includes others) | zinc finger protein 640 |
| 1449978_at | Zfy1/Zfy2 | zinc finger protein 2, Y linked |
| 1438237_at | ZNF600/ZNF888 | zinc finger protein 600 |
| 1426679_at | ZNF706 | zinc finger protein 706 |
| 1432750_at | ZNF711 | zinc finger protein 711 |
