# Supplementary material for: Role of Gonadotropin Regulated Testicular RNA Helicase (GRTH/DDX25) on Polysomal Associated mRNAs in Mouse Testis
Source: PLoS One. 2012 Mar 30;7(3):e32470. doi: 10.1371/journal.pone.0032470 (PMC3316541; doi:10.1371/journal.pone.0032470)
Supplement: Table S3 — Differentially regulated genes associated with GRTH in testicular polysomes of wild type adult mice. A. List of differentially down-regulated genes (186) associated with GRTH in polysomes. B. List of differentially up-regulated genes (7) associated with GRTH in polysomes. (DOCX) [file pone.0032470.s007.docx]

| **Table S3. Differentially regulated genes associated with GRTH in testicular polysomes of wild type adult mice.**  **A. List of differentially down-regulated genes (186) associated with GRTH in testicular polysomes of wild type adult mice** | | | | | | |  |  |
| --- | --- | --- | --- | --- | --- | --- | --- | --- |
|  |  |  |  |  |  |  |  |  |
| **Affymetrix ID** | **Symbol** | **Entrez Gene Name** |  |  |  |  |  |  |
| 1452602_a_at | 1700001C19Rik | RIKEN cDNA 1700001C19 gene |  |  |  |  |  |  |
| 1422722_at | 1700001K19Rik | RIKEN cDNA 1700001K19 gene |  |  |  |  |  |  |
| 1437988_x_at | 1700003E24Rik | RIKEN cDNA 1700003E24 gene |  |  |  |  |  |  |
| 1431103_at | 1700003P14Rik | RIKEN cDNA 1700003P14 gene |  |  |  |  |  |  |
| 1430284_at | 1700008P02Rik | RIKEN cDNA 1700008P02 gene |  |  |  |  |  |  |
| 1432505_at | 1700009J07Rik | RIKEN cDNA 1700009J07 gene |  |  |  |  |  |  |
| 1429747_at | 1700009N14Rik | RIKEN cDNA 1700009N14 gene |  |  |  |  |  |  |
| 1429636_at | 1700010D01Rik (includes others) | predicted gene 9043 |  |  |  |  |  |  |
| 1429142_at | 1700012A03Rik | RIKEN cDNA 1700012A03 gene |  |  |  |  |  |  |
| 1418303_at | 1700012L04Rik (includes others) | predicted gene 14475 |  |  |  |  |  |  |
| 1459995_at | 1700015G11Rik | RIKEN cDNA 1700015G11 gene |  |  |  |  |  |  |
| 1438791_at | 1700016P04Rik | RIKEN cDNA 1700016P04 gene |  |  |  |  |  |  |
| 1429513_at | 1700019M22Rik/RP23-438H3.2 | RIKEN cDNA 1700019M22 gene |  |  |  |  |  |  |
| 1447710_at | 1700019P21Rik | RIKEN cDNA 1700019P21 gene |  |  |  |  |  |  |
| 1430524_at | 1700020N15Rik/Gm6812 | RIKEN cDNA 1700020N15 gene |  |  |  |  |  |  |
| 1453383_at | 1700022F17Rik | RIKEN cDNA 1700022F17 gene |  |  |  |  |  |  |
| 1429855_at | 1700023I07Rik | protein phosphatase 1, regulatory (inhibitor) subunit 2 pseudogene | | |  |  |  |  |
| 1429828_at | 1700024P04Rik | RIKEN cDNA 1700024P04 gene |  |  |  |  |  |  |
| 1458398_at | 1700027F06Rik | RIKEN cDNA 1700027F06 gene |  |  |  |  |  |  |
| 1450999_a_at | 1700029H14Rik | RIKEN cDNA 1700029H14 gene |  |  |  |  |  |  |
| 1457057_at | 1700042G07Rik | RIKEN cDNA 1700042G07 gene |  |  |  |  |  |  |
| 1438384_at | 1700047L15Rik | SPANX family, member N4 |  |  |  |  |  |  |
| 1440768_x_at | 1700052I22Rik | RIKEN cDNA 1700052I22 gene |  |  |  |  |  |  |
| 1457421_at | 1700063D05Rik | RIKEN cDNA 1700063D05 gene |  |  |  |  |  |  |
| 1419337_at | 1700080E11Rik | RIKEN cDNA 1700080E11 gene |  |  |  |  |  |  |
| 1453447_at | 1700109H08Rik | RIKEN cDNA 1700109H08 gene |  |  |  |  |  |  |
| 1457050_at | 1700129O19Rik | RIKEN cDNA 1700129O19 gene |  |  |  |  |  |  |
| 1419718_at | 4921530L21Rik | RIKEN cDNA 4921530L21 gene |  |  |  |  |  |  |
| 1445661_at | 4930406D18Rik | RIKEN cDNA 4930406D18 gene |  |  |  |  |  |  |
| 1430872_at | 4930412O13Rik | RIKEN cDNA 4930412O13 gene |  |  |  |  |  |  |
| 1453838_at | 4930471G03Rik | RIKEN cDNA 4930471G03 gene |  |  |  |  |  |  |
| 1431184_a_at | 4930503B20Rik | RIKEN cDNA 4930503B20 gene |  |  |  |  |  |  |
| 1454133_s_at | 4930523O13Rik | RIKEN cDNA 4930523O13 gene |  |  |  |  |  |  |
| 1421367_at | 4930549C01Rik | RIKEN cDNA 4930549C01 gene |  |  |  |  |  |  |
| 1431673_at | 4930557A04Rik | RIKEN cDNA 4930557A04 gene |  |  |  |  |  |  |
| 1431855_at | 4930570E03Rik | RIKEN cDNA 4930570E03 gene |  |  |  |  |  |  |
| 1430063_at | 4930571K23Rik | RIKEN cDNA 4930571K23 gene |  |  |  |  |  |  |
| 1429551_at | 4930579G22Rik | RIKEN cDNA 4930579G22 gene |  |  |  |  |  |  |
| 1429840_at | 4933439G12Rik | RIKEN cDNA 4933439G12 gene |  |  |  |  |  |  |
| 1439000_at | ACSBG2 | acyl-CoA synthetase bubblegum family member 2 |  |  |  |  |  |  |
| 1422526_at | ACSL1 | acyl-CoA synthetase long-chain family member 1 |  |  |  |  |  |  |
| 1439649_at | ADC | arginine decarboxylase |  |  |  |  |  |  |
| 1429609_at | ADORA3 | adenosine A3 receptor |  |  |  |  |  |  |
| 1428821_at | AGPAT2 | 1-acylglycerol-3-phosphate O-acyltransferase 2 (lysophosphatidic acid acyltransferase, beta) | | | | |  |  |
| 1418204_s_at | AIF1 | allograft inflammatory factor 1 |  |  |  |  |  |  |
| 1418279_a_at | AKAP1 | A kinase (PRKA) anchor protein 1 |  |  |  |  |  |  |
| 1456589_x_at | ALLC | allantoicase |  |  |  |  |  |  |
| 1420468_at | ASB17 | ankyrin repeat and SOCS box containing 17 |  |  |  |  |  |  |
| 1426309_at | ASB9 | ankyrin repeat and SOCS box containing 9 |  |  |  |  |  |  |
| 1451376_at | ATL3 | atlastin GTPase 3 |  |  |  |  |  |  |
| 1439446_at | BC048507 | cDNA sequence BC048507 |  |  |  |  |  |  |
| 1450846_at | BZW1 | basic leucine zipper and W2 domains 1 |  |  |  |  |  |  |
| 1452802_at | C10orf62 | chromosome 10 open reading frame 62 |  |  |  |  |  |  |
| 1430314_at | C14orf148 | chromosome 14 open reading frame 148 |  |  |  |  |  |  |
| 1430121_at | C17orf105 | chromosome 17 open reading frame 105 |  |  |  |  |  |  |
| 1430674_at | C1orf100 | chromosome 1 open reading frame 100 |  |  |  |  |  |  |
| 1430422_at | C20orf141 | chromosome 20 open reading frame 141 |  |  |  |  |  |  |
| 1432308_at | C20orf71 | chromosome 20 open reading frame 71 |  |  |  |  |  |  |
| 1430892_at | C2orf51 | chromosome 2 open reading frame 51 |  |  |  |  |  |  |
| 1432404_at | C2orf73 | chromosome 2 open reading frame 73 |  |  |  |  |  |  |
| 1430368_s_at | C2orf88 | chromosome 2 open reading frame 88 |  |  |  |  |  |  |
| 1453879_at | C4orf36 | chromosome 4 open reading frame 36 |  |  |  |  |  |  |
| 1441883_at | C5orf32 | chromosome 5 open reading frame 32 |  |  |  |  |  |  |
| 1429989_at | C5orf50 | chromosome 5 open reading frame 50 |  |  |  |  |  |  |
| 1453142_at | C7orf31 | chromosome 7 open reading frame 31 |  |  |  |  |  |  |
| 1424795_a_at | C9orf50 | chromosome 9 open reading frame 50 |  |  |  |  |  |  |
| 1429744_at | CABS1 | calcium-binding protein, spermatid-specific 1 |  |  |  |  |  |  |
| 1453233_s_at | CALR3 | calreticulin 3 |  |  |  |  |  |  |
| 1418556_at | CAPZA3 | capping protein (actin filament) muscle Z-line, alpha 3 |  |  |  |  |  |  |
| 1451166_a_at | CCDC101 | coiled-coil domain containing 101 |  |  |  |  |  |  |
| 1428965_at | CCDC54 | coiled-coil domain containing 54 |  |  |  |  |  |  |
| 1429852_at | CCDC57 | coiled-coil domain containing 57 |  |  |  |  |  |  |
| 1449487_at | CCDC70 | coiled-coil domain containing 70 |  |  |  |  |  |  |
| 1418459_at | CCDC91 | coiled-coil domain containing 91 |  |  |  |  |  |  |
| 1448983_at | CDRT4 | CMT1A duplicated region transcript 4 |  |  |  |  |  |  |
| 1451100_a_at | CDV3 | CDV3 homolog (mouse) |  |  |  |  |  |  |
| 1448935_at | CDYL | chromodomain protein, Y-like |  |  |  |  |  |  |
| 1432522_s_at | CEP112 | centrosomal protein 112kDa |  |  |  |  |  |  |
| 1451092_a_at | CHADL | chondroadherin-like |  |  |  |  |  |  |
| 1417795_at | CHL1 | cell adhesion molecule with homology to L1CAM (close homolog of L1) | | |  |  |  |  |
| 1428574_a_at | Chn2 | chimerin (chimaerin) 2 |  |  |  |  |  |  |
| 1425321_a_at | CLMN | calmin (calponin-like, transmembrane) |  |  |  |  |  |  |
| 1416541_at | CLPB | ClpB caseinolytic peptidase B homolog (E. coli) |  |  |  |  |  |  |
| 1430855_at | COL20A1 | collagen, type XX, alpha 1 |  |  |  |  |  |  |
| 1445868_at | CPEB3 | cytoplasmic polyadenylation element binding protein 3 |  |  |  |  |  |  |
| 1423370_a_at | CSNK1G2 | casein kinase 1, gamma 2 |  |  |  |  |  |  |
| 1429424_at | Cst13 | cystatin 13 |  |  |  |  |  |  |
| 1424479_at | CST8 | cystatin 8 (cystatin-related epididymal specific) |  |  |  |  |  |  |
| 1420424_at | CXorf27 | chromosome X open reading frame 27 |  |  |  |  |  |  |
| 1456192_x_at | Cypt1 (includes others) | cysteine-rich perinuclear theca 1 |  |  |  |  |  |  |
| 1429374_at | Cypt12 | cysteine-rich perinuclear theca 12 |  |  |  |  |  |  |
| 1452079_s_at | DCUN1D1 | DCN1, defective in cullin neddylation 1, domain containing 1 (S. cerevisiae) | | |  |  |  |  |
| 1418263_at | DDX25 | DEAD (Asp-Glu-Ala-Asp) box polypeptide 25 |  |  |  |  |  |  |
| 1450991_at | DNAJB7 | DnaJ (Hsp40) homolog, subfamily B, member 7 |  |  |  |  |  |  |
| 1418725_at | DNAJC5B | DnaJ (Hsp40) homolog, subfamily C, member 5 beta |  |  |  |  |  |  |
| 1438789_s_at | DPYSL3 | dihydropyrimidinase-like 3 |  |  |  |  |  |  |
| 1453400_at | DYDC2 | DPY30 domain containing 2 |  |  |  |  |  |  |
| 1439439_x_at | EEF1D | eukaryotic translation elongation factor 1 delta (guanine nucleotide exchange protein) | | | |  |  |  |
| 1444010_at | EIF4E | eukaryotic translation initiation factor 4E |  |  |  |  |  |  |
| 1431788_at | FABP12 | fatty acid binding protein 12 |  |  |  |  |  |  |
| 1453026_at | FAM166A | family with sequence similarity 166, member A |  |  |  |  |  |  |
| 1429397_a_at | FAM71D | family with sequence similarity 71, member D |  |  |  |  |  |  |
| 1449332_at | FHL5 | four and a half LIM domains 5 |  |  |  |  |  |  |
| 1418948_at | FSCN3 | fascin homolog 3, actin-bundling protein, testicular (Strongylocentrotus purpuratus) | | | |  |  |  |
| 1419016_at | FUNDC2 | FUN14 domain containing 2 |  |  |  |  |  |  |
| 1449974_at | GAPDHS | glyceraldehyde-3-phosphate dehydrogenase, spermatogenic | |  |  |  |  |  |
| 1437679_a_at | GLRX2 | glutaredoxin 2 |  |  |  |  |  |  |
| 1426235_a_at | GLUL | glutamate-ammonia ligase |  |  |  |  |  |  |
| 1439149_s_at | Gm9999 | predicted gene 9999 |  |  |  |  |  |  |
| 1430181_at | H1FNT | H1 histone family, member N, testis-specific |  |  |  |  |  |  |
| 1415982_at | HERPUD2 | HERPUD family member 2 |  |  |  |  |  |  |
| 1448512_at | HILS1 | histone linker H1 domain, spermatid-specific 1 |  |  |  |  |  |  |
| 1429349_at | HMGB4 | high mobility group box 4 |  |  |  |  |  |  |
| 1419625_at | HSPA1L | heat shock 70kDa protein 1-like |  |  |  |  |  |  |
| 1458481_at | IL2RG | interleukin 2 receptor, gamma |  |  |  |  |  |  |
| 1438990_x_at | Iqcf3 | IQ motif containing F3 |  |  |  |  |  |  |
| 1429666_at | KCTD16 | potassium channel tetramerisation domain containing 16 | |  |  |  |  |  |
| 1449463_at | Klk1b1 (includes others) | kallikrein 1-related pepidase b4 |  |  |  |  |  |  |
| 1453235_at | Lelp1 | late cornified envelope-like proline-rich 1 |  |  |  |  |  |  |
| 1432180_at | LIPE | lipase, hormone-sensitive |  |  |  |  |  |  |
| 1421865_at | LOC100131454 | diazepam binding inhibitor-like 5, pseudogene |  |  |  |  |  |  |
| 1438280_at | LOC100287482 | hypothetical protein LOC100287482 |  |  |  |  |  |  |
| 1438813_at | LOC100502820 | hypothetical LOC100502820 |  |  |  |  |  |  |
| 1442131_at | LOC100503166 | hypothetical LOC100503166 |  |  |  |  |  |  |
| 1438944_at | LOC100504462 | hypothetical LOC100504462 |  |  |  |  |  |  |
| 1429853_at | LOC100505478 | hypothetical protein LOC100505478 |  |  |  |  |  |  |
| 1429611_at | LOC100505841/LOC728460 | zinc finger protein 474-like |  |  |  |  |  |  |
| 1453483_at | LOC100506564 | hypothetical LOC100506564 |  |  |  |  |  |  |
| 1459803_x_at | LOC390760 | protein phosphatase inhibitor 2-like |  |  |  |  |  |  |
| 1431849_at | LOC646851 | hypothetical LOC646851 |  |  |  |  |  |  |
| 1432551_at | LOC73317 | RIKEN cDNA 1700031F10 gene |  |  |  |  |  |  |
| 1430057_s_at | LRRC57 | leucine rich repeat containing 57 |  |  |  |  |  |  |
| 1453536_at | LRRC69 | leucine rich repeat containing 69 |  |  |  |  |  |  |
| 1425803_a_at | MBD2 | Methyl-CpG binding |  |  |  |  |  |  |
| 1453607_at | MFAP3L | microfibrillar-associated protein 3-like |  |  |  |  |  |  |
| 1456987_at | MGC50722 | hypothetical MGC50722 |  |  |  |  |  |  |
| 1430189_at | NOL4 | nucleolar protein 4 |  |  |  |  |  |  |
| 1437010_a_at | OAZ3 | ornithine decarboxylase antizyme 3 |  |  |  |  |  |  |
| 1440821_x_at | ODF1 | outer dense fiber of sperm tails 1 |  |  |  |  |  |  |
| 1417576_a_at | OTUB2 | OTU domain, ubiquitin aldehyde binding 2 |  |  |  |  |  |  |
| 1418272_at | OXCT2 | 3-oxoacid CoA transferase 2 |  |  |  |  |  |  |
| 1460459_at | PAQR5 | progestin and adipoQ receptor family member V |  |  |  |  |  |  |
| 1449298_a_at | PDE1A | phosphodiesterase 1A, calmodulin-dependent |  |  |  |  |  |  |
| 1416501_at | PDPK1 | 3-phosphoinositide dependent protein kinase-1 |  |  |  |  |  |  |
| 1431701_a_at | PDZK1 | PDZ domain containing 1 |  |  |  |  |  |  |
| 1421138_a_at | PKIB | protein kinase (cAMP-dependent, catalytic) inhibitor beta | |  |  |  |  |  |
| 1432460_at | Pldi | polymorphic derived intron containing |  |  |  |  |  |  |
| 1428345_at | PPAPDC2 | phosphatidic acid phosphatase type 2 domain containing 2 | |  |  |  |  |  |
| 1426621_a_at | PPP2R2B | protein phosphatase 2, regulatory subunit B, beta |  |  |  |  |  |  |
| 1437054_x_at | PRM1 | protamine 1 |  |  |  |  |  |  |
| 1448105_at | PRM2 | protamine 2 |  |  |  |  |  |  |
| 1422913_at | PRM3 | protamine 3 |  |  |  |  |  |  |
| 1449427_at | PRSS37 | protease, serine, 37 |  |  |  |  |  |  |
| 1429578_at | Prss52 | protease, serine, 52 |  |  |  |  |  |  |
| 1420278_at | PRSS58 | protease, serine, 58 |  |  |  |  |  |  |
| 1430128_a_at | REEP6 | receptor accessory protein 6 |  |  |  |  |  |  |
| 1456802_at | RNF133 | ring finger protein 133 |  |  |  |  |  |  |
| 1419368_a_at | RNF138 | ring finger protein 138 |  |  |  |  |  |  |
| 1449293_a_at | SKP2 | S-phase kinase-associated protein 2 (p45) |  |  |  |  |  |  |
| 1418358_at | SMCP | sperm mitochondria-associated cysteine-rich protein |  |  |  |  |  |  |
| 1420766_at | SOCS7 | suppressor of cytokine signaling 7 |  |  |  |  |  |  |
| 1430351_at | SPATA18 | spermatogenesis associated 18 homolog (rat) |  |  |  |  |  |  |
| 1453335_a_at | SPATA3 | spermatogenesis associated 3 |  |  |  |  |  |  |
| 1429864_at | SPATC1 | spermatogenesis and centriole associated 1 |  |  |  |  |  |  |
| 1417616_at | ST6GALNAC2 | ST6 (alpha-N-acetyl-neuraminyl-2,3-beta-galactosyl-1,3)-N-acetylgalactosaminide alpha-2,6-sialyltransferase 2 | | | | | | |
| 1440820_x_at | TMCO2 | transmembrane and coiled-coil domains 2 |  |  |  |  |  |  |
| 1429825_at | TMEM225 | transmembrane protein 225 |  |  |  |  |  |  |
| 1432566_at | TMSB10/TMSB4X | thymosin beta 4, X-linked |  |  |  |  |  |  |
| 1415924_at | TNP1 | transition protein 1 (during histone to protamine replacement) | |  |  |  |  |  |
| 1422419_s_at | TNP2 | transition protein 2 (during histone to protamine replacement) | |  |  |  |  |  |
| 1426084_a_at | TOR1AIP1 | torsin A interacting protein 1 |  |  |  |  |  |  |
| 1430106_at | TP53TG5 | TP53 target 5 |  |  |  |  |  |  |
| 1448501_at | TSPAN6 | tetraspanin 6 |  |  |  |  |  |  |
| 1418557_s_at | TSSK3 | testis-specific serine kinase 3 |  |  |  |  |  |  |
| 1418956_at | TSSK6 | testis-specific serine kinase 6 |  |  |  |  |  |  |
| 1417373_a_at | TUBA4A | tubulin, alpha 4a |  |  |  |  |  |  |
| 1437266_at | TXNDC2 | thioredoxin domain containing 2 (spermatozoa) |  |  |  |  |  |  |
| 1417033_at | UBE2G2 | ubiquitin-conjugating enzyme E2G 2 |  |  |  |  |  |  |
| 1460272_at | UBL4B | ubiquitin-like 4B |  |  |  |  |  |  |
| 1437955_at | UBQLNL | ubiquilin-like |  |  |  |  |  |  |
| 1433479_at | UBR7 | ubiquitin protein ligase E3 component n-recognin 7 (putative) | |  |  |  |  |  |
| 1425805_a_at | USP12 | ubiquitin specific peptidase 12 |  |  |  |  |  |  |
| 1453670_at | USP50 | ubiquitin specific peptidase 50 |  |  |  |  |  |  |
| 1430644_at | Wbscr25 | Williams Beuren syndrome chromosome region 25 (human) | |  |  |  |  |  |
| 1430082_at | WDR64 | WD repeat domain 64 |  |  |  |  |  |  |
| 1448145_at | WWP2 | WW domain containing E3 ubiquitin protein ligase 2 |  |  |  |  |  |  |

| **B. List of differentially up-regulated genes (7) associated with GRTH in testicular polysomes of wild type adult mice** | | | | | | | | |
| --- | --- | --- | --- | --- | --- | --- | --- | --- |
|  |  |  |  |  |  |  |  |  |
| **Affymetrix ID** | **Entrez Gene Name** | |  |  |  |  |  |  |
| 1416022_at | FABP5 | fatty acid binding protein 5 (psoriasis-associated) | | | | |  |  |
| 1421374_a_at | FXYD1 | FXYD domain containing ion transport regulator 1 | | | | |  |  |
| 1436858_at | MBNL2 | muscleblind-like 2 (Drosophila) | | |  |  |  |  |
| 1416762_at | S100A10 | S100 calcium binding protein A10 | | | |  |  |  |
| 1460351_at | S100A11 | S100 calcium binding protein A11 | | | |  |  |  |
| 1415823_at | Scd2 | stearoyl-Coenzyme A desaturase 2 | | | |  |  |  |
| 1420447_at | SULT1E1 | sulfotransferase family 1E, estrogen-preferring, member 1 | | | | | |  |
