# Supplementary material for: Role of Gonadotropin Regulated Testicular RNA Helicase (GRTH/DDX25) on Polysomal Associated mRNAs in Mouse Testis
Source: PLoS One. 2012 Mar 30;7(3):e32470. doi: 10.1371/journal.pone.0032470 (PMC3316541; doi:10.1371/journal.pone.0032470)
Supplement: Table S4 — Differentially regulated genes in spermatocytes of GRTH KO compared to wild type adult mice. A. List of down-regulated genes (139) in spermatocytes of GRTH−/− compared to wild type mice. B. List of up-regulated genes (52) in spermatocytes of GRTH−/− compared to wild type mice. (DOCX) [file pone.0032470.s008.docx]

| **Table S4. Differentially regulated genes in spermatocytes of GRTH KO compared to wild type adult mice.**  **A. List of down-regulated genes (139) in spermatocytes of GRTH KO compared to wild type adult mice** | | | | | |
| --- | --- | --- | --- | --- | --- |
|  |  |  |  |  |  |
| **Affymetrix ID** | **Symbol** | **Entrez Gene Name** |  |  |  |
| 1419151_at | 1700001J03Rik (includes others) | RIKEN cDNA 1700123K08 gene |  |  |  |
| 1431103_at | 1700003P14Rik | RIKEN cDNA 1700003P14 gene |  |  |  |
| 1430284_at | 1700008P02Rik | RIKEN cDNA 1700008P02 gene |  |  |  |
| 1432505_at | 1700009J07Rik | RIKEN cDNA 1700009J07 gene |  |  |  |
| 1429142_at | 1700012A03Rik | RIKEN cDNA 1700012A03 gene |  |  |  |
| 1459995_at | 1700015G11Rik | RIKEN cDNA 1700015G11 gene |  |  |  |
| 1420767_at | 1700019G17Rik/Cml2 | camello-like 2 |  |  |  |
| 1450999_a_at | 1700029H14Rik | RIKEN cDNA 1700029H14 gene |  |  |  |
| 1457057_at | 1700042G07Rik | RIKEN cDNA 1700042G07 gene |  |  |  |
| 1440768_x_at | 1700052I22Rik | RIKEN cDNA 1700052I22 gene |  |  |  |
| 1457421_at | 1700063D05Rik | RIKEN cDNA 1700063D05 gene |  |  |  |
| 1419337_at | 1700080E11Rik | RIKEN cDNA 1700080E11 gene |  |  |  |
| 1453447_at | 1700109H08Rik | RIKEN cDNA 1700109H08 gene |  |  |  |
| 1432500_at | 2410006F04Rik | RIKEN cDNA 2410006F04 gene |  |  |  |
| 1432989_at | 2900011F02Rik | RIKEN cDNA 2900011F02 gene |  |  |  |
| 1432687_at | 4833406M21Rik | RIKEN cDNA 4833406M21 gene |  |  |  |
| 1432299_at | 4921513I08Rik | RIKEN cDNA 4921513I08 gene |  |  |  |
| 1454050_at | 4921521D15Rik | RIKEN cDNA 4921521D15 gene |  |  |  |
| 1433419_at | 4930405A07Rik | RIKEN cDNA 4930405A07 gene |  |  |  |
| 1445661_at | 4930406D18Rik | RIKEN cDNA 4930406D18 gene |  |  |  |
| 1430872_at | 4930412O13Rik | RIKEN cDNA 4930412O13 gene |  |  |  |
| 1454204_at | 4930442G10Rik | RIKEN cDNA 4930442G10 gene |  |  |  |
| 1433259_at | 4930518C04Rik | RIKEN cDNA 4930518C04 gene |  |  |  |
| 1433269_at | 4930553M12Rik | RIKEN cDNA 4930553M12 gene |  |  |  |
| 1431509_at | 4933404G15Rik | RIKEN cDNA 4933404G15 gene |  |  |  |
| 1432258_at | 9430014N10Rik | RIKEN cDNA 9430014N10 gene |  |  |  |
| 1445715_at | A230001M10Rik | RIKEN cDNA A230001M10 gene |  |  |  |
| 1443313_at | A530058N18Rik | RIKEN cDNA A530058N18 gene |  |  |  |
| 1427958_at | ABHD10 | abhydrolase domain containing 10 |  |  |  |
| 1419626_at | Adam25/Adam39 | a disintegrin and metallopeptidase domain 25 (testase 2) | | |  |
| 1439649_at | ADC | arginine decarboxylase |  |  |  |
| 1427519_at | ADORA2A | adenosine A2a receptor |  |  |  |
| 1428821_at | AGPAT2 | 1-acylglycerol-3-phosphate O-acyltransferase 2 (lysophosphatidic acid acyltransferase, beta) | | | |
| 1451376_at | ATL3 | atlastin GTPase 3 |  |  |  |
| 1435331_at | BC094916/Pyhin1 | pyrin and HIN domain family, member 1 |  |  |  |
| 1430886_at | C10orf11 | chromosome 10 open reading frame 11 |  |  |  |
| 1452802_at | C10orf62 | chromosome 10 open reading frame 62 |  |  |  |
| 1430314_at | C14orf148 | chromosome 14 open reading frame 148 |  |  |  |
| 1430422_at | C20orf141 | chromosome 20 open reading frame 141 |  |  |  |
| 1428095_a_at | C2CD2L | C2CD2-like |  |  |  |
| 1430892_at | C2orf51 | chromosome 2 open reading frame 51 |  |  |  |
| 1453879_at | C4orf36 | chromosome 4 open reading frame 36 |  |  |  |
| 1453142_at | C7orf31 | chromosome 7 open reading frame 31 |  |  |  |
| 1448752_at | CA2 | carbonic anhydrase II |  |  |  |
| 1429744_at | CABS1 | calcium-binding protein, spermatid-specific 1 | |  |  |
| 1417461_at | CAP1 | CAP, adenylate cyclase-associated protein 1 (yeast) | |  |  |
| 1431668_at | CAPZB | capping protein (actin filament) muscle Z-line, beta | |  |  |
| 1432568_at | CBY3 | chibby homolog 3 (Drosophila) |  |  |  |
| 1428965_at | CCDC54 | coiled-coil domain containing 54 |  |  |  |
| 1449487_at | CCDC70 | coiled-coil domain containing 70 |  |  |  |
| 1448983_at | CDRT4 | CMT1A duplicated region transcript 4 |  |  |  |
| 1417795_at | CHL1 | cell adhesion molecule with homology to L1CAM (close homolog of L1) | | | |
| 1431575_at | CLEC4G | C-type lectin domain family 4, member G |  |  |  |
| 1423370_a_at | CSNK1G2 | casein kinase 1, gamma 2 |  |  |  |
| 1444630_at | D2Ertd127e | DNA segment, Chr 2, ERATO Doi 127, expressed | |  |  |
| 1418263_at | DDX25 | DEAD (Asp-Glu-Ala-Asp) box polypeptide 25 | |  |  |
| 1425256_a_at | DIXDC1 | DIX domain containing 1 |  |  |  |
| 1432196_a_at | DSCAML1 | Down syndrome cell adhesion molecule like 1 | |  |  |
| 1452912_at | DSCC1 | defective in sister chromatid cohesion 1 homolog (S. cerevisiae) | | | |
| 1453400_at | DYDC2 | DPY30 domain containing 2 |  |  |  |
| 1441286_at | EG667449 (includes others) | T cell receptor alpha variable 6-1 |  |  |  |
| 1456258_at | EMX2 | empty spiracles homeobox 2 |  |  |  |
| 1434301_at | Fam84b | family with sequence similarity 84, member B | |  |  |
| 1450063_at | Fmn2 (mouse) | formin 2 |  |  |  |
| 1434309_at | FNTB | farnesyltransferase, CAAX box, beta |  |  |  |
| 1422267_at | FOXB2 | forkhead box B2 |  |  |  |
| 1418948_at | FSCN3 | fascin homolog 3, actin-bundling protein, testicular (Strongylocentrotus purpuratus) | | | |
| 1419378_a_at | FXYD2 | FXYD domain containing ion transport regulator 2 | |  |  |
| 1452907_at | GALC | galactosylceramidase |  |  |  |
| 1426235_a_at | GLUL | glutamate-ammonia ligase |  |  |  |
| 1428323_at | GPD2 | glycerol-3-phosphate dehydrogenase 2 (mitochondrial) | | |  |
| 1456432_at | GRAP2 | GRB2-related adaptor protein 2 |  |  |  |
| 1425891_a_at | Grtp1 (mouse) | GH regulated TBC protein 1 |  |  |  |
| 1425165_at | Gzmn | granzyme N |  |  |  |
| 1451023_at | HCN3 | hyperpolarization activated cyclic nucleotide-gated potassium channel 3 | | | |
| 1419297_at | HLA-DOA | major histocompatibility complex, class II, DO alpha | |  |  |
| 1449497_at | IL12B | interleukin 12B (natural killer cell stimulatory factor 2, cytotoxic lymphocyte maturation factor 2, p40) | | | |
| 1450370_a_at | KCNIP4 | Kv channel interacting protein 4 |  |  |  |
| 1428602_at | KCNJ9 | potassium inwardly-rectifying channel, subfamily J, member 9 | | |  |
| 1431613_a_at | KCNK10 | potassium channel, subfamily K, member 10 | |  |  |
| 1449463_at | Klk1b1 (includes others) | kallikrein 1-related pepidase b4 |  |  |  |
| 1432180_at | LIPE | lipase, hormone-sensitive |  |  |  |
| 1444710_at | LOC100503197 | hypothetical LOC100503197 |  |  |  |
| 1432551_at | LOC73317 | RIKEN cDNA 1700031F10 gene |  |  |  |
| 1448998_at | LPO | lactoperoxidase |  |  |  |
| 1451022_at | LRP6 | low density lipoprotein receptor-related protein 6 | |  |  |
| 1430057_s_at | LRRC57 | leucine rich repeat containing 57 |  |  |  |
| 1430821_at | LYRM7 | Lyrm7 homolog (mouse) |  |  |  |
| 1425393_a_at | MAP2K7 | mitogen-activated protein kinase kinase 7 |  |  |  |
| 1440880_at | MPPE1 | metallophosphoesterase 1 |  |  |  |
| 1438351_at | MSX2 | msh homeobox 2 |  |  |  |
| 1421592_at | NCAM2 | neural cell adhesion molecule 2 |  |  |  |
| 1453777_a_at | NDST3 | N-deacetylase/N-sulfotransferase (heparan glucosaminyl) 3 | | |  |
| 1416816_at | NEK7 | NIMA (never in mitosis gene a)-related kinase 7 | |  |  |
| 1453236_at | NIPSNAP3B | nipsnap homolog 3B (C. elegans) |  |  |  |
| 1432342_at | NMNAT3 | nicotinamide nucleotide adenylyltransferase 3 | |  |  |
| 1438086_at | NPY6R | neuropeptide Y receptor Y6 (pseudogene) | |  |  |
| 1421515_at | NR6A1 | nuclear receptor subfamily 6, group A, member 1 | |  |  |
| 1437010_a_at | OAZ3 | ornithine decarboxylase antizyme 3 |  |  |  |
| 1457659_x_at | ODF1 | outer dense fiber of sperm tails 1 |  |  |  |
| 1418272_at | OXCT2 | 3-oxoacid CoA transferase 2 |  |  |  |
| 1425521_at | PAIP1 | poly(A) binding protein interacting protein 1 | |  |  |
| 1420798_s_at | PCDHA11 | protocadherin alpha 11 |  |  |  |
| 1419327_at | PDXDC1 | pyridoxal-dependent decarboxylase domain containing 1 | | |  |
| 1452485_at | PHOSPHO1 | phosphatase, orphan 1 |  |  |  |
| 1452590_a_at | PLAC9 | placenta-specific 9 |  |  |  |
| 1432421_at | PMS2 | PMS2 postmeiotic segregation increased 2 (S. cerevisiae) | | |  |
| 1451943_a_at | PPM1A | protein phosphatase, Mg2+/Mn2+ dependent, 1A | |  |  |
| 1426621_a_at | PPP2R2B | protein phosphatase 2, regulatory subunit B, beta | |  |  |
| 1448105_at | PRM2 | protamine 2 |  |  |  |
| 1422913_at | PRM3 | protamine 3 |  |  |  |
| 1449259_at | RAB3D | RAB3D, member RAS oncogene family |  |  |  |
| 1452355_at | RD3 | retinal degeneration 3 |  |  |  |
| 1430978_at | RPS25 | ribosomal protein S25 |  |  |  |
| 1422194_at | SCN5A | sodium channel, voltage-gated, type V, alpha subunit | |  |  |
| 1430374_at | SLC25A37 | solute carrier family 25, member 37 |  |  |  |
| 1428752_at | SLC5A11 | solute carrier family 5 (sodium/glucose cotransporter), member 11 | | | |
| 1435524_at | Snhg8 | small nucleolar RNA host gene 8 |  |  |  |
| 1430351_at | SPATA18 | spermatogenesis associated 18 homolog (rat) | |  |  |
| 1429923_x_at | SPATA3 | spermatogenesis associated 3 |  |  |  |
| 1431384_at | SPEM1 | spermatid maturation 1 |  |  |  |
| 1452617_at | SSBP1 | single-stranded DNA binding protein 1 |  |  |  |
| 1417616_at | ST6GALNAC2 | ST6 (alpha-N-acetyl-neuraminyl-2,3-beta-galactosyl-1,3)-N-acetylgalactosaminide alpha-2,6-sialyltransferase 2 | | | |
| 1444074_at | SYNE2 | spectrin repeat containing, nuclear envelope 2 | |  |  |
| 1416783_at | TAC1 | tachykinin, precursor 1 |  |  |  |
| 1426612_at | TIPIN | TIMELESS interacting protein |  |  |  |
| 1429825_at | TMEM225 | transmembrane protein 225 |  |  |  |
| 1431404_at | TMEM56 | transmembrane protein 56 |  |  |  |
| 1422419_s_at | TNP2 | transition protein 2 (during histone to protamine replacement) | | |  |
| 1436533_at | TROVE2 | TROVE domain family, member 2 |  |  |  |
| 1418557_s_at | TSSK3 | testis-specific serine kinase 3 |  |  |  |
| 1418956_at | TSSK6 | testis-specific serine kinase 6 |  |  |  |
| 1457149_at | TTC22 | tetratricopeptide repeat domain 22 |  |  |  |
| 1437955_at | UBQLNL | ubiquilin-like |  |  |  |
| 1422932_a_at | VAV1 | vav 1 guanine nucleotide exchange factor |  |  |  |
| 1418486_at | VNN1 | vanin 1 |  |  |  |
| 1430644_at | Wbscr25 | Williams Beuren syndrome chromosome region 25 (human) | | |  |
| 1431335_a_at | WFDC1 | WAP four-disulfide core domain 1 |  |  |  |
| 1426614_at | ZMYND8 | zinc finger, MYND-type containing 8 |  |  |  |

| **B. List of up-regulated genes (51) in spermatocytes of GRTH KO compared to wild type adult mice** | | | | |
| --- | --- | --- | --- | --- |
|  |  |  |  |  |
| **Affymetrix ID** | **Symbol** | **Entrez Gene Name** |  |  |
| 1456857_at | 1500011B03Rik | RIKEN cDNA 1500011B03 gene |  |  |
| 1449239_at | 1700045I19Rik | ring finger protein 138 pseudogene |  |  |
| 1453925_at | 4930429F24Rik | RIKEN cDNA 4930429F24 gene |  |  |
| 1454341_at | 4933404I11Rik | RIKEN cDNA 4933404I11 gene |  |  |
| 1435827_at | 4933404O12Rik | RIKEN cDNA 4933404O12 gene |  |  |
| 1455370_at | A630023P12Rik | RIKEN cDNA A630023P12 gene |  |  |
| 1437441_at | AA388235 | expressed sequence AA388235 |  |  |
| 1433453_a_at | ABTB2 | ankyrin repeat and BTB (POZ) domain containing 2 | | |
| 1421002_at | ANGPTL2 | angiopoietin-like 2 |  |  |
| 1441978_at | AQP6 | aquaporin 6, kidney specific |  |  |
| 1452732_at | ASPRV1 | aspartic peptidase, retroviral-like 1 |  |  |
| 1422279_at | AU040096/Fv1 | Friend virus susceptibility 1 |  |  |
| 1455826_a_at | BACE1 | beta-site APP-cleaving enzyme 1 |  |  |
| 1452257_at | BDH1 | 3-hydroxybutyrate dehydrogenase, type 1 | |  |
| 1424694_at | C20orf108 | chromosome 20 open reading frame 108 | |  |
| 1433988_s_at | C230098O21Rik | RIKEN cDNA C230098O21 gene |  |  |
| 1424186_at | CCDC80 | coiled-coil domain containing 80 |  |  |
| 1427428_at | CLEC4G | C-type lectin domain family 4, member G | |  |
| 1419582_at | CYP2C18 | cytochrome P450, family 2, subfamily C, polypeptide 18 | | |
| 1419704_at | CYP3A4 | cytochrome P450, family 3, subfamily A, polypeptide 4 | | |
| 1418550_x_at | Defa-rs1 (includes others) (mouse) | defensin, alpha, related sequence 1 |  |  |
| 1424047_at | DERA | deoxyribose-phosphate aldolase (putative) | |  |
| 1424065_at | EDEM1 | ER degradation enhancer, mannosidase alpha-like 1 | | |
| 1436926_at | ESRRB | estrogen-related receptor beta |  |  |
| 1448620_at | FCGR2A | Fc fragment of IgG, low affinity IIa, receptor (CD32) | | |
| 1419907_s_at | FCRLA | Fc receptor-like A |  |  |
| 1424537_at | Gm10921 (includes others) | predicted gene 14346 |  |  |
| 1443995_at | Gm9 | predicted gene 9 |  |  |
| 1450346_at | GPR50 | G protein-coupled receptor 50 |  |  |
| 1423290_at | HYOU1 | hypoxia up-regulated 1 |  |  |
| 1421551_s_at | Ifi202b | interferon activated gene 202B |  |  |
| 1418600_at | KLF1 | Kruppel-like factor 1 (erythroid) |  |  |
| 1446154_at | LOC100047123 | hypothetical protein LOC100047123 |  |  |
| 1455871_s_at | LOC388344 | ribosomal protein L13 pseudogene 12 | |  |
| 1449440_at | LPIN3 | lipin 3 |  |  |
| 1434931_at | NEO1 | neogenin 1 |  |  |
| 1431518_at | PTCHD3 | patched domain containing 3 |  |  |
| 1417676_a_at | PTPRO | protein tyrosine phosphatase, receptor type, O | |  |
| 1449048_s_at | RAB4A | RAB4A, member RAS oncogene family | |  |
| 1449319_at | RSPO1 | R-spondin homolog (Xenopus laevis) | |  |
| 1439882_at | SEC23IP | SEC23 interacting protein |  |  |
| 1418739_at | SGK2 | serum/glucocorticoid regulated kinase 2 | |  |
| 1447517_at | SKIV2L2 | superkiller viralicidic activity 2-like 2 (S. cerevisiae) | | |
| 1439368_a_at | SLC9A3R2 | solute carrier family 9 (sodium/hydrogen exchanger), member 3 regulator 2 | | |
| 1420884_at | SLN | sarcolipin |  |  |
| 1453391_at | Speer7-ps1 | spermatogenesis associated glutamate (E)-rich protein 7, pseudogene 1 | | |
| 1457352_x_at | SVOPL | SVOP-like |  |  |
| 1433498_at | URGCP | upregulator of cell proliferation |  |  |
| 1435244_at | VAV2 | vav 2 guanine nucleotide exchange factor | |  |
| 1437358_at | WDFY1 | WD repeat and FYVE domain containing 1 | |  |
| 1430024_at | YIPF6 | Yip1 domain family, member 6 |  |  |
