# Supplementary material for: Role of Gonadotropin Regulated Testicular RNA Helicase (GRTH/DDX25) on Polysomal Associated mRNAs in Mouse Testis
Source: PLoS One. 2012 Mar 30;7(3):e32470. doi: 10.1371/journal.pone.0032470 (PMC3316541; doi:10.1371/journal.pone.0032470)
Supplement: Table S5 — Differentially regulated genes in round spermatids of GRTH KO compared to wild type adult mice. A. List of down-regulated genes (216) in round spermatids of GRTH−/− compared to wild type mice. B. List of up-regulated genes (326) in round spermatids of GRTH−/− compared to wild type mice. (DOCX) [file pone.0032470.s009.docx]

| **Table S5. Differentially regulated genes in round spermatids of GRTH KO compared to wild type adult mice.**  **A. List of down-regulated genes (216) in round spermatids of GRTH KO compared to wild type adult mice** | | | | | | | |
| --- | --- | --- | --- | --- | --- | --- | --- |
|  |  |  |  |  |  |  |  |
| **Affymetrix ID** | **Symbol** | **Entrez Gene Name** |  |  |  |  |  |
| 1431103_at | 1700003P14Rik | RIKEN cDNA 1700003P14 gene |  |  |  |  |  |
| 1430284_at | 1700008P02Rik | RIKEN cDNA 1700008P02 gene |  |  |  |  |  |
| 1432505_at | 1700009J07Rik | RIKEN cDNA 1700009J07 gene |  |  |  |  |  |
| 1429142_at | 1700012A03Rik | RIKEN cDNA 1700012A03 gene |  |  |  |  |  |
| 1459995_at | 1700015G11Rik | RIKEN cDNA 1700015G11 gene |  |  |  |  |  |
| 1420767_at | 1700019G17Rik/Cml2 | camello-like 2 |  |  |  |  |  |
| 1447710_at | 1700019P21Rik | RIKEN cDNA 1700019P21 gene |  |  |  |  |  |
| 1429855_at | 1700023I07Rik | protein phosphatase 1, regulatory (inhibitor) subunit 2 pseudogene | | | |  |  |
| 1429828_at | 1700024P04Rik | RIKEN cDNA 1700024P04 gene |  |  |  |  |  |
| 1432779_at | 1700037N05Rik | RIKEN cDNA 1700037N05 gene |  |  |  |  |  |
| 1457057_at | 1700042G07Rik | RIKEN cDNA 1700042G07 gene |  |  |  |  |  |
| 1442131_at | 1700048M11Rik | RIKEN cDNA 1700048M11 gene |  |  |  |  |  |
| 1440768_x_at | 1700052I22Rik | RIKEN cDNA 1700052I22 gene |  |  |  |  |  |
| 1457421_at | 1700063D05Rik | RIKEN cDNA 1700063D05 gene |  |  |  |  |  |
| 1419337_at | 1700080E11Rik | RIKEN cDNA 1700080E11 gene |  |  |  |  |  |
| 1433328_at | 1700095A21Rik | RIKEN cDNA 1700095A21 gene |  |  |  |  |  |
| 1453447_at | 1700109H08Rik | RIKEN cDNA 1700109H08 gene |  |  |  |  |  |
| 1424439_at | 1810065E05Rik | RIKEN cDNA 1810065E05 gene |  |  |  |  |  |
| 1433152_at | 2900022B07Rik | RIKEN cDNA 2900022B07 gene |  |  |  |  |  |
| 1432299_at | 4921513I08Rik | RIKEN cDNA 4921513I08 gene |  |  |  |  |  |
| 1430254_at | 4921517D22Rik | RIKEN cDNA 4921517D22 gene |  |  |  |  |  |
| 1454050_at | 4921521D15Rik | RIKEN cDNA 4921521D15 gene |  |  |  |  |  |
| 1419718_at | 4921530L21Rik | RIKEN cDNA 4921530L21 gene |  |  |  |  |  |
| 1433419_at | 4930405A07Rik | RIKEN cDNA 4930405A07 gene |  |  |  |  |  |
| 1445661_at | 4930406D18Rik | RIKEN cDNA 4930406D18 gene |  |  |  |  |  |
| 1430872_at | 4930412O13Rik | RIKEN cDNA 4930412O13 gene |  |  |  |  |  |
| 1431184_a_at | 4930503B20Rik | RIKEN cDNA 4930503B20 gene |  |  |  |  |  |
| 1433259_at | 4930518C04Rik | RIKEN cDNA 4930518C04 gene |  |  |  |  |  |
| 1454133_s_at | 4930523O13Rik | RIKEN cDNA 4930523O13 gene |  |  |  |  |  |
| 1421367_at | 4930549C01Rik | RIKEN cDNA 4930549C01 gene |  |  |  |  |  |
| 1432223_at | 4930552N02Rik | RIKEN cDNA 4930552N02 gene |  |  |  |  |  |
| 1433218_at | 4930566N20Rik | RIKEN cDNA 4930566N20 gene |  |  |  |  |  |
| 1431863_at | 4931430N09Rik | RIKEN cDNA 4931430N09 gene |  |  |  |  |  |
| 1430094_at | 4931440L10Rik | RIKEN cDNA 4931440L10 gene |  |  |  |  |  |
| 1432210_at | 4933401H06Rik | RIKEN cDNA 4933401H06 gene |  |  |  |  |  |
| 1431476_at | 4933407I05Rik | RIKEN cDNA 4933407I05 gene |  |  |  |  |  |
| 1433081_at | 4933407I18Rik | RIKEN cDNA 4933407I18 gene |  |  |  |  |  |
| 1430958_at | 4933430N04Rik | RIKEN cDNA 4933430N04 gene |  |  |  |  |  |
| 1431925_at | 4933433H22Rik | RIKEN cDNA 4933433H22 gene |  |  |  |  |  |
| 1433050_at | 5730478J17Rik | RIKEN cDNA 5730478J17 gene |  |  |  |  |  |
| 1442517_a_at | 9630013A20Rik | RIKEN cDNA 9630013A20 gene |  |  |  |  |  |
| 1445721_at | A830021M18Rik | RIKEN cDNA A830021M18 gene |  |  |  |  |  |
| 1419626_at | Adam25/Adam39 | a disintegrin and metallopeptidase domain 25 (testase 2) | | |  |  |  |
| 1439649_at | ADC | arginine decarboxylase |  |  |  |  |  |
| 1444193_at | ADHFE1 | alcohol dehydrogenase, iron containing, 1 | |  |  |  |  |
| 1428821_at | AGPAT2 | 1-acylglycerol-3-phosphate O-acyltransferase 2 (lysophosphatidic acid acyltransferase, beta) | | | | | |
| 1430395_at | ANKRD45 | ankyrin repeat domain 45 |  |  |  |  |  |
| 1430571_s_at | ARMC6 | armadillo repeat containing 6 |  |  |  |  |  |
| 1430632_at | ASB1 | ankyrin repeat and SOCS box containing 1 | |  |  |  |  |
| 1451376_at | ATL3 | atlastin GTPase 3 |  |  |  |  |  |
| 1435331_at | BC094916/Pyhin1 | pyrin and HIN domain family, member 1 | |  |  |  |  |
| 1428743_at | BRI3BP | BRI3 binding protein |  |  |  |  |  |
| 1451824_at | BTN1A1 | butyrophilin, subfamily 1, member A1 | |  |  |  |  |
| 1423040_at | BZW1 | basic leucine zipper and W2 domains 1 | |  |  |  |  |
| 1430887_s_at | C10orf11 | chromosome 10 open reading frame 11 | |  |  |  |  |
| 1429414_at | C10orf90 | chromosome 10 open reading frame 90 | |  |  |  |  |
| 1428593_at | C13orf27 | chromosome 13 open reading frame 27 | |  |  |  |  |
| 1430314_at | C14orf148 | chromosome 14 open reading frame 148 | |  |  |  |  |
| 1423463_a_at | C15orf23 | chromosome 15 open reading frame 23 | |  |  |  |  |
| 1452785_at | C18orf55 | chromosome 18 open reading frame 55 | |  |  |  |  |
| 1430422_at | C20orf141 | chromosome 20 open reading frame 141 | |  |  |  |  |
| 1432308_at | C20orf71 | chromosome 20 open reading frame 71 | |  |  |  |  |
| 1430892_at | C2orf51 | chromosome 2 open reading frame 51 | |  |  |  |  |
| 1453879_at | C4orf36 | chromosome 4 open reading frame 36 | |  |  |  |  |
| 1436832_at | C4orf52 | chromosome 4 open reading frame 52 | |  |  |  |  |
| 1449654_s_at | C77545 | expressed sequence C77545 |  |  |  |  |  |
| 1453142_at | C7orf31 | chromosome 7 open reading frame 31 | |  |  |  |  |
| 1424795_a_at | C9orf50 | chromosome 9 open reading frame 50 | |  |  |  |  |
| 1448752_at | CA2 | carbonic anhydrase II |  |  |  |  |  |
| 1429744_at | CABS1 | calcium-binding protein, spermatid-specific 1 | |  |  |  |  |
| 1431668_at | CAPZB | capping protein (actin filament) muscle Z-line, beta | | |  |  |  |
| 1432568_at | CBY3 | chibby homolog 3 (Drosophila) |  |  |  |  |  |
| 1428965_at | CCDC54 | coiled-coil domain containing 54 |  |  |  |  |  |
| 1449487_at | CCDC70 | coiled-coil domain containing 70 |  |  |  |  |  |
| 1432346_a_at | CDH23 | cadherin-related 23 |  |  |  |  |  |
| 1415956_a_at | CDK16 | cyclin-dependent kinase 16 |  |  |  |  |  |
| 1448983_at | CDRT4 | CMT1A duplicated region transcript 4 | |  |  |  |  |
| 1450257_at | CER1 | cerberus 1, cysteine knot superfamily, homolog (Xenopus laevis) | | | |  |  |
| 1425321_a_at | CLMN | calmin (calponin-like, transmembrane) | |  |  |  |  |
| 1449563_at | CNTN1 | contactin 1 |  |  |  |  |  |
| 1425234_at | COL20A1 | collagen, type XX, alpha 1 |  |  |  |  |  |
| 1423370_a_at | CSNK1G2 | casein kinase 1, gamma 2 |  |  |  |  |  |
| 1431405_a_at | CSPP1 | centrosome and spindle pole associated protein 1 | | |  |  |  |
| 1441560_at | CTNNA3 | catenin (cadherin-associated protein), alpha 3 | | |  |  |  |
| 1438304_at | CXXC4 | CXXC finger protein 4 |  |  |  |  |  |
| 1418821_at | Cyp2a12/Cyp2a22 | cytochrome P450, family 2, subfamily a, polypeptide 12 | | |  |  |  |
| 1435940_at | DCLK1 | doublecortin-like kinase 1 |  |  |  |  |  |
| 1431933_a_at | DCP1A | DCP1 decapping enzyme homolog A (S. cerevisiae) | | |  |  |  |
| 1418263_at | DDX25 | DEAD (Asp-Glu-Ala-Asp) box polypeptide 25 | |  |  |  |  |
| 1452546_x_at | Defb10/Defb11 | defensin beta 11 |  |  |  |  |  |
| 1421807_at | Defb6 | defensin beta 6 |  |  |  |  |  |
| 1426465_at | DLGAP4 | discs, large (Drosophila) homolog-associated protein 4 | | |  |  |  |
| 1432196_a_at | DSCAML1 | Down syndrome cell adhesion molecule like 1 | |  |  |  |  |
| 1453400_at | DYDC2 | DPY30 domain containing 2 |  |  |  |  |  |
| 1453619_at | EFCAB1 | EF-hand calcium binding domain 1 |  |  |  |  |  |
| 1421788_x_at | Egfbp2 | epidermal growth factor binding protein type B | | |  |  |  |
| 1444010_at | EIF4E | eukaryotic translation initiation factor 4E | |  |  |  |  |
| 1441777_at | EMX1 | empty spiracles homeobox 1 |  |  |  |  |  |
| 1431761_at | ENTPD4 | ectonucleoside triphosphate diphosphohydrolase 4 | | |  |  |  |
| 1430685_at | FAM131B | family with sequence similarity 131, member B | | |  |  |  |
| 1434301_at | Fam84b | family with sequence similarity 84, member B | |  |  |  |  |
| 1426042_at | FGD4 | FYVE, RhoGEF and PH domain containing 4 | |  |  |  |  |
| 1427671_a_at | Fmn1 | formin 1 |  |  |  |  |  |
| 1422267_at | FOXB2 | forkhead box B2 |  |  |  |  |  |
| 1437138_at | FOXM1 | forkhead box M1 |  |  |  |  |  |
| 1418948_at | FSCN3 | fascin homolog 3, actin-bundling protein, testicular (Strongylocentrotus purpuratus) | | | | | |
| 1449655_a_at | FTSJD2 | FtsJ methyltransferase domain containing 2 | |  |  |  |  |
| 1422998_a_at | GLRX2 | glutaredoxin 2 |  |  |  |  |  |
| 1426235_a_at | GLUL | glutamate-ammonia ligase |  |  |  |  |  |
| 1454140_at | Gm11149 | predicted gene 11149 |  |  |  |  |  |
| 1420997_a_at | GPI | glucose-6-phosphate isomerase |  |  |  |  |  |
| 1439150_x_at | Grtp1 (mouse) | GH regulated TBC protein 1 |  |  |  |  |  |
| 1421061_at | GUCA1A | guanylate cyclase activator 1A (retina) | |  |  |  |  |
| 1425165_at | Gzmn | granzyme N |  |  |  |  |  |
| 1430869_a_at | HABP4 | hyaluronan binding protein 4 |  |  |  |  |  |
| 1451023_at | HCN3 | hyperpolarization activated cyclic nucleotide-gated potassium channel 3 | | | | |  |
| 1435626_a_at | HERPUD1 | homocysteine-inducible, endoplasmic reticulum stress-inducible, ubiquitin-like domain member 1 | | | | | |
| 1415982_at | HERPUD2 | HERPUD family member 2 |  |  |  |  |  |
| 1425988_a_at | HIPK1 | homeodomain interacting protein kinase 1 | |  |  |  |  |
| 1419625_at | HSPA1L | heat shock 70kDa protein 1-like |  |  |  |  |  |
| 1458481_at | IL2RG | interleukin 2 receptor, gamma |  |  |  |  |  |
| 1454109_a_at | JMJD6 | jumonji domain containing 6 |  |  |  |  |  |
| 1435094_at | KCNJ16 | potassium inwardly-rectifying channel, subfamily J, member 16 | | | |  |  |
| 1421342_at | KCNS2 | potassium voltage-gated channel, delayed-rectifier, subfamily S, member 2 | | | | |  |
| 1454786_at | KIAA0930 | KIAA0930 |  |  |  |  |  |
| 1432464_a_at | KIAA1543 | KIAA1543 |  |  |  |  |  |
| 1430255_at | KLF5 | Kruppel-like factor 5 (intestinal) |  |  |  |  |  |
| 1415837_at | Klk1b1 (includes others) | kallikrein 1-related pepidase b4 |  |  |  |  |  |
| 1432353_at | Larp1b | La ribonucleoprotein domain family, member 1B | | |  |  |  |
| 1448207_at | LASP1 | LIM and SH3 protein 1 |  |  |  |  |  |
| 1453235_at | Lelp1 | late cornified envelope-like proline-rich 1 | |  |  |  |  |
| 1422820_at | LIPE | lipase, hormone-sensitive |  |  |  |  |  |
| 1453632_at | LOC100129924 | hypothetical LOC100129924 |  |  |  |  |  |
| 1438813_at | LOC100502820 | hypothetical LOC100502820 |  |  |  |  |  |
| 1444710_at | LOC100503197 | hypothetical LOC100503197 |  |  |  |  |  |
| 1440967_at | LOC100504231 | hypothetical LOC100504231 |  |  |  |  |  |
| 1429872_at | LOC346702 | protease, serine, 51-like |  |  |  |  |  |
| 1432551_at | LOC73317 | RIKEN cDNA 1700031F10 gene |  |  |  |  |  |
| 1444906_at | LPHN2 | latrophilin 2 |  |  |  |  |  |
| 1448998_at | LPO | lactoperoxidase |  |  |  |  |  |
| 1430057_s_at | LRRC57 | leucine rich repeat containing 57 |  |  |  |  |  |
| 1419135_at | LTB | lymphotoxin beta (TNF superfamily, member 3) | | |  |  |  |
| 1425393_a_at | MAP2K7 | mitogen-activated protein kinase kinase 7 | |  |  |  |  |
| 1425803_a_at | MBD2 | methyl-CpG binding domain protein 2 | |  |  |  |  |
| 1432675_at | MDN1 | MDN1, midasin homolog (yeast) |  |  |  |  |  |
| 1458015_at | MEGF11 | multiple EGF-like-domains 11 |  |  |  |  |  |
| 1430331_at | METTL23 | methyltransferase like 23 |  |  |  |  |  |
| 1423489_at | MMD | monocyte to macrophage differentiation-associated | | |  |  |  |
| 1452990_at | MTF1 | metal-regulatory transcription factor 1 | |  |  |  |  |
| 1431813_at | MYO18B | myosin XVIIIB |  |  |  |  |  |
| 1418781_at | NAT9 | N-acetyltransferase 9 (GCN5-related, putative) | | |  |  |  |
| 1451872_a_at | NEURL | neuralized homolog (Drosophila) |  |  |  |  |  |
| 1448728_a_at | NFKBIZ | nuclear factor of kappa light polypeptide gene enhancer in B-cells inhibitor, zeta | | | | | |
| 1422057_at | NODAL | nodal homolog (mouse) |  |  |  |  |  |
| 1421181_at | Npcd | neuronal pentraxin chromo domain |  |  |  |  |  |
| 1449983_a_at | NQO2 | NAD(P)H dehydrogenase, quinone 2 | |  |  |  |  |
| 1440821_x_at | ODF1 | outer dense fiber of sperm tails 1 |  |  |  |  |  |
| 1439918_at | Odf2 | outer dense fiber of sperm tails 2 |  |  |  |  |  |
| 1439666_at | ODF3B | outer dense fiber of sperm tails 3B |  |  |  |  |  |
| 1418272_at | OXCT2 | 3-oxoacid CoA transferase 2 |  |  |  |  |  |
| 1460459_at | PAQR5 | progestin and adipoQ receptor family member V | | |  |  |  |
| 1419327_at | PDXDC1 | pyridoxal-dependent decarboxylase domain containing 1 | | |  |  |  |
| 1452485_at | PHOSPHO1 | phosphatase, orphan 1 |  |  |  |  |  |
| 1452590_a_at | PLAC9 | placenta-specific 9 |  |  |  |  |  |
| 1420839_at | PLEKHA3 | pleckstrin homology domain containing, family A (phosphoinositide binding specific) member 3 | | | | | |
| 1451093_at | POLR2E | polymerase (RNA) II (DNA directed) polypeptide E, 25kDa | | | |  |  |
| 1422220_at | POU1F1 | POU class 1 homeobox 1 |  |  |  |  |  |
| 1451943_a_at | PPM1A | protein phosphatase, Mg2+/Mn2+ dependent, 1A | | |  |  |  |
| 1421822_at | PPP2CB | protein phosphatase 2, catalytic subunit, beta isozyme | | |  |  |  |
| 1426621_a_at | PPP2R2B | protein phosphatase 2, regulatory subunit B, beta | | |  |  |  |
| 1422847_a_at | PRKCD | protein kinase C, delta |  |  |  |  |  |
| 1426044_a_at | PRKCQ | protein kinase C, theta |  |  |  |  |  |
| 1418181_at | PTP4A3 | protein tyrosine phosphatase type IVA, member 3 | | |  |  |  |
| 1452284_at | PTPRZ1 | protein tyrosine phosphatase, receptor-type, Z polypeptide 1 | | | |  |  |
| 1436336_at | PTRH1 | peptidyl-tRNA hydrolase 1 homolog (S. cerevisiae) | | |  |  |  |
| 1423905_at | Pvr | poliovirus receptor |  |  |  |  |  |
| 1453406_a_at | RAB28 | RAB28, member RAS oncogene family | |  |  |  |  |
| 1452355_at | RD3 | retinal degeneration 3 |  |  |  |  |  |
| 1430128_a_at | REEP6 | receptor accessory protein 6 |  |  |  |  |  |
| 1421242_at | RNF144A | ring finger protein 144A |  |  |  |  |  |
| 1444666_at | RPF2 | ribosome production factor 2 homolog (S. cerevisiae) | | |  |  |  |
| 1430978_at | RPS25 | ribosomal protein S25 |  |  |  |  |  |
| 1449246_at | RUNDC3A | RUN domain containing 3A |  |  |  |  |  |
| 1449293_a_at | SKP2 | S-phase kinase-associated protein 2 (p45) | |  |  |  |  |
| 1419315_at | SLAMF9 | SLAM family member 9 |  |  |  |  |  |
| 1430374_at | SLC25A37 | solute carrier family 25, member 37 | |  |  |  |  |
| 1417042_at | SLC37A4 | solute carrier family 37 (glucose-6-phosphate transporter), member 4 | | | | |  |
| 1453687_at | SLC4A3 | solute carrier family 4, anion exchanger, member 3 | | |  |  |  |
| 1440779_s_at | SLC5A9 | solute carrier family 5 (sodium/glucose cotransporter), member 9 | | | |  |  |
| 1420766_at | SOCS7 | suppressor of cytokine signaling 7 |  |  |  |  |  |
| 1430351_at | SPATA18 | spermatogenesis associated 18 homolog (rat) | |  |  |  |  |
| 1429923_x_at | SPATA3 | spermatogenesis associated 3 |  |  |  |  |  |
| 1429864_at | SPATC1 | spermatogenesis and centriole associated 1 | |  |  |  |  |
| 1431384_at | SPEM1 | spermatid maturation 1 |  |  |  |  |  |
| 1431719_a_at | SRGAP1 | SLIT-ROBO Rho GTPase activating protein 1 | |  |  |  |  |
| 1454246_at | ST3GAL6 | ST3 beta-galactoside alpha-2,3-sialyltransferase 6 | | |  |  |  |
| 1425444_a_at | TGFBR2 | transforming growth factor, beta receptor II (70/80kDa) | | |  |  |  |
| 1453843_at | THAP6 | THAP domain containing 6 |  |  |  |  |  |
| 1449554_at | TLE3 | transducin-like enhancer of split 3 (E(sp1) homolog, Drosophila) | | | |  |  |
| 1431404_at | TMEM56 | transmembrane protein 56 |  |  |  |  |  |
| 1449969_at | TMOD4 | tropomodulin 4 (muscle) |  |  |  |  |  |
| 1418557_s_at | TSSK3 | testis-specific serine kinase 3 |  |  |  |  |  |
| 1418956_at | TSSK6 | testis-specific serine kinase 6 |  |  |  |  |  |
| 1429740_at | TTC7A | tetratricopeptide repeat domain 7A |  |  |  |  |  |
| 1417033_at | UBE2G2 | ubiquitin-conjugating enzyme E2G 2 (UBC7 homolog, yeast) | | | |  |  |
| 1420392_at | UBL4B | ubiquitin-like 4B |  |  |  |  |  |
| 1437955_at | UBQLNL | ubiquilin-like |  |  |  |  |  |
| 1433479_at | UBR7 | ubiquitin protein ligase E3 component n-recognin 7 (putative) | | | |  |  |
| 1423397_at | UGT2B17 | UDP glucuronosyltransferase 2 family, polypeptide B17 | | |  |  |  |
| 1422136_at | UHMK1 | U2AF homology motif (UHM) kinase 1 | |  |  |  |  |
| 1422377_at | Vmn1r49 (includes others) | vomeronasal 1, receptor 49 |  |  |  |  |  |
| 1418486_at | VNN1 | vanin 1 |  |  |  |  |  |
| 1453258_at | Wbscr25 | Williams Beuren syndrome chromosome region 25 (human) | | | |  |  |
| 1431335_a_at | WFDC1 | WAP four-disulfide core domain 1 |  |  |  |  |  |
| 1417805_at | XPNPEP2 | X-prolyl aminopeptidase (aminopeptidase P) 2, membrane-bound | | | |  |  |
| 1426679_at | ZNF706 | zinc finger protein 706 |  |  |  |  |  |

| **B. List of up-regulated genes (326) in round spermatids of GRTH KO compared to wild type adult mice** | | | | | |  |  |  |
| --- | --- | --- | --- | --- | --- | --- | --- | --- |
|  |  |  |  |  |  |  |  |  |
| **Affymetrix ID** | **Symbol** | **Entrez Gene Name** |  |  |  |  |  |  |
| 1432562_at | 1110006G14Rik | RIKEN cDNA 1110006G14 gene |  |  |  |  |  |  |
| 1454586_at | 1500002K03Rik | RIKEN cDNA 1500002K03 gene |  |  |  |  |  |  |
| 1433297_at | 1700039M15Rik | RIKEN cDNA 1700039M15 gene |  |  |  |  |  |  |
| 1421489_a_at | 2010106E10Rik | RIKEN cDNA 2010106E10 gene |  |  |  |  |  |  |
| 1443575_at | 2310040G24Rik | RIKEN cDNA 2310040G24 gene |  |  |  |  |  |  |
| 1458587_at | 2310047D07Rik | RIKEN cDNA 2310047D07 gene |  |  |  |  |  |  |
| 1431764_at | 2310067P03Rik | RIKEN cDNA 2310067P03 gene |  |  |  |  |  |  |
| 1432651_at | 2510019K15Rik | RIKEN cDNA 2510019K15 gene |  |  |  |  |  |  |
| 1433051_at | 2610011I18Rik | RIKEN cDNA 2610011I18 gene |  |  |  |  |  |  |
| 1447695_at | 2610035D17Rik | RIKEN cDNA 2610035D17 gene |  |  |  |  |  |  |
| 1431906_at | 2700068H02Rik | RIKEN cDNA 2700068H02 gene |  |  |  |  |  |  |
| 1457130_at | 2900035I09Rik | RIKEN cDNA 2900035I09 gene |  |  |  |  |  |  |
| 1454328_at | 2900060K15Rik | RIKEN cDNA 2900060K15 gene |  |  |  |  |  |  |
| 1432582_at | 3110054G05Rik | RIKEN cDNA 3110054G05 gene |  |  |  |  |  |  |
| 1432919_at | 4921511E18Rik | RIKEN cDNA 4921511E18 gene |  |  |  |  |  |  |
| 1432971_at | 4921518B13Rik | RIKEN cDNA 4921518B13 gene |  |  |  |  |  |  |
| 1447157_at | 4930480K23Rik | RIKEN cDNA 4930480K23 gene |  |  |  |  |  |  |
| 1432328_at | 4930516K23Rik | RIKEN cDNA 4930516K23 gene |  |  |  |  |  |  |
| 1433361_at | 4930527F18Rik | RIKEN cDNA 4930527F18 gene |  |  |  |  |  |  |
| 1433244_at | 4930573G07Rik | RIKEN cDNA 4930573G07 gene |  |  |  |  |  |  |
| 1432368_at | 4930594M17Rik | RIKEN cDNA 4930594M17 gene |  |  |  |  |  |  |
| 1433057_at | 4933411O13Rik | RIKEN cDNA 4933411O13 gene |  |  |  |  |  |  |
| 1445858_at | 4933439G19Rik | RIKEN cDNA 4933439G19 gene |  |  |  |  |  |  |
| 1433126_at | 5330422M15Rik | RIKEN cDNA 5330422M15 gene |  |  |  |  |  |  |
| 1435830_a_at | 5430435G22Rik | RIKEN cDNA 5430435G22 gene |  |  |  |  |  |  |
| 1430470_at | 5730411F24Rik | RIKEN cDNA 5730411F24 gene |  |  |  |  |  |  |
| 1429896_at | 5830408B19Rik | RIKEN cDNA 5830408B19 gene |  |  |  |  |  |  |
| 1432570_at | 6030458E02Rik | RIKEN cDNA 6030458E02 gene |  |  |  |  |  |  |
| 1454897_at | 6330509M05Rik | RIKEN cDNA 6330509M05 gene |  |  |  |  |  |  |
| 1454343_at | 6430710M23Rik | RIKEN cDNA 6430710M23 gene |  |  |  |  |  |  |
| 1431657_at | 9230112D13Rik | RIKEN cDNA 9230112D13 gene |  |  |  |  |  |  |
| 1454587_at | 9330199C07Rik | RIKEN cDNA 9330199C07 gene |  |  |  |  |  |  |
| 1430730_at | 9430024F10Rik | RIKEN cDNA 9430024F10 gene |  |  |  |  |  |  |
| 1433196_at | 9530006O14Rik | RIKEN cDNA 9530006O14 gene |  |  |  |  |  |  |
| 1435584_at | A630033H20Rik | RIKEN cDNA A630033H20 gene |  |  |  |  |  |  |
| 1429890_at | A930007D18Rik | RIKEN cDNA A930007D18 gene |  |  |  |  |  |  |
| 1437441_at | AA388235 | expressed sequence AA388235 |  |  |  |  |  |  |
| 1449781_at | AA517650 | expressed sequence AA517650 |  |  |  |  |  |  |
| 1451377_a_at | AAAS | achalasia, adrenocortical insufficiency, alacrimia | |  |  |  |  |  |
| 1422226_at | ABAT | 4-aminobutyrate aminotransferase |  |  |  |  |  |  |
| 1455328_at | ACCN2 | amiloride-sensitive cation channel 2, neuronal | |  |  |  |  |  |
| 1422925_s_at | ACOT1 | acyl-CoA thioesterase 1 |  |  |  |  |  |  |
| 1455720_at | ADAMTS2 | ADAM metallopeptidase with thrombospondin type 1 motif, 2 | | |  |  |  |  |
| 1421300_at | ADARB2 | adenosine deaminase, RNA-specific, B2 |  |  |  |  |  |  |
| 1418586_at | ADCY9 | adenylate cyclase 9 |  |  |  |  |  |  |
| 1427519_at | ADORA2A | adenosine A2a receptor |  |  |  |  |  |  |
| 1435838_at | AGXT2 | alanine--glyoxylate aminotransferase 2 |  |  |  |  |  |  |
| 1443565_at | AI849538 | expressed sequence AI849538 |  |  |  |  |  |  |
| 1435049_s_at | AI854703 | expressed sequence AI854703 |  |  |  |  |  |  |
| 1424262_at | AIF1L | allograft inflammatory factor 1-like |  |  |  |  |  |  |
| 1439254_at | AKAP13 | A kinase (PRKA) anchor protein 13 |  |  |  |  |  |  |
| 1421791_at | Ang2 (includes others) | angiogenin, ribonuclease A family, member 2 | |  |  |  |  |  |
| 1421002_at | ANGPTL2 | angiopoietin-like 2 |  |  |  |  |  |  |
| 1425482_s_at | ANKMY2 | ankyrin repeat and MYND domain containing 2 | |  |  |  |  |  |
| 1451554_a_at | Aph1a | anterior pharynx defective 1a homolog (C. elegans) | |  |  |  |  |  |
| 1418278_at | APOC3 | apolipoprotein C-III |  |  |  |  |  |  |
| 1455522_at | ARHGEF15 | Rho guanine nucleotide exchange factor (GEF) 15 | |  |  |  |  |  |
| 1434409_at | ARMCX6 | armadillo repeat containing, X-linked 6 |  |  |  |  |  |  |
| 1444719_at | ASPH | aspartate beta-hydroxylase |  |  |  |  |  |  |
| 1452732_at | ASPRV1 | aspartic peptidase, retroviral-like 1 |  |  |  |  |  |  |
| 1441122_at | AU024342 | expressed sequence AU024342 |  |  |  |  |  |  |
| 1422279_at | AU040096/Fv1 | Friend virus susceptibility 1 |  |  |  |  |  |  |
| 1425752_at | BC014805 | cDNA sequence BC014805 |  |  |  |  |  |  |
| 1439055_at | BC099439 | transcription elongation factor B (SIII), polypeptide 2 pseudogene | | | |  |  |  |
| 1428773_s_at | BCOR | BCL6 corepressor |  |  |  |  |  |  |
| 1418271_at | BHLHE22 | basic helix-loop-helix family, member e22 | |  |  |  |  |  |
| 1420006_at | BMP15 | bone morphogenetic protein 15 |  |  |  |  |  |  |
| 1458386_at | C030009J22Rik | RIKEN cDNA C030009J22 gene |  |  |  |  |  |  |
| 1427176_s_at | C19orf46 | chromosome 19 open reading frame 46 |  |  |  |  |  |  |
| 1427041_at | C1orf38 | chromosome 1 open reading frame 38 |  |  |  |  |  |  |
| 1457584_at | C1orf9 | chromosome 1 open reading frame 9 |  |  |  |  |  |  |
| 1456364_at | C230057M02Rik | RIKEN cDNA C230057M02 gene |  |  |  |  |  |  |
| 1457878_at | C430042M11Rik | RIKEN cDNA C430042M11 gene |  |  |  |  |  |  |
| 1439689_at | C6orf186 | chromosome 6 open reading frame 186 |  |  |  |  |  |  |
| 1449765_at | C76628 | expressed sequence C76628 |  |  |  |  |  |  |
| 1458935_at | C79744 | expressed sequence C79744 |  |  |  |  |  |  |
| 1457475_at | C80993 | expressed sequence C80993 |  |  |  |  |  |  |
| 1439198_at | C85445 | expressed sequence C85445 |  |  |  |  |  |  |
| 1460256_at | CA3 | carbonic anhydrase III, muscle specific |  |  |  |  |  |  |
| 1427330_at | CARS | cysteinyl-tRNA synthetase |  |  |  |  |  |  |
| 1450797_a_at | CBX1 | chromobox homolog 1 |  |  |  |  |  |  |
| 1430090_at | CCDC28B | coiled-coil domain containing 28B |  |  |  |  |  |  |
| 1422029_at | CCL20 | chemokine (C-C motif) ligand 20 |  |  |  |  |  |  |
| 1436065_at | CCR9 | chemokine (C-C motif) receptor 9 |  |  |  |  |  |  |
| 1418815_at | CDH2 | cadherin 2, type 1, N-cadherin (neuronal) | |  |  |  |  |  |
| 1457001_at | CENPK | centromere protein K |  |  |  |  |  |  |
| 1444641_at | CENPO | centromere protein O |  |  |  |  |  |  |
| 1436428_at | CHRNB2 | cholinergic receptor, nicotinic, beta 2 (neuronal) | |  |  |  |  |  |
| 1438261_at | CITED4 | Cbp/p300-interacting transactivator, with Glu/Asp-rich carboxy-terminal domain, 4 | | | | |  |  |
| 1427312_at | Cmya5 | cardiomyopathy associated 5 |  |  |  |  |  |  |
| 1452981_at | CNTN1 | contactin 1 |  |  |  |  |  |  |
| 1434262_at | COG1 | component of oligomeric golgi complex 1 |  |  |  |  |  |  |
| 1436613_at | CORO6 | coronin 6 |  |  |  |  |  |  |
| 1416002_x_at | COTL1 | coactosin-like 1 (Dictyostelium) |  |  |  |  |  |  |
| 1429443_at | CPNE4 | copine IV |  |  |  |  |  |  |
| 1459589_at | CRYL1 | crystallin, lambda 1 |  |  |  |  |  |  |
| 1421449_at | CSMD1 | CUB and Sushi multiple domains 1 |  |  |  |  |  |  |
| 1435373_at | CSNK1E | casein kinase 1, epsilon |  |  |  |  |  |  |
| 1436779_at | CYBB | cytochrome b-245, beta polypeptide |  |  |  |  |  |  |
| 1422217_a_at | CYP1A1 | cytochrome P450, family 1, subfamily A, polypeptide 1 | | |  |  |  |  |
| 1419582_at | CYP2C18 | cytochrome P450, family 2, subfamily C, polypeptide 18 | | |  |  |  |  |
| 1457626_at | D3Wsu106e | DNA segment, Chr 3, Wayne State University 106, expressed | | |  |  |  |  |
| 1423872_a_at | DAG1 | dystroglycan 1 (dystrophin-associated glycoprotein 1) | | |  |  |  |  |
| 1434519_at | DDAH1 | dimethylarginine dimethylaminohydrolase 1 | |  |  |  |  |  |
| 1430050_at | DDX23 | DEAD (Asp-Glu-Ala-Asp) box polypeptide 23 | |  |  |  |  |  |
| 1418550_x_at | Defa-rs1 (includes others) (mouse) | defensin, alpha, related sequence 1 |  |  |  |  |  |  |
| 1432891_at | DHRS7 | dehydrogenase/reductase (SDR family) member 7 | |  |  |  |  |  |
| 1444395_at | DIXDC1 | DIX domain containing 1 |  |  |  |  |  |  |
| 1431186_at | DLG5 | discs, large homolog 5 (Drosophila) |  |  |  |  |  |  |
| 1420219_at | DNAJC21 | DnaJ (Hsp40) homolog, subfamily C, member 21 | |  |  |  |  |  |
| 1452097_a_at | DUSP7 | dual specificity phosphatase 7 |  |  |  |  |  |  |
| 1458724_at | E230008O15Rik | RIKEN cDNA E230008O15 gene |  |  |  |  |  |  |
| 1455790_at | E2F2 | E2F transcription factor 2 |  |  |  |  |  |  |
| 1419597_at | EDA | ectodysplasin A |  |  |  |  |  |  |
| 1433133_at | EDARADD | EDAR-associated death domain |  |  |  |  |  |  |
| 1438124_at | EG224552 (includes others) | vomeronasal 2, receptor 107 |  |  |  |  |  |  |
| 1457143_at | EG625121 | predicted gene 6556 |  |  |  |  |  |  |
| 1451699_at | EG668468 | RIKEN cDNA B230354O11 gene |  |  |  |  |  |  |
| 1417977_at | EIF4E3 | eukaryotic translation initiation factor 4E family member 3 | | |  |  |  |  |
| 1455575_at | EIF4EBP2 | eukaryotic translation initiation factor 4E binding protein 2 | | |  |  |  |  |
| 1445521_at | ELAVL1 | ELAV (embryonic lethal, abnormal vision, Drosophila)-like 1 (Hu antigen R) | | | | |  |  |
| 1434083_a_at | ELMOD1 | ELMO/CED-12 domain containing 1 |  |  |  |  |  |  |
| 1442656_at | ELOVL6 | ELOVL family member 6, elongation of long chain fatty acids (FEN1/Elo2, SUR4/Elo3-like, yeast) | | | | | | |
| 1451099_at | ESYT1 | extended synaptotagmin-like protein 1 |  |  |  |  |  |  |
| 1446891_at | FAM117B | family with sequence similarity 117, member B | |  |  |  |  |  |
| 1436403_at | FAM171A2 | family with sequence similarity 171, member A2 | |  |  |  |  |  |
| 1441095_at | FAM179B | family with sequence similarity 179, member B | |  |  |  |  |  |
| 1417688_at | FAM20C | family with sequence similarity 20, member C | |  |  |  |  |  |
| 1457368_at | FAM65C | family with sequence similarity 65, member C | |  |  |  |  |  |
| 1449141_at | FBLIM1 | filamin binding LIM protein 1 |  |  |  |  |  |  |
| 1448620_at | FCGR2A | Fc fragment of IgG, low affinity IIa, receptor (CD32) | | |  |  |  |  |
| 1420765_a_at | FOXP3 | forkhead box P3 |  |  |  |  |  |  |
| 1450319_at | GABRB2 | gamma-aminobutyric acid (GABA) A receptor, beta 2 | | |  |  |  |  |
| 1420197_at | GADD45B | growth arrest and DNA-damage-inducible, beta | |  |  |  |  |  |
| 1439615_at | GAN | gigaxonin |  |  |  |  |  |  |
| 1425579_at | GFRA2 | GDNF family receptor alpha 2 |  |  |  |  |  |  |
| 1451856_at | GIGYF2 | GRB10 interacting GYF protein 2 |  |  |  |  |  |  |
| 1429101_at | GJA5 | gap junction protein, alpha 5, 40kDa |  |  |  |  |  |  |
| 1437139_at | GLRA1 | glycine receptor, alpha 1 |  |  |  |  |  |  |
| 1424825_a_at | GLYCAM1 | glycosylation dependent cell adhesion molecule 1 (pseudogene) | | | |  |  |  |
| 1439816_at | Gm10567 | predicted gene 10567 |  |  |  |  |  |  |
| 1441759_at | Gm10804 | predicted gene 10804 |  |  |  |  |  |  |
| 1426101_at | Gm10997 | predicted gene 10997 |  |  |  |  |  |  |
| 1419901_at | Gm11110 | predicted gene 11110 |  |  |  |  |  |  |
| 1444524_at | Gm14005 | predicted gene 14005 |  |  |  |  |  |  |
| 1445687_at | Gm885 | predicted gene 885 |  |  |  |  |  |  |
| 1444781_at | GPN1 | GPN-loop GTPase 1 |  |  |  |  |  |  |
| 1437356_at | GPR183 | G protein-coupled receptor 183 |  |  |  |  |  |  |
| 1459245_s_at | GRID2 | glutamate receptor, ionotropic, delta 2 |  |  |  |  |  |  |
| 1448273_at | GSS | glutathione synthetase |  |  |  |  |  |  |
| 1459522_s_at | GYG1 | glycogenin 1 |  |  |  |  |  |  |
| 1453574_at | HBA1/HBA2 | hemoglobin, alpha 1 |  |  |  |  |  |  |
| 1420604_at | HESX1 | HESX homeobox 1 |  |  |  |  |  |  |
| 1419297_at | HLA-DOA | major histocompatibility complex, class II, DO alpha | |  |  |  |  |  |
| 1425996_a_at | HLTF | helicase-like transcription factor |  |  |  |  |  |  |
| 1418693_at | HNRNPC | heterogeneous nuclear ribonucleoprotein C (C1/C2) | | |  |  |  |  |
| 1420414_at | HOXA11 | homeobox A11 |  |  |  |  |  |  |
| 1425833_a_at | HPCA | hippocalcin |  |  |  |  |  |  |
| 1423028_at | IFNA1/IFNA13 | interferon, alpha 1 |  |  |  |  |  |  |
| 1422313_a_at | IGFBP5 | insulin-like growth factor binding protein 5 | |  |  |  |  |  |
| 1425738_at | Igk-v21-7 | immunoglobulin kappa variable 3-7 |  |  |  |  |  |  |
| 1426507_at | IL1F5 | interleukin 1 family, member 5 (delta) |  |  |  |  |  |  |
| 1451983_at | IRX1 | iroquois homeobox 1 |  |  |  |  |  |  |
| 1423608_at | ITM2A | integral membrane protein 2A |  |  |  |  |  |  |
| 1429796_at | KALRN | kalirin, RhoGEF kinase |  |  |  |  |  |  |
| 1431043_at | KBTBD5 | kelch repeat and BTB (POZ) domain containing 5 | |  |  |  |  |  |
| 1448468_a_at | KCNAB1 | potassium voltage-gated channel, shaker-related subfamily, beta member 1 | | | | |  |  |
| 1426080_a_at | KCNQ2 | potassium voltage-gated channel, KQT-like subfamily, member 2 | | | |  |  |  |
| 1455923_at | KCTD8 | potassium channel tetramerisation domain containing 8 | | |  |  |  |  |
| 1449936_at | KIAA1467 | KIAA1467 |  |  |  |  |  |  |
| 1454998_at | KIAA1704 | KIAA1704 |  |  |  |  |  |  |
| 1457046_s_at | KIAA2022 | KIAA2022 |  |  |  |  |  |  |
| 1426325_at | KIF1C | kinesin family member 1C |  |  |  |  |  |  |
| 1423015_at | KIRREL | kin of IRRE like (Drosophila) |  |  |  |  |  |  |
| 1425144_at | Klk1b1 (includes others) | kallikrein 1-related pepidase b4 |  |  |  |  |  |  |
| 1445399_at | Klrb1c (includes others) | killer cell lectin-like receptor subfamily B member 1C | | |  |  |  |  |
| 1456578_x_at | LASP1 | LIM and SH3 protein 1 |  |  |  |  |  |  |
| 1451018_at | LEPROTL1 | leptin receptor overlapping transcript-like 1 | |  |  |  |  |  |
| 1420643_at | LFNG | LFNG O-fucosylpeptide 3-beta-N-acetylglucosaminyltransferase | | | |  |  |  |
| 1437374_at | LOC100507290/ZNF865 | zinc finger protein 721-like |  |  |  |  |  |  |
| 1452424_at | LPAR4 | lysophosphatidic acid receptor 4 |  |  |  |  |  |  |
| 1440684_at | LPCAT2 | lysophosphatidylcholine acyltransferase 2 | |  |  |  |  |  |
| 1451245_at | LRRC3B | leucine rich repeat containing 3B |  |  |  |  |  |  |
| 1447954_at | LRRC49 | leucine rich repeat containing 49 |  |  |  |  |  |  |
| 1423843_at | LRRC61 | leucine rich repeat containing 61 |  |  |  |  |  |  |
| 1448487_at | Lrrfip1 | leucine rich repeat (in FLII) interacting protein 1 | |  |  |  |  |  |
| 1417110_at | MAN1A1 | mannosidase, alpha, class 1A, member 1 | |  |  |  |  |  |
| 1449693_at | MAP3K7 | mitogen-activated protein kinase kinase kinase 7 | |  |  |  |  |  |
| 1419677_at | Masp1 | mannan-binding lectin serine peptidase 1 | |  |  |  |  |  |
| 1457277_at | MBLAC1 | metallo-beta-lactamase domain containing 1 | |  |  |  |  |  |
| 1428765_at | Meg3 | maternally expressed 3 |  |  |  |  |  |  |
| 1440091_at | MEIS2 | Meis homeobox 2 |  |  |  |  |  |  |
| 1417595_at | MEOX1 | mesenchyme homeobox 1 |  |  |  |  |  |  |
| 1449952_s_at | MGC52282 | protease, serine, 30 homolog (mouse), pseudogene | |  |  |  |  |  |
| 1421898_a_at | MR1 | major histocompatibility complex, class I-related | |  |  |  |  |  |
| 1443264_at | MS4A2 | membrane-spanning 4-domains, subfamily A, member 2 (Fc fragment of IgE, high affinity I, receptor for; beta polypeptide) | | | | | | |
| 1417303_at | MVD | mevalonate (diphospho) decarboxylase |  |  |  |  |  |  |
| 1457126_at | MYL4 | myosin, light chain 4, alkali; atrial, embryonic | |  |  |  |  |  |
| 1460159_at | MYSM1 | Myb-like, SWIRM and MPN domains 1 |  |  |  |  |  |  |
| 1427434_at | Naip5 (includes others) | NLR family, apoptosis inhibitory protein 1 | |  |  |  |  |  |
| 1455320_at | NAMPT | nicotinamide phosphoribosyltransferase |  |  |  |  |  |  |
| 1421592_at | NCAM2 | neural cell adhesion molecule 2 |  |  |  |  |  |  |
| 1454809_at | NCOA7 | nuclear receptor coactivator 7 |  |  |  |  |  |  |
| 1459760_at | NDUFS4 | NADH dehydrogenase (ubiquinone) Fe-S protein 4, 18kDa (NADH-coenzyme Q reductase) | | | | | |  |
| 1439328_at | NFAT5 | nuclear factor of activated T-cells 5, tonicity-responsive | | |  |  |  |  |
| 1438236_at | NFIA | nuclear factor I/A |  |  |  |  |  |  |
| 1454834_at | NFIB | nuclear factor I/B |  |  |  |  |  |  |
| 1424731_at | NLE1 | notchless homolog 1 (Drosophila) |  |  |  |  |  |  |
| 1438086_at | NPY6R | neuropeptide Y receptor Y6 (pseudogene) | |  |  |  |  |  |
| 1416959_at | NR1D2 | nuclear receptor subfamily 1, group D, member 2 | |  |  |  |  |  |
| 1444229_at | NR2F2 | nuclear receptor subfamily 2, group F, member 2 | |  |  |  |  |  |
| 1430583_at | NRCAM | neuronal cell adhesion molecule |  |  |  |  |  |  |
| 1432603_at | NRIP1 | nuclear receptor interacting protein 1 |  |  |  |  |  |  |
| 1433792_at | NRIP2 | nuclear receptor interacting protein 2 |  |  |  |  |  |  |
| 1439358_a_at | NRXN1 | neurexin 1 |  |  |  |  |  |  |
| 1419710_at | NXPH3 | neurexophilin 3 |  |  |  |  |  |  |
| 1422367_at | Olfr70 | olfactory receptor 70 |  |  |  |  |  |  |
| 1440005_at | ONECUT2 | one cut homeobox 2 |  |  |  |  |  |  |
| 1422372_at | OR2C1 | olfactory receptor, family 2, subfamily C, member 1 | |  |  |  |  |  |
| 1425540_at | OTC | ornithine carbamoyltransferase |  |  |  |  |  |  |
| 1418252_at | PADI2 | peptidyl arginine deiminase, type II |  |  |  |  |  |  |
| 1420851_at | PARD6G | par-6 partitioning defective 6 homolog gamma (C. elegans) | | |  |  |  |  |
| 1452294_at | PCDH1 | protocadherin 1 |  |  |  |  |  |  |
| 1420429_at | PCDHB3 | protocadherin beta 3 |  |  |  |  |  |  |
| 1453636_at | PCGF5 | polycomb group ring finger 5 |  |  |  |  |  |  |
| 1452404_at | PHACTR2 | phosphatase and actin regulator 2 |  |  |  |  |  |  |
| 1436738_at | PIF1 | PIF1 5'-to-3' DNA helicase homolog (S. cerevisiae) | |  |  |  |  |  |
| 1443798_at | PIK3CD | phosphoinositide-3-kinase, catalytic, delta polypeptide | | |  |  |  |  |
| 1428025_s_at | PITPNC1 | phosphatidylinositol transfer protein, cytoplasmic 1 | |  |  |  |  |  |
| 1449586_at | PKP1 | plakophilin 1 (ectodermal dysplasia/skin fragility syndrome) | | |  |  |  |  |
| 1421170_a_at | PLCB1 | phospholipase C, beta 1 (phosphoinositide-specific) | |  |  |  |  |  |
| 1417237_at | PLD2 | phospholipase D2 |  |  |  |  |  |  |
| 1431081_a_at | PLSCR3 | phospholipid scramblase 3 |  |  |  |  |  |  |
| 1456731_x_at | POLR3K | polymerase (RNA) III (DNA directed) polypeptide K, 12.3 kDa | | |  |  |  |  |
| 1451210_at | PPAP2C | phosphatidic acid phosphatase type 2C |  |  |  |  |  |  |
| 1457578_at | PPARGC1B | peroxisome proliferator-activated receptor gamma, coactivator 1 beta | | | |  |  |  |
| 1441988_at | PPM1K | protein phosphatase, Mg2+/Mn2+ dependent, 1K | |  |  |  |  |  |
| 1445573_at | PPP1R14B | protein phosphatase 1, regulatory (inhibitor) subunit 14B | | |  |  |  |  |
| 1457197_at | PRKACB | protein kinase, cAMP-dependent, catalytic, beta | |  |  |  |  |  |
| 1427562_a_at | PRKCA | protein kinase C, alpha |  |  |  |  |  |  |
| 1449529_s_at | Prl7a1 | prolactin family 7, subfamily a, member 1 | |  |  |  |  |  |
| 1429818_at | PRR23A | proline rich 23A |  |  |  |  |  |  |
| 1420467_at | PSORS1C2 | psoriasis susceptibility 1 candidate 2 |  |  |  |  |  |  |
| 1431518_at | PTCHD3 | patched domain containing 3 |  |  |  |  |  |  |
| 1439984_at | PTDSS2 | phosphatidylserine synthase 2 |  |  |  |  |  |  |
| 1457493_at | PTEN | phosphatase and tensin homolog |  |  |  |  |  |  |
| 1453578_at | PTER | phosphotriesterase related |  |  |  |  |  |  |
| 1425251_at | PTGER3 | prostaglandin E receptor 3 (subtype EP3) | |  |  |  |  |  |
| 1443699_at | PTPRK | protein tyrosine phosphatase, receptor type, K | |  |  |  |  |  |
| 1417676_a_at | PTPRO | protein tyrosine phosphatase, receptor type, O | |  |  |  |  |  |
| 1443766_x_at | RAB11FIP4 | RAB11 family interacting protein 4 (class II) | |  |  |  |  |  |
| 1454988_s_at | RAB22A | RAB22A, member RAS oncogene family |  |  |  |  |  |  |
| 1449259_at | RAB3D | RAB3D, member RAS oncogene family |  |  |  |  |  |  |
| 1455924_at | RAB6B | RAB6B, member RAS oncogene family |  |  |  |  |  |  |
| 1443921_at | RANBP3L | RAN binding protein 3-like |  |  |  |  |  |  |
| 1427975_at | RASL10A | RAS-like, family 10, member A |  |  |  |  |  |  |
| 1450159_at | REM1 | RAS (RAD and GEM)-like GTP-binding 1 |  |  |  |  |  |  |
| 1425564_at | REST | RE1-silencing transcription factor |  |  |  |  |  |  |
| 1457009_at | RHOBTB3 | Rho-related BTB domain containing 3 |  |  |  |  |  |  |
| 1434741_at | RREB1 | ras responsive element binding protein 1 |  |  |  |  |  |  |
| 1452118_at | RRP1B | ribosomal RNA processing 1 homolog B (S. cerevisiae) | | |  |  |  |  |
| 1449319_at | RSPO1 | R-spondin homolog (Xenopus laevis) |  |  |  |  |  |  |
| 1437991_x_at | Rusc1 | RUN and SH3 domain containing 1 |  |  |  |  |  |  |
| 1428176_at | S1PR2 | sphingosine-1-phosphate receptor 2 |  |  |  |  |  |  |
| 1444282_at | SAMD10 | sterile alpha motif domain containing 10 |  |  |  |  |  |  |
| 1420272_at | SAMHD1 | SAM domain and HD domain 1 |  |  |  |  |  |  |
| 1422194_at | SCN5A | sodium channel, voltage-gated, type V, alpha subunit | | |  |  |  |  |
| 1436573_at | SCRN3 | secernin 3 |  |  |  |  |  |  |
| 1439882_at | SEC23IP | SEC23 interacting protein |  |  |  |  |  |  |
| 1416644_a_at | SEMA3B | sema domain, immunoglobulin domain (Ig), short basic domain, secreted, (semaphorin) 3B | | | | | |  |
| 1425906_a_at | SEMA3E | sema domain, immunoglobulin domain (Ig), short basic domain, secreted, (semaphorin) 3E | | | | | |  |
| 1448395_at | SFRP1 | secreted frizzled-related protein 1 |  |  |  |  |  |  |
| 1421270_at | SH3RF1 | SH3 domain containing ring finger 1 |  |  |  |  |  |  |
| 1425845_a_at | SHOC2 | soc-2 suppressor of clear homolog (C. elegans) | |  |  |  |  |  |
| 1454359_at | SLC12A6 | solute carrier family 12 (potassium/chloride transporters), member 6 | | | |  |  |  |
| 1439384_at | SLC13A3 | solute carrier family 13 (sodium-dependent dicarboxylate transporter), member 3 | | | | |  |  |
| 1453056_at | SLC16A13 | solute carrier family 16, member 13 (monocarboxylic acid transporter 13) | | | |  |  |  |
| 1429727_at | SLC16A9 | solute carrier family 16, member 9 (monocarboxylic acid transporter 9) | | | |  |  |  |
| 1420966_at | SLC25A15 | solute carrier family 25 (mitochondrial carrier; ornithine transporter) member 15 | | | | |  |  |
| 1436865_at | SLC26A11 | solute carrier family 26, member 11 |  |  |  |  |  |  |
| 1428954_at | SLC9A3R2 | solute carrier family 9 (sodium/hydrogen exchanger), member 3 regulator 2 | | | | |  |  |
| 1427825_at | SLCO1B3 | solute carrier organic anion transporter family, member 1B3 | | |  |  |  |  |
| 1420884_at | SLN | sarcolipin |  |  |  |  |  |  |
| 1437311_at | Snhg11 (mouse) | small nucleolar RNA host gene 11 |  |  |  |  |  |  |
| 1458204_at | SPARC | secreted protein, acidic, cysteine-rich (osteonectin) | |  |  |  |  |  |
| 1450220_a_at | SPDEF | SAM pointed domain containing ets transcription factor | | |  |  |  |  |
| 1441226_at | SPON1 | spondin 1, extracellular matrix protein |  |  |  |  |  |  |
| 1451939_a_at | SRPX | sushi-repeat containing protein, X-linked |  |  |  |  |  |  |
| 1418946_at | ST3GAL1 | ST3 beta-galactoside alpha-2,3-sialyltransferase 1 | |  |  |  |  |  |
| 1439238_at | SUN2 | Sad1 and UNC84 domain containing 2 |  |  |  |  |  |  |
| 1423406_at | SV2A | synaptic vesicle glycoprotein 2A |  |  |  |  |  |  |
| 1460230_at | SYN2 | synapsin II |  |  |  |  |  |  |
| 1435310_at | SYN3 | synapsin III |  |  |  |  |  |  |
| 1444074_at | SYNE2 | spectrin repeat containing, nuclear envelope 2 | |  |  |  |  |  |
| 1450828_at | SYNPO2 | synaptopodin 2 |  |  |  |  |  |  |
| 1415845_at | SYT4 | synaptotagmin IV |  |  |  |  |  |  |
| 1423323_at | TACSTD2 | tumor-associated calcium signal transducer 2 | |  |  |  |  |  |
| 1444594_at | TBX3 | T-box 3 |  |  |  |  |  |  |
| 1429556_at | TEAD1 | TEA domain family member 1 (SV40 transcriptional enhancer factor) | | | |  |  |  |
| 1418136_at | TGFB1I1 | transforming growth factor beta 1 induced transcript 1 | | |  |  |  |  |
| 1436682_at | TMSB10/TMSB4X | thymosin beta 4, X-linked |  |  |  |  |  |  |
| 1452425_at | TNFRSF14 | tumor necrosis factor receptor superfamily, member 14 (herpesvirus entry mediator) | | | | |  |  |
| 1422721_at | TNK1 | tyrosine kinase, non-receptor, 1 |  |  |  |  |  |  |
| 1452772_at | TNKS2 | tankyrase, TRF1-interacting ankyrin-related ADP-ribose polymerase 2 | | | |  |  |  |
| 1448458_at | TOP2B | topoisomerase (DNA) II beta 180kDa |  |  |  |  |  |  |
| 1438808_at | TP53 | tumor protein p53 |  |  |  |  |  |  |
| 1434573_at | TRAF3IP3 | TRAF3 interacting protein 3 |  |  |  |  |  |  |
| 1418756_at | TRH | thyrotropin-releasing hormone |  |  |  |  |  |  |
| 1436533_at | TROVE2 | TROVE domain family, member 2 |  |  |  |  |  |  |
| 1437445_at | TRPM1 | transient receptor potential cation channel, subfamily M, member 1 | | | |  |  |  |
| 1426179_a_at | TWSG1 | twisted gastrulation homolog 1 (Drosophila) | |  |  |  |  |  |
| 1415862_at | TYRP1 | tyrosinase-related protein 1 |  |  |  |  |  |  |
| 1456065_at | UBASH3A | ubiquitin associated and SH3 domain containing A | |  |  |  |  |  |
| 1445885_at | UBE2D2 | ubiquitin-conjugating enzyme E2D 2 (UBC4/5 homolog, yeast) | | |  |  |  |  |
| 1418132_a_at | UBFD1 | ubiquitin family domain containing 1 |  |  |  |  |  |  |
| 1438165_x_at | VAT1 | vesicle amine transport protein 1 homolog (T. californica) | | |  |  |  |  |
| 1421272_at | VAV2 | vav 2 guanine nucleotide exchange factor | |  |  |  |  |  |
| 1439876_at | VTI1A | vesicle transport through interaction with t-SNAREs homolog 1A (yeast) | | | |  |  |  |
| 1456624_at | WIPI1 | WD repeat domain, phosphoinositide interacting 1 | |  |  |  |  |  |
| 1443621_at | XAF1 | XIAP associated factor 1 |  |  |  |  |  |  |
| 1434729_at | ZC4H2 | zinc finger, C4H2 domain containing |  |  |  |  |  |  |
| 1460076_x_at | ZFP3 | zinc finger protein 3 homolog (mouse) |  |  |  |  |  |  |
| 1431160_x_at | Zfp640 (includes others) | zinc finger protein 640 |  |  |  |  |  |  |
| 1432887_at | Zfp942 | zinc finger protein 942 |  |  |  |  |  |  |
| 1447959_at | ZNF398 | zinc finger protein 398 |  |  |  |  |  |  |
| 1456093_at | ZNF536 | zinc finger protein 536 |  |  |  |  |  |  |
