# Supplementary material for: Role of Gonadotropin Regulated Testicular RNA Helicase (GRTH/DDX25) on Polysomal Associated mRNAs in Mouse Testis
Source: PLoS One. 2012 Mar 30;7(3):e32470. doi: 10.1371/journal.pone.0032470 (PMC3316541; doi:10.1371/journal.pone.0032470)
Supplement: Table S6 — Differentially regulated genes in Leydig cells of GRTH KO compared to wild type adult mice. A. List of down-regulated genes (144) in Leydig cells of GRTH−/− compared to wild type mice. B. List of down-regulated genes (155) in Leydig cells of GRTH−/− compared to wild type mice. (DOCX) [file pone.0032470.s010.docx]

| **Table S6. Differentially regulated genes in Leydig cells of GRTH KO compared to wild type adult mice.**  **A. List of down-regulated genes (144) in Leydig cells of GRTH KO compared to wild type adult mice** | | | | | | | | | | |  |  |
| --- | --- | --- | --- | --- | --- | --- | --- | --- | --- | --- | --- | --- |
|  |  |  |  |  |  |  |  |  |  |  |  |  |
| **Affymetrix ID** | **Symbol** | **Entrez Gene Name** | |  |  |  |  |  |  |  |  |  |
| 1431103_at | 1700003P14Rik | RIKEN cDNA 1700003P14 gene | | | |  |  |  |  |  |  |  |
| 1430284_at | 1700008P02Rik | RIKEN cDNA 1700008P02 gene | | | |  |  |  |  |  |  |  |
| 1432505_at | 1700009J07Rik | RIKEN cDNA 1700009J07 gene | | |  |  |  |  |  |  |  |  |
| 1429142_at | 1700012A03Rik | RIKEN cDNA 1700012A03 gene | | | |  |  |  |  |  |  |  |
| 1420767_at | 1700019G17Rik/Cml2 | camello-like 2 | |  |  |  |  |  |  |  |  |  |
| 1457421_at | 1700063D05Rik | RIKEN cDNA 1700063D05 gene | | | |  |  |  |  |  |  |  |
| 1454355_at | 1810021M19Rik | RIKEN cDNA 1810021M19 gene | | | |  |  |  |  |  |  |  |
| 1447576_at | 2010001K21Rik | RIKEN cDNA 2010001K21 gene | | | |  |  |  |  |  |  |  |
| 1421489_a_at | 2010106E10Rik | RIKEN cDNA 2010106E10 gene | | | |  |  |  |  |  |  |  |
| 1432651_at | 2510019K15Rik | RIKEN cDNA 2510019K15 gene | | | |  |  |  |  |  |  |  |
| 1433152_at | 2900022B07Rik | RIKEN cDNA 2900022B07 gene | | | |  |  |  |  |  |  |  |
| 1454384_at | 2900057C01Rik | RIKEN cDNA 2900057C01 gene | | | |  |  |  |  |  |  |  |
| 1432299_at | 4921513I08Rik | RIKEN cDNA 4921513I08 gene | | |  |  |  |  |  |  |  |  |
| 1454050_at | 4921521D15Rik | RIKEN cDNA 4921521D15 gene | | | |  |  |  |  |  |  |  |
| 1431969_at | 4930402D18Rik | RIKEN cDNA 4930402D18 gene | | | |  |  |  |  |  |  |  |
| 1433419_at | 4930405A07Rik | RIKEN cDNA 4930405A07 gene | | | |  |  |  |  |  |  |  |
| 1430872_at | 4930412O13Rik | RIKEN cDNA 4930412O13 gene | | | |  |  |  |  |  |  |  |
| 1447157_at | 4930480K23Rik | RIKEN cDNA 4930480K23 gene | | | |  |  |  |  |  |  |  |
| 1440525_at | 4930516B21Rik | RIKEN cDNA 4930516B21 gene | | | |  |  |  |  |  |  |  |
| 1433218_at | 4930566N20Rik | RIKEN cDNA 4930566N20 gene | | | |  |  |  |  |  |  |  |
| 1454177_at | 4930597G03Rik | RIKEN cDNA 4930597G03 gene | | | |  |  |  |  |  |  |  |
| 1430958_at | 4933430N04Rik | RIKEN cDNA 4933430N04 gene | | | |  |  |  |  |  |  |  |
| 1431925_at | 4933433H22Rik | RIKEN cDNA 4933433H22 gene | | | |  |  |  |  |  |  |  |
| 1454474_at | 4933433M23Rik | RIKEN cDNA 4933433M23 gene | | | |  |  |  |  |  |  |  |
| 1432939_at | 5730405N03Rik | RIKEN cDNA 5730405N03 gene | | | |  |  |  |  |  |  |  |
| 1453561_x_at | 8430431K14Rik | RIKEN cDNA 8430431K14 gene | | | |  |  |  |  |  |  |  |
| 1431459_at | 9430013L14Rik | RIKEN cDNA 9430013L14 gene | | |  |  |  |  |  |  |  |  |
| 1422226_at | ABAT | 4-aminobutyrate aminotransferase | | | |  |  |  |  |  |  |  |
| 1425170_a_at | ADAM15 | ADAM metallopeptidase domain 15 | | | |  |  |  |  |  |  |  |
| 1421300_at | ADARB2 | adenosine deaminase, RNA-specific, B2 | | | |  |  |  |  |  |  |  |
| 1439649_at | ADC | arginine decarboxylase | | |  |  |  |  |  |  |  |  |
| 1427519_at | ADORA2A | adenosine A2a receptor | | |  |  |  |  |  |  |  |  |
| 1440697_at | AI746446 | expressed sequence AI746446 | | |  |  |  |  |  |  |  |  |
| 1424959_at | ANXA13 | annexin A13 | |  |  |  |  |  |  |  |  |  |
| 1444897_at | AU017263 | expressed sequence AU017263 | | |  |  |  |  |  |  |  |  |
| 1422279_at | AU040096/Fv1 | Friend virus susceptibility 1 | | |  |  |  |  |  |  |  |  |
| 1446116_at | B230378P21Rik | RIKEN cDNA B230378P21 gene | | | |  |  |  |  |  |  |  |
| 1444565_at | BB166591 | expressed sequence BB166591 | | |  |  |  |  |  |  |  |  |
| 1431160_x_at | BC099486 | RIKEN cDNA 6030426L16 gene | | |  |  |  |  |  |  |  |  |
| 1420235_at | BPHL | biphenyl hydrolase-like (serine hydrolase) | | | |  |  |  |  |  |  |  |
| 1430886_at | C10orf11 | chromosome 10 open reading frame 11 | | | |  |  |  |  |  |  |  |
| 1439723_at | C15orf42 | chromosome 15 open reading frame 42 | | | |  |  |  |  |  |  |  |
| 1430422_at | C20orf141 | chromosome 20 open reading frame 141 | | | |  |  |  |  |  |  |  |
| 1459404_at | C4orf49 | chromosome 4 open reading frame 49 | | | |  |  |  |  |  |  |  |
| 1450633_at | CALML5 | calmodulin-like 5 | |  |  |  |  |  |  |  |  |  |
| 1431778_at | CARS2 | cysteinyl-tRNA synthetase 2, mitochondrial (putative) | | | | |  |  |  |  |  |  |
| 1432568_at | CBY3 | chibby homolog 3 (Drosophila) | | |  |  |  |  |  |  |  |  |
| 1439109_at | CCDC68 | coiled-coil domain containing 68 | | | |  |  |  |  |  |  |  |
| 1445388_at | CD226 | CD226 molecule | |  |  |  |  |  |  |  |  |  |
| 1460415_a_at | CD40 | CD40 molecule, TNF receptor superfamily member 5 | | | | |  |  |  |  |  |  |
| 1436565_at | CEACAM3 | carcinoembryonic antigen-related cell adhesion molecule 3 | | | | | |  |  |  |  |  |
| 1427669_a_at | CIT | citron (rho-interacting, serine/threonine kinase 21) | | | | |  |  |  |  |  |  |
| 1444643_at | CKM | creatine kinase, muscle | | |  |  |  |  |  |  |  |  |
| 1420434_at | CLDN16 | claudin 16 | |  |  |  |  |  |  |  |  |  |
| 1419467_at | CLEC14A | C-type lectin domain family 14, member A | | | |  |  |  |  |  |  |  |
| 1425235_s_at | COL20A1 | collagen, type XX, alpha 1 | | |  |  |  |  |  |  |  |  |
| 1417607_at | COX6A2 | cytochrome c oxidase subunit VIa polypeptide 2 | | | | |  |  |  |  |  |  |
| 1418771_a_at | CPB2 | carboxypeptidase B2 (plasma) | | |  |  |  |  |  |  |  |  |
| 1427429_at | CSF2 | colony stimulating factor 2 (granulocyte-macrophage) | | | | |  |  |  |  |  |  |
| 1438304_at | CXXC4 | CXXC finger protein 4 | | |  |  |  |  |  |  |  |  |
| 1418821_at | Cyp2a12/Cyp2a22 | cytochrome P450, family 2, subfamily a, polypeptide 12 | | | | | |  |  |  |  |  |
| 1418263_at | DDX25 | DEAD (Asp-Glu-Ala-Asp) box polypeptide 25 | | | | |  |  |  |  |  |  |
| 1452546_x_at | Defb10/Defb11 | defensin beta 11 | |  |  |  |  |  |  |  |  |  |
| 1432196_a_at | DSCAML1 | Down syndrome cell adhesion molecule like 1 | | | | |  |  |  |  |  |  |
| 1450119_at | Dst | dystonin |  |  |  |  |  |  |  |  |  |  |
| 1453400_at | DYDC2 | DPY30 domain containing 2 | | |  |  |  |  |  |  |  |  |
| 1453619_at | EFCAB1 | EF-hand calcium binding domain 1 | | | |  |  |  |  |  |  |  |
| 1444010_at | EIF4E | eukaryotic translation initiation factor 4E | | | |  |  |  |  |  |  |  |
| 1431761_at | ENTPD4 | ectonucleoside triphosphate diphosphohydrolase 4 | | | | |  |  |  |  |  |  |
| 1430685_at | FAM131B | family with sequence similarity 131, member B | | | | |  |  |  |  |  |  |
| 1441095_at | FAM179B | family with sequence similarity 179, member B | | | | |  |  |  |  |  |  |
| 1417552_at | FAP | fibroblast activation protein, alpha | | | |  |  |  |  |  |  |  |
| 1454005_at | FMO2 | flavin containing monooxygenase 2 (non-functional) | | | | |  |  |  |  |  |  |
| 1420373_at | FOXJ2 | forkhead box J2 | |  |  |  |  |  |  |  |  |  |
| 1440765_at | FRAS1 | Fraser syndrome 1 | |  |  |  |  |  |  |  |  |  |
| 1457038_at | FREM2 | FRAS1 related extracellular matrix protein 2 | | | | |  |  |  |  |  |  |
| 1437139_at | GLRA1 | glycine receptor, alpha 1 | | |  |  |  |  |  |  |  |  |
| 1421693_a_at | GPR98 | G protein-coupled receptor 98 | | |  |  |  |  |  |  |  |  |
| 1446430_at | HECTD2 | HECT domain containing 2 | | |  |  |  |  |  |  |  |  |
| 1419576_at | HOXB13 | homeobox B13 | |  |  |  |  |  |  |  |  |  |
| 1425833_a_at | HPCA | hippocalcin | |  |  |  |  |  |  |  |  |  |
| 1449242_s_at | HRG | histidine-rich glycoprotein | | |  |  |  |  |  |  |  |  |
| 1449392_at | HSD17B1 | hydroxysteroid (17-beta) dehydrogenase 1 | | | |  |  |  |  |  |  |  |
| 1452108_at | IGF1R | insulin-like growth factor 1 receptor | | | |  |  |  |  |  |  |  |
| 1450704_at | IHH | Indian hedgehog | |  |  |  |  |  |  |  |  |  |
| 1425958_at | IL36G | interleukin 36, gamma | | |  |  |  |  |  |  |  |  |
| 1426080_a_at | KCNQ2 | potassium voltage-gated channel, KQT-like subfamily, member 2 | | | | | |  |  |  |  |  |
| 1449463_at | Klk1b1 (includes others) | kallikrein 1-related pepidase b4 | | |  |  |  |  |  |  |  |  |
| 1445399_at | Klrb1c (includes others) | killer cell lectin-like receptor subfamily B member 1C | | | | |  |  |  |  |  |  |
| 1427800_at | KRTAP19-1 | keratin associated protein 19-1 | | |  |  |  |  |  |  |  |  |
| 1444710_at | LOC100503197 | hypothetical LOC100503197 | | |  |  |  |  |  |  |  |  |
| 1456674_at | LOC100503583 | fibrinogen silencer-binding protein-like | | | |  |  |  |  |  |  |  |
| 1440967_at | LOC100504231 | hypothetical LOC100504231 | | |  |  |  |  |  |  |  |  |
| 1448998_at | LPO | lactoperoxidase | |  |  |  |  |  |  |  |  |  |
| 1438344_at | LPPR5 | lipid phosphate phosphatase-related protein type 5 | | | | |  |  |  |  |  |  |
| 1430057_s_at | LRRC57 | leucine rich repeat containing 57 | | | |  |  |  |  |  |  |  |
| 1453528_at | LTA4H | leukotriene A4 hydrolase | | |  |  |  |  |  |  |  |  |
| 1444073_at | MAF | v-maf musculoaponeurotic fibrosarcoma oncogene homolog (avian) | | | | | | |  |  |  |  |
| 1425393_a_at | MAP2K7 | mitogen-activated protein kinase kinase 7 | | | |  |  |  |  |  |  |  |
| 1458015_at | MEGF11 | multiple EGF-like-domains 11 | | |  |  |  |  |  |  |  |  |
| 1427631_x_at | Mup1 (includes others) | major urinary protein 1 | | |  |  |  |  |  |  |  |  |
| 1431813_at | MYO18B | myosin XVIIIB | |  |  |  |  |  |  |  |  |  |
| 1446407_at | MYSM1 | Myb-like, SWIRM and MPN domains 1 | | | |  |  |  |  |  |  |  |
| 1446189_at | Myst4 | MYST histone acetyltransferase monocytic leukemia 4 | | | | | |  |  |  |  |  |
| 1442918_at | NAV3 | neuron navigator 3 | |  |  |  |  |  |  |  |  |  |
| 1450977_s_at | NDRG1 | N-myc downstream regulated 1 | | |  |  |  |  |  |  |  |  |
| 1443277_at | NDST3 | N-deacetylase/N-sulfotransferase (heparan glucosaminyl) 3 | | | | | |  |  |  |  |  |
| 1448728_a_at | NFKBIZ | nuclear factor of kappa light polypeptide gene enhancer in B-cells inhibitor, zeta | | | | | | | |  |  |  |
| 1421181_at | Npcd | neuronal pentraxin chromo domain | | | |  |  |  |  |  |  |  |
| 1417986_at | NRARP | NOTCH-regulated ankyrin repeat protein | | | |  |  |  |  |  |  |  |
| 1439358_a_at | NRXN1 | neurexin 1 | |  |  |  |  |  |  |  |  |  |
| 1419327_at | PDXDC1 | pyridoxal-dependent decarboxylase domain containing 1 | | | | | |  |  |  |  |  |
| 1458473_at | POU6F2 | POU class 6 homeobox 2 | | |  |  |  |  |  |  |  |  |
| 1426621_a_at | PPP2R2B | protein phosphatase 2, regulatory subunit B, beta | | | | |  |  |  |  |  |  |
| 1458284_at | PTBP1 | polypyrimidine tract binding protein 1 | | | |  |  |  |  |  |  |  |
| 1442264_at | RASGRP2 | RAS guanyl releasing protein 2 (calcium and DAG-regulated) | | | | | |  |  |  |  |  |
| 1430978_at | RPS25 | ribosomal protein S25 | | |  |  |  |  |  |  |  |  |
| 1425840_a_at | SEMA3F | sema domain, immunoglobulin domain (Ig), short basic domain, secreted, (semaphorin) 3F | | | | | | | | |  |  |
| 1450336_at | SETD1A | SET domain containing 1A | | |  |  |  |  |  |  |  |  |
| 1420559_a_at | SHOX2 | short stature homeobox 2 | | |  |  |  |  |  |  |  |  |
| 1420334_at | SLC12A8 | solute carrier family 12 (potassium/chloride transporters), member 8 | | | | | | |  |  |  |  |
| 1433094_at | SLC1A2 | solute carrier family 1 (glial high affinity glutamate transporter), member 2 | | | | | | |  |  |  |  |
| 1430374_at | SLC25A37 | solute carrier family 25, member 37 | | | |  |  |  |  |  |  |  |
| 1460129_at | SLC6A2 | solute carrier family 6 (neurotransmitter transporter, noradrenalin), member 2 | | | | | | | |  |  |  |
| 1447483_s_at | Snhg7 | small nucleolar RNA host gene (non-protein coding) 7 | | | | |  |  |  |  |  |  |
| 1451835_at | SOX21 | SRY (sex determining region Y)-box 21 | | | |  |  |  |  |  |  |  |
| 1421504_at | SP4 | Sp4 transcription factor | | |  |  |  |  |  |  |  |  |
| 1459019_at | SPRN | shadow of prion protein homolog (zebrafish) | | | | |  |  |  |  |  |  |
| 1431719_a_at | SRGAP1 | SLIT-ROBO Rho GTPase activating protein 1 | | | | |  |  |  |  |  |  |
| 1454246_at | ST3GAL6 | ST3 beta-galactoside alpha-2,3-sialyltransferase 6 | | | | |  |  |  |  |  |  |
| 1422195_s_at | TBX15 | T-box 15 |  |  |  |  |  |  |  |  |  |  |
| 1449969_at | TMOD4 | tropomodulin 4 (muscle) | | |  |  |  |  |  |  |  |  |
| 1450798_at | TNXB | tenascin XB | |  |  |  |  |  |  |  |  |  |
| 1440696_at | TRNP1 | TMF1-regulated nuclear protein 1 | | | |  |  |  |  |  |  |  |
| 1432397_at | TSSK3 | testis-specific serine kinase 3 | | |  |  |  |  |  |  |  |  |
| 1437955_at | UBQLNL | ubiquilin-like | |  |  |  |  |  |  |  |  |  |
| 1428993_at | UCMA | upper zone of growth plate and cartilage matrix associated | | | | | |  |  |  |  |  |
| 1422136_at | UHMK1 | U2AF homology motif (UHM) kinase 1 | | | |  |  |  |  |  |  |  |
| 1440542_at | VRTN | vertebrae development homolog (pig) | | | |  |  |  |  |  |  |  |
| 1453258_at | Wbscr25 | Williams Beuren syndrome chromosome region 25 (human) | | | | | |  |  |  |  |  |
| 1425819_at | ZBTB7C | zinc finger and BTB domain containing 7C | | | |  |  |  |  |  |  |  |
| 1452275_at | ZFAND3 | zinc finger, AN1-type domain 3 | | |  |  |  |  |  |  |  |  |
| 1426614_at | ZMYND8 | zinc finger, MYND-type containing 8 | | | |  |  |  |  |  |  |  |
| 1437374_at | ZNF865 | zinc finger protein 865 | | |  |  |  |  |  |  |  |  |

| **B. List of up-regulated genes (155) in Leydig cells of GRTH KO compared to wild type adult mice** | | | | | | | | |  |  |
| --- | --- | --- | --- | --- | --- | --- | --- | --- | --- | --- |
|  |  |  |  |  |  |  |  |  |  |  |
| **Affymetrix ID** | **Entrez Gene Name** |  |  |  |  |  |  |  |  |  |
| 1430637_at | 2210016H18Rik | RIKEN cDNA 2210016H18 gene | | | |  |  |  |  |  |
| 1431764_at | 2310067P03Rik | RIKEN cDNA 2310067P03 gene | | | |  |  |  |  |  |
| 1436231_at | 2900052N01Rik | RIKEN cDNA 2900052N01 gene | | | |  |  |  |  |  |
| 1432368_at | 4930594M17Rik | RIKEN cDNA 4930594M17 gene | | | |  |  |  |  |  |
| 1436172_at | 9530028C05 | hypothetical protein 9530028C05 | | | |  |  |  |  |  |
| 1421839_at | ABCA1 | ATP-binding cassette, sub-family A (ABC1), member 1 | | | | | |  |  |  |
| 1427054_s_at | ABI3BP | ABI family, member 3 (NESH) binding protein | | | | |  |  |  |  |
| 1455061_a_at | ACAA2 | acetyl-CoA acyltransferase 2 | | |  |  |  |  |  |  |
| 1448987_at | ACADL | acyl-CoA dehydrogenase, long chain | | | |  |  |  |  |  |
| 1456728_x_at | ACO1 | aconitase 1, soluble | |  |  |  |  |  |  |  |
| 1416225_at | ADH1C | alcohol dehydrogenase 1C (class I), gamma polypeptide | | | | | |  |  |  |
| 1417208_at | AMACR | alpha-methylacyl-CoA racemase | | | |  |  |  |  |  |
| 1421002_at | ANGPTL2 | angiopoietin-like 2 | |  |  |  |  |  |  |  |
| 1424176_a_at | ANXA4 | annexin A4 | |  |  |  |  |  |  |  |
| 1417561_at | APOC1 | apolipoprotein C-I | |  |  |  |  |  |  |  |
| 1452732_at | ASPRV1 | aspartic peptidase, retroviral-like 1 | | | |  |  |  |  |  |
| 1427451_a_at | BC018473 | cDNA sequence BC018473 | | |  |  |  |  |  |  |
| 1425764_a_at | BCAT2 | branched chain amino-acid transaminase 2, mitochondrial | | | | | |  |  |  |
| 1427153_at | BCKDHB | branched chain keto acid dehydrogenase E1, beta polypeptide | | | | | |  |  |  |
| 1452257_at | BDH1 | 3-hydroxybutyrate dehydrogenase, type 1 | | | |  |  |  |  |  |
| 1437661_at | C16orf89 | chromosome 16 open reading frame 89 | | | |  |  |  |  |  |
| 1424768_at | Cald1 | caldesmon 1 | |  |  |  |  |  |  |  |
| 1424186_at | CCDC80 | coiled-coil domain containing 80 | | | |  |  |  |  |  |
| 1419684_at | CCL8 | chemokine (C-C motif) ligand 8 | | |  |  |  |  |  |  |
| 1448788_at | CD200 | CD200 molecule | |  |  |  |  |  |  |  |
| 1449918_at | Cd209f/Cd209g | CD209g antigen | |  |  |  |  |  |  |  |
| 1460218_at | CD52 | CD52 molecule | |  |  |  |  |  |  |  |
| 1425519_a_at | CD74 | CD74 molecule, major histocompatibility complex, class II invariant chain | | | | | | |  |  |
| 1418901_at | CEBPB | CCAAT/enhancer binding protein (C/EBP), beta | | | | |  |  |  |  |
| 1451322_at | CMBL | carboxymethylenebutenolidase homolog (Pseudomonas) | | | | | |  |  |  |
| 1448316_at | CMTM3 | CKLF-like MARVEL transmembrane domain containing 3 | | | | | |  |  |  |
| 1436759_x_at | CNN3 | calponin 3, acidic | |  |  |  |  |  |  |  |
| 1449154_at | COL11A1 | collagen, type XI, alpha 1 | | |  |  |  |  |  |  |
| 1426348_at | COL4A1 | collagen, type IV, alpha 1 | | |  |  |  |  |  |  |
| 1421449_at | CSMD1 | CUB and Sushi multiple domains 1 | | | |  |  |  |  |  |
| 1416382_at | CTSC | cathepsin C | |  |  |  |  |  |  |  |
| 1418365_at | CTSH | cathepsin H | |  |  |  |  |  |  |  |
| 1419209_at | CXCL2 | chemokine (C-X-C motif) ligand 2 | | | |  |  |  |  |  |
| 1422333_at | CYP21A2 | cytochrome P450, family 21, subfamily A, polypeptide 2 | | | | | |  |  |  |
| 1419582_at | CYP2C18 | cytochrome P450, family 2, subfamily C, polypeptide 18 | | | | | |  |  |  |
| 1426915_at | DAPK1 | death-associated protein kinase 1 | | | |  |  |  |  |  |
| 1418550_x_at | Defa-rs1 (includes others) (mouse) | defensin, alpha, related sequence 1 | | | |  |  |  |  |  |
| 1427946_s_at | DPYD | dihydropyrimidine dehydrogenase | | | |  |  |  |  |  |
| 1452341_at | ECHS1 | enoyl CoA hydratase, short chain, 1, mitochondrial | | | | |  |  |  |  |
| 1427183_at | EFEMP1 | EGF containing fibulin-like extracellular matrix protein 1 | | | | | |  |  |  |
| 1421090_at | EPB41L1 | erythrocyte membrane protein band 4.1-like 1 | | | | |  |  |  |  |
| 1416021_a_at | FABP5 | fatty acid binding protein 5 (psoriasis-associated) | | | | |  |  |  |  |
| 1448620_at | FCGR2A | Fc fragment of IgG, low affinity IIa, receptor (CD32) | | | | |  |  |  |  |
| 1449555_a_at | FETUB | fetuin B |  |  |  |  |  |  |  |  |
| 1417429_at | FMO1 | flavin containing monooxygenase 1 | | | |  |  |  |  |  |
| 1448700_at | G0S2 | G0/G1switch 2 | |  |  |  |  |  |  |  |
| 1450971_at | GADD45B | growth arrest and DNA-damage-inducible, beta | | | | |  |  |  |  |
| 1449526_a_at | GDPD3 | glycerophosphodiester phosphodiesterase domain containing 3 | | | | | |  |  |  |
| 1437756_at | Gimap9 | GTPase, IMAP family member 9 | | | |  |  |  |  |  |
| 1416592_at | GLRX | glutaredoxin (thioltransferase) | | |  |  |  |  |  |  |
| 1436530_at | Gm11428 | predicted gene 11428 | | |  |  |  |  |  |  |
| 1443995_at | Gm9 | predicted gene 9 | |  |  |  |  |  |  |  |
| 1417422_at | GNMT | glycine N-methyltransferase | | |  |  |  |  |  |  |
| 1439029_at | GPT2 | glutamic pyruvate transaminase (alanine aminotransferase) 2 | | | | | |  |  |  |
| 1418654_at | HAO2 | hydroxyacid oxidase 2 (long chain) | | | |  |  |  |  |  |
| 1416101_a_at | HIST1H1C | histone cluster 1, H1c | | |  |  |  |  |  |  |
| 1451784_x_at | HLA-C | major histocompatibility complex, class I, C | | | |  |  |  |  |  |
| 1435290_x_at | HLA-DQA1 | major histocompatibility complex, class II, DQ alpha 1 | | | | |  |  |  |  |
| 1417025_at | HLA-DRB1 (includes EG:100332219) | major histocompatibility complex, class II, DR beta 1 | | | | |  |  |  |  |
| 1423858_a_at | HMGCS2 | 3-hydroxy-3-methylglutaryl-CoA synthase 2 (mitochondrial) | | | | | |  |  |  |
| 1434642_at | HSD17B11 | hydroxysteroid (17-beta) dehydrogenase 11 | | | | |  |  |  |  |
| 1450010_at | HSD17B12 | hydroxysteroid (17-beta) dehydrogenase 12 | | | | |  |  |  |  |
| 1421671_at | HSD17B3 | hydroxysteroid (17-beta) dehydrogenase 3 | | | |  |  |  |  |  |
| 1448453_at | HSD3B2 | hydroxy-delta-5-steroid dehydrogenase, 3 beta- and steroid delta-isomerase 2 | | | | | | | |  |
| 1417013_at | HSPB8 | heat shock 22kDa protein 8 | | |  |  |  |  |  |  |
| 1419647_a_at | IER3 | immediate early response 3 | | |  |  |  |  |  |  |
| 1421551_s_at | Ifi202b | interferon activated gene 202B | | |  |  |  |  |  |  |
| 1425738_at | Igk-v21-7 | immunoglobulin kappa variable 3-7 | | | |  |  |  |  |  |
| 1419042_at | Iigp1/Iigp1b | interferon inducible GTPase 1 | | |  |  |  |  |  |  |
| 1420678_a_at | IL17RB | interleukin 17 receptor B | | |  |  |  |  |  |  |
| 1448950_at | IL1R1 | interleukin 1 receptor, type I | | |  |  |  |  |  |  |
| 1427771_x_at | ITGB1 | integrin, beta 1 (fibronectin receptor, beta polypeptide, antigen CD29 includes MDF2, MSK12) | | | | | | | | |
| 1425341_at | KCNK3 | potassium channel, subfamily K, member 3 | | | |  |  |  |  |  |
| 1448181_at | KLF15 | Kruppel-like factor 15 | | |  |  |  |  |  |  |
| 1428837_at | KLHL14 | kelch-like 14 (Drosophila) | | |  |  |  |  |  |  |
| 1423935_x_at | KRT14 | keratin 14 |  |  |  |  |  |  |  |  |
| 1417780_at | LASS4 | LAG1 homolog, ceramide synthase 4 | | | |  |  |  |  |  |
| 1448550_at | LBP | lipopolysaccharide binding protein | | | |  |  |  |  |  |
| 1418478_at | LMO1 | LIM domain only 1 (rhombotin 1) | | | |  |  |  |  |  |
| 1424400_a_at | LOC100047937 | 10-formyltetrahydrofolate dehydrogenase-like | | | | |  |  |  |  |
| 1431004_at | LOXL2 | lysyl oxidase-like 2 | |  |  |  |  |  |  |  |
| 1417290_at | LRG1 | leucine-rich alpha-2-glycoprotein 1 | | | |  |  |  |  |  |
| 1416053_at | LRRN1 | leucine rich repeat neuronal 1 | | |  |  |  |  |  |  |
| 1416930_at | LY6D | lymphocyte antigen 6 complex, locus D | | | |  |  |  |  |  |
| 1453304_s_at | LY6E | lymphocyte antigen 6 complex, locus E | | | |  |  |  |  |  |
| 1436996_x_at | Lyz1/Lyz2 | lysozyme 2 | |  |  |  |  |  |  |  |
| 1417110_at | MAN1A1 | mannosidase, alpha, class 1A, member 1 | | | |  |  |  |  |  |
| 1439380_x_at | Meg3 | maternally expressed 3 | | |  |  |  |  |  |  |
| 1417595_at | MEOX1 | mesenchyme homeobox 1 | | |  |  |  |  |  |  |
| 1435135_at | NCEH1 | neutral cholesterol ester hydrolase 1 | | | |  |  |  |  |  |
| 1421955_a_at | NEDD4 | neural precursor cell expressed, developmentally down-regulated 4 | | | | | | |  |  |
| 1422596_at | NKAIN4 | Na+/K+ transporting ATPase interacting 4 | | | |  |  |  |  |  |
| 1455796_x_at | OLFM1 | olfactomedin 1 | |  |  |  |  |  |  |  |
| 1426155_a_at | OSR2 | odd-skipped related 2 (Drosophila) | | | |  |  |  |  |  |
| 1418908_at | PAM | peptidylglycine alpha-amidating monooxygenase | | | | |  |  |  |  |
| 1420429_at | PCDHB3 | protocadherin beta 3 | |  |  |  |  |  |  |  |
| 1460570_at | PGBD5 | piggyBac transposable element derived 5 | | | |  |  |  |  |  |
| 1460194_at | PHYH | phytanoyl-CoA 2-hydroxylase | | |  |  |  |  |  |  |
| 1420859_at | PKIA | protein kinase (cAMP-dependent, catalytic) inhibitor alpha | | | | | |  |  |  |
| 1424937_at | PLIN5 | perilipin 5 |  |  |  |  |  |  |  |  |
| 1429514_at | PPAP2B | phosphatidic acid phosphatase type 2B | | | |  |  |  |  |  |
| 1431518_at | PTCHD3 | patched domain containing 3 | | |  |  |  |  |  |  |
| 1448816_at | PTGIS | prostaglandin I2 (prostacyclin) synthase | | | |  |  |  |  |  |
| 1417676_a_at | PTPRO | protein tyrosine phosphatase, receptor type, O | | | | |  |  |  |  |
| 1438055_at | RARRES1 | retinoic acid receptor responder (tazarotene induced) 1 | | | | | |  |  |  |
| 1448975_s_at | REN | renin |  |  |  |  |  |  |  |  |
| 1425566_at | REST | RE1-silencing transcription factor | | | |  |  |  |  |  |
| 1419247_at | RGS2 | regulator of G-protein signaling 2, 24kDa | | | |  |  |  |  |  |
| 1415965_at | SCD | stearoyl-CoA desaturase (delta-9-desaturase) | | | | |  |  |  |  |
| 1415823_at | Scd2 | stearoyl-Coenzyme A desaturase 2 | | | |  |  |  |  |  |
| 1448158_at | SDC1 | syndecan 1 | |  |  |  |  |  |  |  |
| 1439882_at | SEC23IP | SEC23 interacting protein | | |  |  |  |  |  |  |
| 1419100_at | SERPINA3 | serpin peptidase inhibitor, clade A (alpha-1 antiproteinase, antitrypsin), member 3 | | | | | | | |  |
| 1424923_at | Serpina3g (includes others) | serine (or cysteine) peptidase inhibitor, clade A, member 3G | | | | | |  |  |  |
| 1421564_at | Serpina3k (includes others) | serine (or cysteine) peptidase inhibitor, clade A, member 3K | | | | | |  |  |  |
| 1416318_at | SERPINB1 | serpin peptidase inhibitor, clade B (ovalbumin), member 1 | | | | | |  |  |  |
| 1416168_at | SERPINF1 | serpin peptidase inhibitor, clade F (alpha-2 antiplasmin, pigment epithelium derived factor), member 1 | | | | | | | | |
| 1416625_at | SERPING1 | serpin peptidase inhibitor, clade G (C1 inhibitor), member 1 | | | | | |  |  |  |
| 1436865_at | SLC26A11 | solute carrier family 26, member 11 | | | |  |  |  |  |  |
| 1454764_s_at | SLC38A1 | solute carrier family 38, member 1 | | | |  |  |  |  |  |
| 1418395_at | SLC47A1 | solute carrier family 47, member 1 | | | |  |  |  |  |  |
| 1439368_a_at | SLC9A3R2 | solute carrier family 9 (sodium/hydrogen exchanger), member 3 regulator 2 | | | | | | |  |  |
| 1420884_at | SLN | sarcolipin |  |  |  |  |  |  |  |  |
| 1453003_at | SORL1 | sortilin-related receptor, L(DLR class) A repeats containing | | | | | |  |  |  |
| 1450220_a_at | SPDEF | SAM pointed domain containing ets transcription factor | | | | | |  |  |  |
| 1453391_at | Speer7-ps1 | spermatogenesis associated glutamate (E)-rich protein 7, pseudogene 1 | | | | | | |  |  |
| 1427119_at | SPINK4 | serine peptidase inhibitor, Kazal type 4 | | | |  |  |  |  |  |
| 1421849_at | STAG2 | stromal antigen 2 | |  |  |  |  |  |  |  |
| 1420447_at | SULT1E1 | sulfotransferase family 1E, estrogen-preferring, member 1 | | | | | |  |  |  |
| 1428975_at | SUSD3 | sushi domain containing 3 | | |  |  |  |  |  |  |
| 1440762_at | SYN2 | synapsin II | |  |  |  |  |  |  |  |
| 1418744_s_at | TESC | tescalcin |  |  |  |  |  |  |  |  |
| 1422973_a_at | THRSP | thyroid hormone responsive | | |  |  |  |  |  |  |
| 1417162_at | TMBIM1 | transmembrane BAX inhibitor motif containing 1 | | | | |  |  |  |  |
| 1423909_at | TMEM176A | transmembrane protein 176A | | |  |  |  |  |  |  |
| 1422587_at | TMEM45A | transmembrane protein 45A | | |  |  |  |  |  |  |
| 1420725_at | TMLHE | trimethyllysine hydroxylase, epsilon | | | |  |  |  |  |  |
| 1416950_at | TNFAIP8 | tumor necrosis factor, alpha-induced protein 8 | | | | |  |  |  |  |
| 1446496_at | TRA2B | transformer 2 beta homolog (Drosophila) | | | |  |  |  |  |  |
| 1424653_at | TSPAN15 | tetraspanin 15 | |  |  |  |  |  |  |  |
| 1448276_at | TSPAN4 | tetraspanin 4 | |  |  |  |  |  |  |  |
| 1433460_at | TTC7B | tetratricopeptide repeat domain 7B | | | |  |  |  |  |  |
| 1426179_a_at | TWSG1 | twisted gastrulation homolog 1 (Drosophila) | | | |  |  |  |  |  |
| 1423968_at | UGT3A2 | UDP glycosyltransferase 3 family, polypeptide A2 | | | | |  |  |  |  |
| 1435065_x_at | VAV2 | vav 2 guanine nucleotide exchange factor | | | |  |  |  |  |  |
| 1448162_at | VCAM1 | vascular cell adhesion molecule 1 | | | |  |  |  |  |  |
| 1453593_at | VGLL3 | vestigial like 3 (Drosophila) | | |  |  |  |  |  |  |
| 1430024_at | YIPF6 | Yip1 domain family, member 6 | | |  |  |  |  |  |  |
| 1420816_at | YWHAG | tyrosine 3-monooxygenase/tryptophan 5-monooxygenase activation protein, gamma polypeptide | | | | | | | | |
| 1430961_at | ZNF292 | zinc finger protein 292 | | |  |  |  |  |  |  |
