# Supplementary material for: Role of Gonadotropin Regulated Testicular RNA Helicase (GRTH/DDX25) on Polysomal Associated mRNAs in Mouse Testis
Source: PLoS One. 2012 Mar 30;7(3):e32470. doi: 10.1371/journal.pone.0032470 (PMC3316541; doi:10.1371/journal.pone.0032470)
Supplement: Table S7 — List of differentially expressed genes associated with GRTH in polysome of the individual testicular cells. (DOC) [file pone.0032470.s011.doc]

| **Table S7. List of differentially expressed genes associated with GRTH in polysome of the individual testicular cells** | | | | | | | | | | | | | | | | | | | | | | |  | |  |
| --- | --- | --- | --- | --- | --- | --- | --- | --- | --- | --- | --- | --- | --- | --- | --- | --- | --- | --- | --- | --- | --- | --- | --- | --- | --- |
|  |  | |  | | |  |  | |  | | |  | | |  | | |  |  | |  |  | |  |  |
| **51 Common elements in "TpolyD", "SpD" and "GRTHIP":** | | | | | | | | | |  | | |  | | | **70 Common elements in "TpolyD", "RsD" and "GRTHIP":** | | | | | | | | |  |
|  | | | | | | | | | |  | | |  | | |  | | | | | | | | |  |
| 1700003P14Rik | |  | | | CHL1 | |  | |  | | |  | | |  | | | 1700003P14Rik | | BZW1 | |  | | Klk1b1 | |
| 1700008P02Rik | |  | | | CSNK1G2 | |  | |  | | |  | | |  | | | 1700008P02Rik | | C14orf148 | | | PPP2R2B | |  |
| 1700009J07Rik | |  | | | DDX25 | |  | |  | | |  | | |  | | | 1700009J07Rik | | C20orf141 | | | REEP6 | |  |
| 1700012A03Rik | |  | | | DYDC2 | |  | |  | | |  | | |  | | | 1700012A03Rik | | C20orf71 | |  | | SKP2 |  |
| 1700015G11Rik | |  | | | FSCN3 | |  | |  | | |  | | |  | | | 1700015G11Rik | | C2orf51 | |  | | SOCS7 |  |
| 1700029H14Rik | |  | | | GLUL | |  | |  | | |  | | |  | | | 1700019P21Rik | | C4orf36 | |  | | SPATA18 |  |
| 1700042G07Rik | |  | | | Klk1b1 (includes others) | | | | |  | | |  | | | 1700023I07Rik | | | | C7orf31 | |  | | SPATA3 |  |
| 1700052I22Rik | |  | | | LIPE | |  | |  | | |  | | |  | | | 1700024P04Rik | | C9orf50 | |  | | SPATC1 |  |
| 1700063D05Rik | |  | | | LOC73317 | | |  | | |  | | |  | | | 1700042G07Rik | | | CABS1 | |  | | TSSK3 |  |
| 1700080E11Rik | |  | | | LRRC57 | |  | |  | | |  | | |  | | | 1700052I22Rik | | CCDC54 | |  | | TSSK6 |  |
| 1700109H08Rik | |  | | | OAZ3 | |  | |  | | |  | | |  | | | 1700063D05Rik | | CCDC70 | |  | | UBE2G2 |  |
| 4930406D18Rik | |  | | | ODF1 | |  | |  | | |  | | |  | | | 1700080E11Rik | | CDRT4 | |  | | UBL4B |  |
| 4930412O13Rik | |  | | | OXCT2 | |  | |  | | |  | | |  | | | 1700109H08Rik | | CLMN | |  | | UBQLNL |  |
| ADC |  | |  | | | PPP2R2B |  | |  | | |  | | |  | | | 4921530L21Rik | | COL20A1 | |  | | UBR7 |  |
| AGPAT2 |  | |  | | | PRM2 |  | |  | | |  | | |  | | | 4930406D18Rik | | CSNK1G2 | |  | | Wbscr25 |  |
| ATL3 |  | |  | | | PRM3 |  | |  | | |  | | |  | | | 4930412O13Rik | | DDX25 | |  | | Lelp1 |  |
| C10orf62 |  | |  | | | SPATA18 |  | |  | | |  | | |  | | | 4930503B20Rik | | DYDC2 | |  | | LIPE |  |
| C14orf148 | |  | | | SPATA3 | |  | |  | | |  | | |  | | | 4930523O13Rik | | EIF4E | |  | | LOC100502820 | |
| C20orf141 | |  | | | ST6GALNAC2 | | |  | | |  | | |  | | | 4930549C01Rik | | | FSCN3 | |  | | LOC73317 | |
| C2orf51 |  | |  | | | TMEM225 | |  | | |  | | |  | | | ADC | |  | | GLRX2 |  | | LRRC57 |  |
| C4orf36 |  | |  | | | TNP2 |  | |  | | |  | | |  | | | AGPAT2 |  | | GLUL |  | | MBD2 |  |
| C7orf31 |  | |  | | | TSSK3 |  | |  | | |  | | |  | | | ATL3 |  | | HERPUD2 |  | | ODF1 |  |
| CABS1 |  | |  | | | TSSK6 |  | |  | | |  | | |  | | |  |  | | HSPA1L |  | | OXCT2 |  |
| CCDC54 |  | |  | | | UBQLNL |  | |  | | |  | | |  | | |  |  | | IL2RG |  | | PAQR5 |  |
| CCDC70 |  | |  | | | Wbscr25 |  | |  | | |  | | |  | | |  |  | |  |  | |  |  |
| CDRT4 |  | |  | | |  |  | |  | | |  | | |  | | |  |  | |  |  | |  |  |
|  |  | |  | | |  |  | |  | | |  | | |  | | |  |  | |  |  | |  |  |
| **18 Common elements in "TpolyD", "LcD" and "GRTHIP":** | | | | | | | | | |  | | |  | | | **3 Common elements in "TpolyU", "LcU" and "GRTHIP":** | | | | | | | | |  |
|  | | | | | | | | | |  | | |  | | |  | | | | | | | | |  |
| 1700003P14Rik | |  | | |  | |  | |  | | |  | | |  | | | FABP5 |  | |  |  | |  |  |
| 1700008P02Rik | |  | | |  | |  | |  | | |  | | |  | | | Scd2 |  | |  |  | |  |  |
| 1700009J07Rik | |  | | |  | |  | |  | | |  | | |  | | | SULT1E1 |  | |  |  | |  |  |
| 1700012A03Rik | |  | | |  | |  | |  | | |  | | |  | | |  |  | |  |  | |  |  |
| 1700063D05Rik | |  | | |  | |  | |  | | |  | | |  | | |  |  | |  |  | |  |  |
| 4930412O13Rik | |  | | |  | |  | |  | | |  | | |  | | |  |  | |  |  | |  |  |
| ADC |  | |  | | |  |  | |  | | |  | | |  | | |  |  | |  |  | |  |  |
| C20orf141 | |  | | |  | |  | |  | | |  | | |  | | |  |  | |  |  | |  |  |
| COL20A1 |  | |  | | |  |  | |  | | |  | | |  | | |  |  | |  |  | |  |  |
| DDX25 |  | |  | | |  |  | |  | | |  | | |  | | |  |  | |  |  | |  |  |
| DYDC2 |  | |  | | |  |  | |  | | |  | | |  | | |  |  | |  |  | |  |  |
| EIF4E |  | |  | | |  |  | |  | | |  | | |  | | |  |  | |  |  | |  |  |
| Klk1b1 (includes others) | | | |  | | |  | |  | | |  | | |  | | |  |  | |  |  | |  |  |
| LRRC57 |  | |  | | |  |  | |  | | |  | | |  | | |  |  | |  |  | |  |  |
| PPP2R2B |  | |  | | |  |  | |  | | |  | | |  | | |  |  | |  |  | |  |  |
| TSSK3 |  | |  | | |  |  | |  | | |  | | |  | | |  |  | |  |  | |  |  |
| UBQLNL |  | |  | | |  |  | |  | | |  | | |  | | |  |  | |  |  | |  |  |
| Wbscr25 |  | |  | | |  |  | |  | | |  | | |  | | |  |  | |  |  | |  |  |

TpolyD: Testicular polysomal down-regulated gene in GRTH KO mice

TpolyU: Testicular polysomal up-regulated gene in GRTH KO mice

GRTHIP: Polysomal mRNA immunoprecipitated by GRTH Ab in WT mice

SpD: Spermatocytes down-regulated mRNA in GRTH KO mice

RsD: Round spermatids down-regulated mRNA in GRTH KO mice

LcD: Leydig cells down-regulated mRNA in GRTH KO mice

LcU: Leydig cells up-regulated mRNA in GRTH KO mice
